# Supplementary material for: The variation and evolution of complete human centromeres
Source: Nature. Author manuscript; Available in PMC 2024 May 11. (PMC11062924; doi:10.1038/s41586-024-07278-3)
Supplement: Supplementary Information [file NIHMS1986935-supplement-Supplementary_Information.pdf]

nature portfolio

https://doi.org/10.1038/s41586-024-07278-3

# Supplementary information

# The variation and evolution of complete human centromeres

In the format provided by the authors and unedited

## SUPPLEMENTARY INFORMATION FOR:

# The variation and evolution of complete human centromeres

**This PDF file includes:**

1. **Supplementary Notes 1 and 2**
2. **Supplementary Figures 1-80**
3. **Supplementary Tables 1-11**
4. **References**

## SUPPLEMENTARY NOTES

**Supplementary Note 1. Isolation, immortalization, and karyotype analysis of the CHM1 cell line.** CHM1hTERT (abbr. CHM1) cells were originally isolated from a hydatidiform mole at Magee-Womens Hospital (Pittsburgh, PA) in 1981 and subsequently immortalized via transformation with human telomerase reverse transcriptase (hTERT) in 2001. Analysis of the CHM1 cell line has shown that it is primarily of European origin[1], similar to the CHM13 cell line that was collected and established around the same time[2]. To determine the karyotype of the CHM1 cell line, we used three orthogonal methods: DAPI staining, spectral karyotyping, and single-cell sequencing of template DNA strands (Strand-seq; **Supplementary Figs. 2 and 3**). All three methods indicate that the CHM1 cell population is biclonal, with approximately 71% of cells existing in a diploid or near-diploid state and approximately 29% of cells existing in a tetraploid or near-tetraploid state (**Supplementary Figs. 2a,b**). Both diploid/near-diploid and tetraploid/near-tetraploid cells have multiple chromosomal rearrangements, including a translocation between chromosomes 4q35.1 and 11q24.3 (**Supplementary Figs. 2c,d**), a translocation between chromosomes 16q23.3 to 17q25.3 (**Supplementary Figs. 2e,f**), and a loss of chromosome 17 or the chromosome 17 p-arm in a subset of cells (**Supplementary Fig. 2f**). The translocation between chromosomes 4q35.1/11q24.3 is accompanied by a complete deletion of *STOX2* and *ADAMTS15* and partial deletion of *ADAMTS8* (**Supplementary Fig. 3c**). *ADAMTS15* is predicted to act as a tumor suppressor gene in breast and colorectal cancer[3,4]. Additionally, the translocation between chromosomes 16q23.3 to 17q25.3 results in a novel gene fusion between *CDH13* and *RPTOR* (**Supplementary Fig. 3d**), which are both associated with cancer[5–8] and may contribute to the observed karyotype of the CHM1 cell line.

**Supplementary Note 2. Loss of two distinct chromosomal regions in the CHM1 cell line.** Mapping of native PacBio HiFi and ONT long-read sequencing data to the CHM1 centromere assemblies reveals a reduction in coverage on the p-arm proximal side of the chromosome 17 centromere (**Supplementary Fig. 5g**), consistent with the loss of the p-arm in a subset of cells (**Supplementary Fig. 2f**). It also reveals a reduction in coverage over a 631-kbp region in the *D13Z2* α-satellite higher-order repeat (HOR) array on chromosome 13 (**Supplementary Fig. 5c**), indicating this region is deleted in a subset of cells.

## SUPPLEMENTARY FIGURES

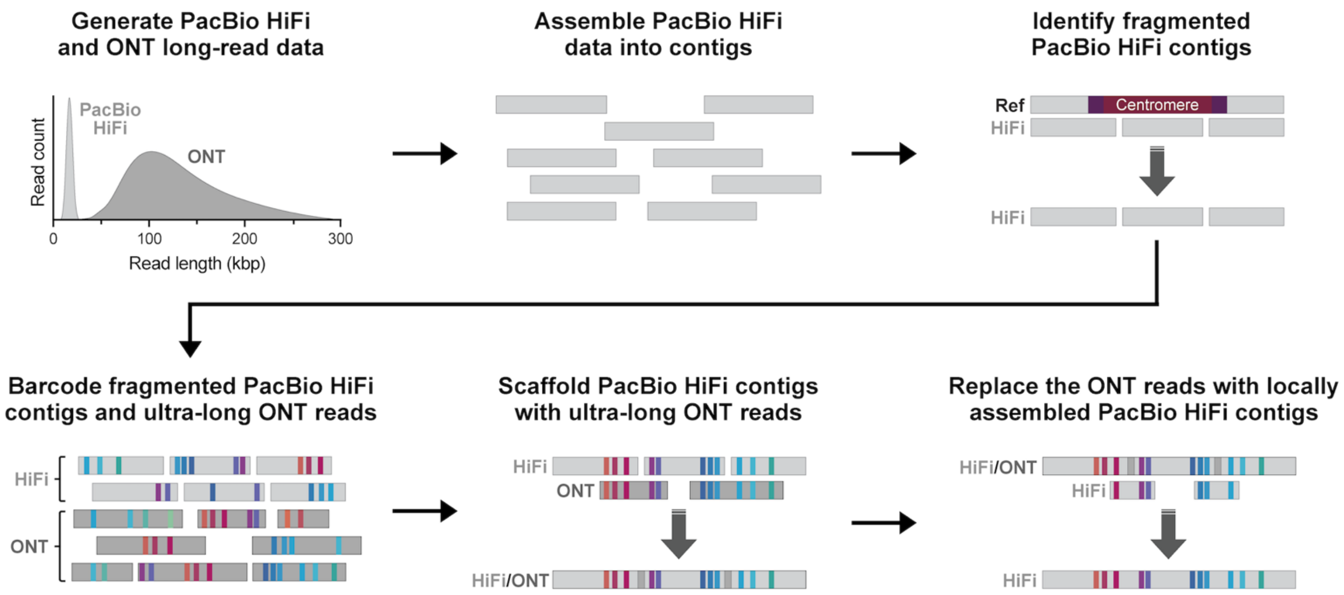


**Supplementary Figure 1. Centromere assembly method.** To assemble each CHM1 centromere, we first generated ~56-fold sequence coverage of Pacific Biosciences (PacBio) high-fidelity (HiFi) data and ~100-fold sequence coverage of Oxford Nanopore Technologies (ONT) data from the CHM1 genome. Then, we assembled the PacBio HiFi data into contigs using an established assembler, hifiasm[9]. We identified PacBio HiFi contigs that were fragmented over the centromeres by aligning them to the T2T-CHM13 reference genome. We barcoded the fragmented centromeric PacBio HiFi contigs and ultra-long (>100 kbp) ONT reads with singly unique nucleotide *k*-mers (SUNKs), creating unique SUNK barcodes. We ordered, oriented, and joined the PacBio HiFi contigs together with ultra-long ONT reads based on shared SUNK barcodes, generating a hybrid PacBio HiFi/ONT-based sequence assembly of each centromere. To improve the base accuracy of each assembly, we replaced the ONT reads with PacBio HiFi contigs that had been locally assembled with HiCanu[10], generating gapless sequence assemblies of each CHM1 centromere that are estimated to be >99.9999% accurate (as determined with Merqury[11]).

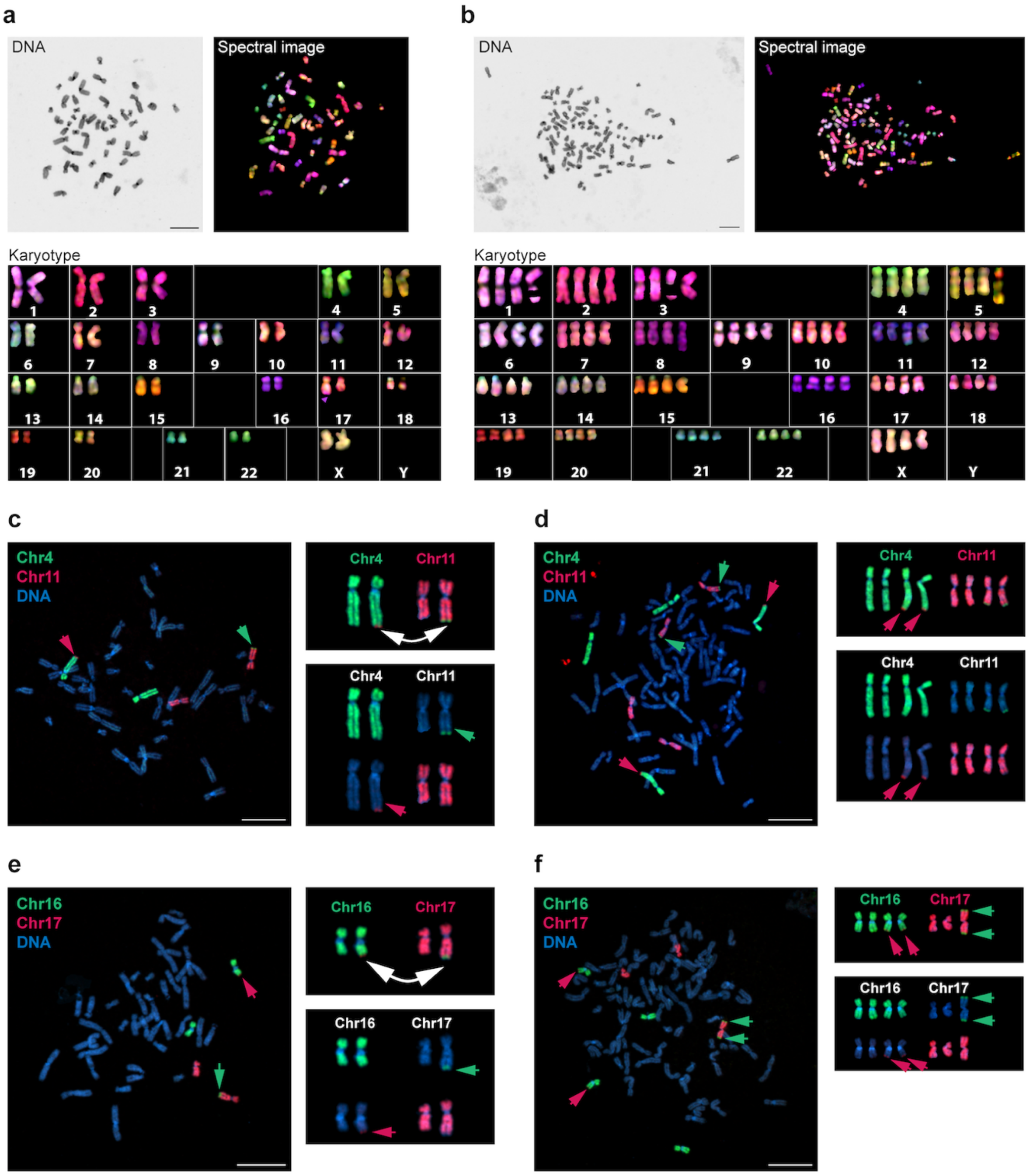


**Supplementary Figure 2. Karyotype of the CHM1 genome. a,b)** DAPI staining and spectral karyotyping of the CHM1 cell line reveals that approximately 71% of CHM1 cells are in a **a)** diploid/near-diploid state, and 29% of cells are in a **b)** tetraploid/near-tetraploid state. **c-f)** Fluorescent *in situ* hybridization on CHM1 metaphase chromosome spreads reveals that almost all cells have a reciprocal translocation between **c,d)** chromosomes 4q35.1 and 11q24.3 and **e,f)** chromosomes 16q23.3 to 17q25.3. Additionally, 44% of diploid/near-diploid cells and 83% of tetraploid/near-tetraploid cells are missing one copy of chromosome 17 or the chromosome 17 p-arm. Spectral karyotyping analysis was performed on two separate batches of CHM1 cells with similar results. n=24 and 22 metaphase chromosome spreads were assessed for translocations between chromosomes 4q35.1 and 11q24.3 and chromosomes 16q23.3 to 17q25.3, respectively. Bar, 10μm.

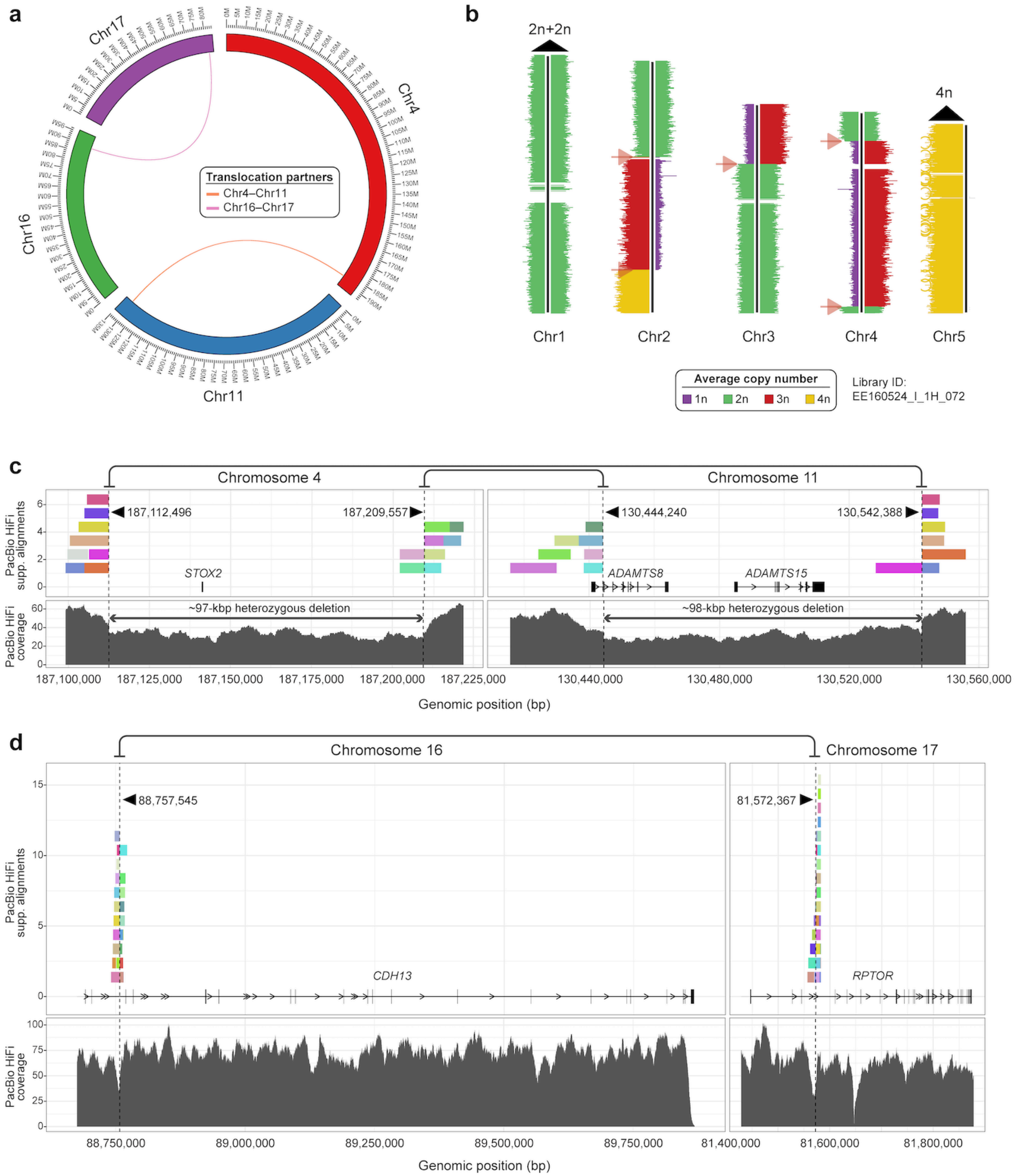


**Supplementary Figure 3. Translocations in the CHM1 genome. a)** Single-cell sequencing of template DNA strands (Strand-seq) from the CHM1 genome confirms the presence of two reciprocal translocations between chromosomes 4q35.1/11q24.3 and 16q23.3/17q25.3 and further refines the breakpoints to chr4:187112496/chr11:130542388, chr4:187209555/chr11:130444240, and chr16:88757545/chr17:81572367 (in T2T-CHM13 v2.0). We note that there are two breakpoints for the

chromosome 4q35.1/11q24.3 reciprocal translocation because it is accompanied by a ~97-98 kbp deletion at chr4:187112495-187209555 and chr11:130444240-130542388 (in T2T-CHM13 v2.0). **b)** Example of a CHM1 Strand-seq library mapped to a subset of chromosomes in the T2T-CHM13 reference genome[12]. Each chromosome is depicted as a vertical ideogram, and the distribution of directional sequencing reads is represented by horizontal lines along each chromosome. Reads mapped to the plus strand of the reference genome are shown on the left, and those mapped to the minus strand on the right of each ideogram. The average copy number of each chromosomal region is indicated. On chromosomes 1 and 5, for example, we find an average of 4n copies. On chromosomes 2, 3, and 4, we find low-frequency switches in strand-state (so-called sister-chromatid exchange events, or SCEs[13]) marked by arrows. Nevertheless, the overall copy number across each chromosome sums to 4n. **c, d)** Mapping of CHM1 PacBio HiFi reads to the T2T-CHM13 reference genome[12] reveals the precise breakpoints of the reciprocal translocation between chromosomes **c)** 4q35.1/11q24.3 and **d)** 16q23.3/17q25.3. CHM1 PacBio HiFi reads spanning the translocations are uniquely colored, and predicted translocation breakpoints are indicated with vertical dashed lines. The exon structure of all genes and the read depth of the CHM1 PacBio HiFi data are shown. The chromosome 4q35.1/11q24.3 translocation is associated with a deletion in both chromosomes, resulting in deletion of the *STOX2* and *ADAMTS15* genes and partial deletion of the *ADAMTS8* gene. The chromosome 16q23.3/17q25.3 is associated with a novel fusion of the *CDH13* and *RPTOR* genes.

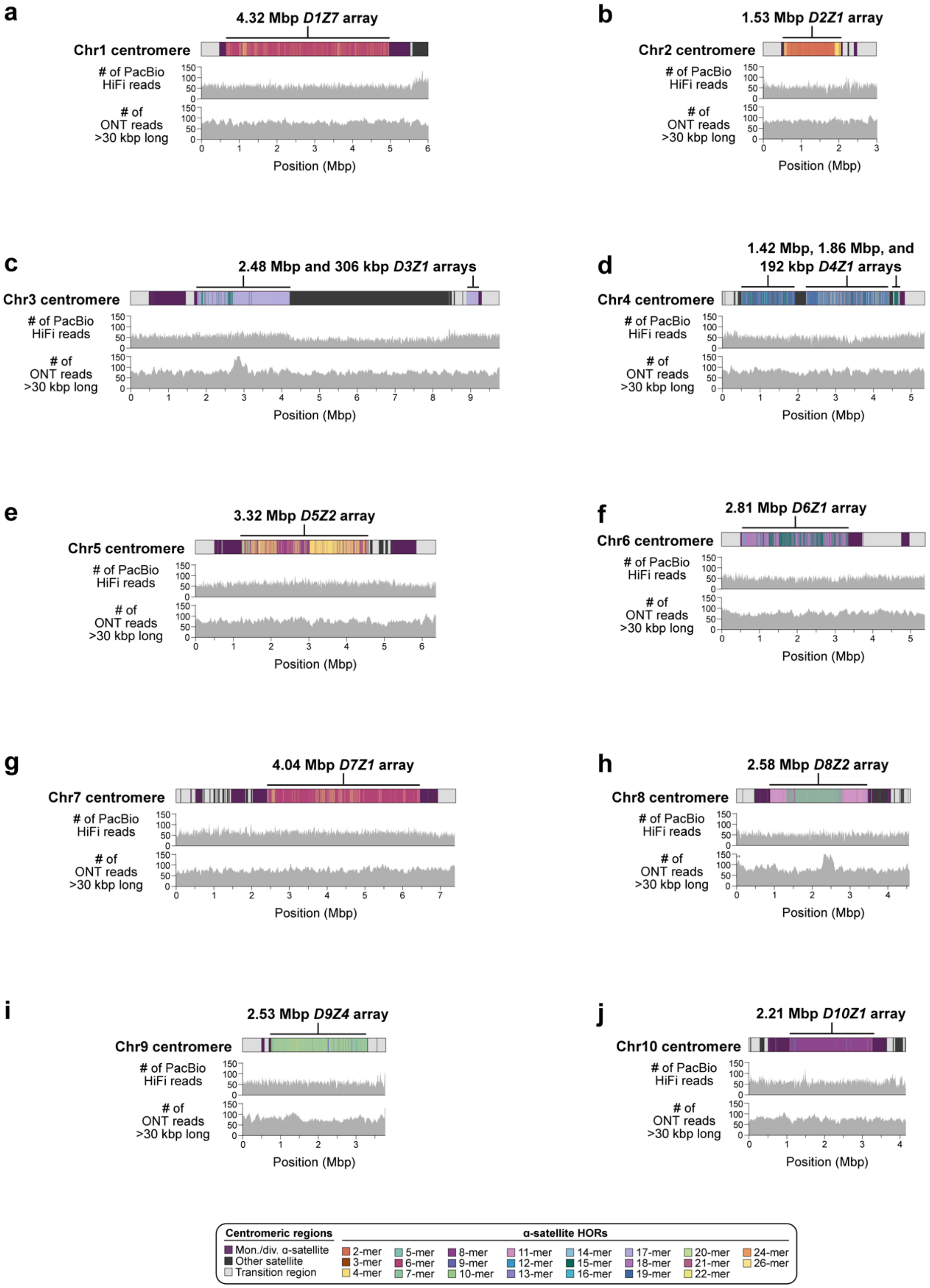


**Supplementary Figure 4. Read-depth profiles of the CHM1 chromosome 1-10 centromeres.** **a-j)** Alignment of CHM1 PacBio HiFi and ONT long-read sequencing data to the CHM1 centromere assemblies from chromosomes 1-10 shows uniform read depth, indicating a lack of large structural errors. Read-depth histograms of these regions are shown in **Supplementary Figs. 7,8**.

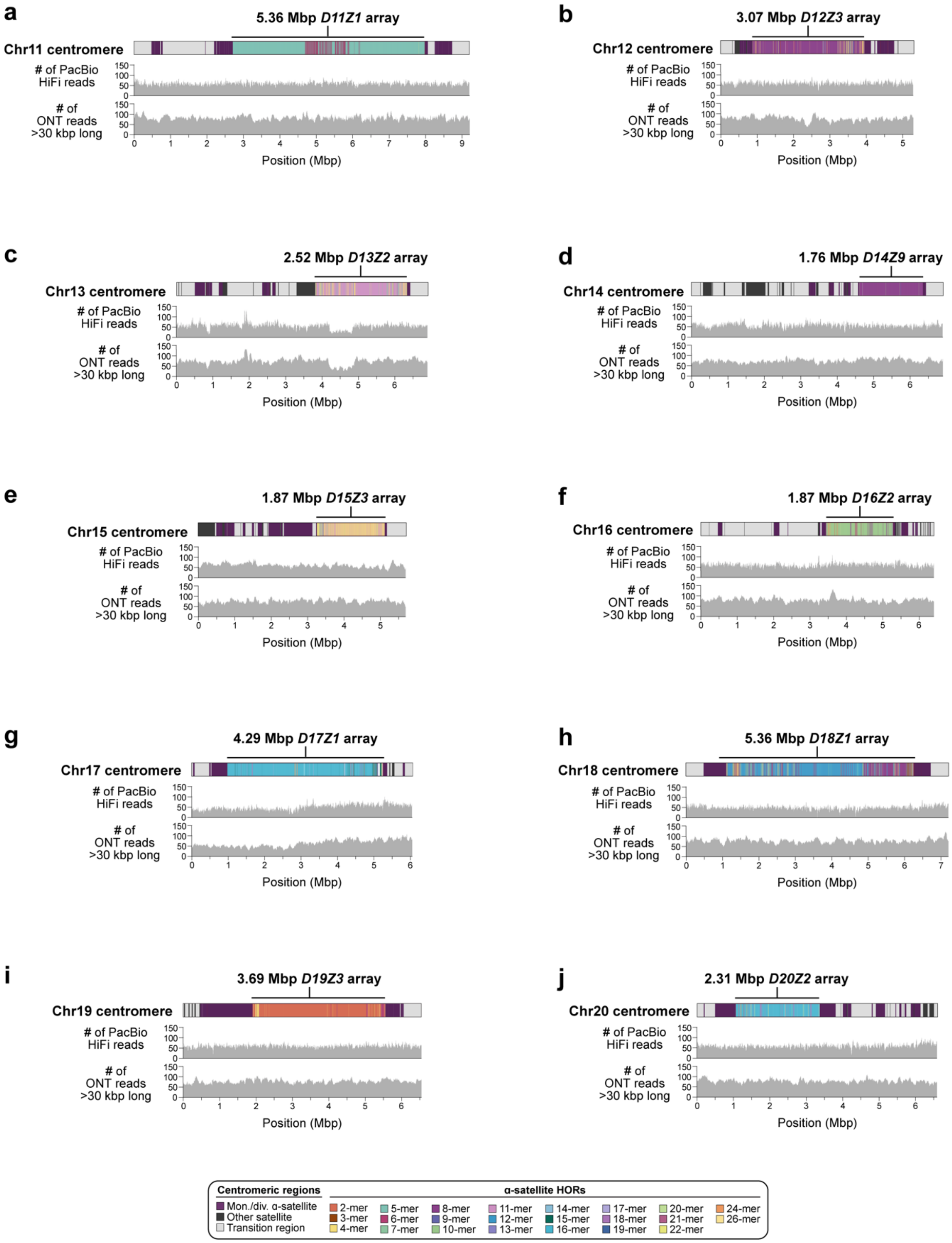


**Supplementary Figure 5. Read-depth profiles of the CHM1 chromosome 11-20 centromeres. a-j)** Alignment of CHM1 PacBio HiFi and ONT long-read sequencing data to the CHM1 centromere assemblies from chromosomes 11-20 shows uniform read depth, indicating a lack of large structural errors. Read-depth histograms of these regions are shown in **Supplementary Figs. 7,8**.

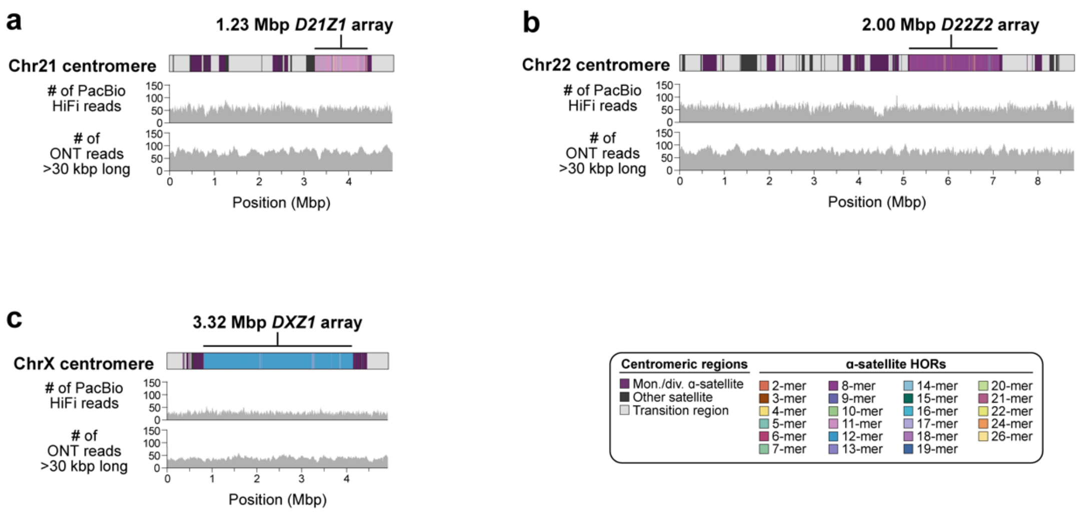


**Supplementary Figure 6. Read-depth profiles of CHM1 chromosome 21, 22, and X centromeres. a-c)** Alignment of CHM1 PacBio HiFi and ONT long-read sequencing data to the CHM1 centromere assemblies from chromosomes 21, 22, and X shows uniform read depth, indicating a lack of large structural errors. Read-depth histograms of these regions are shown in **Supplementary Fig. 8**.

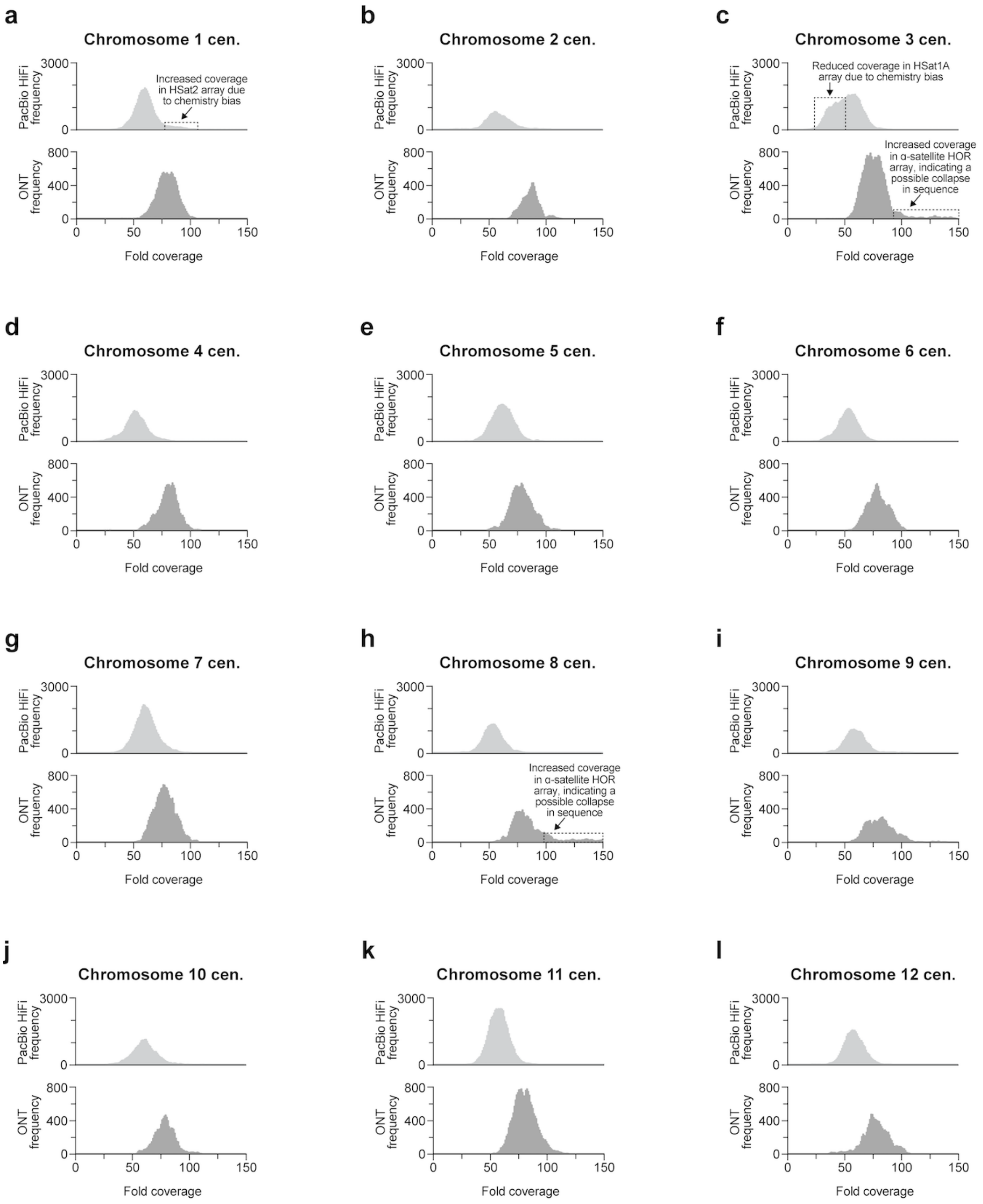


**Supplementary Figure 7. PacBio HiFi and ONT read-depth histograms for CHM1 chromosome 1-12 centromeres. a-l)** Histograms of the PacBio HiFi (top) and ONT (bottom) read depths across the CHM1 chromosome 1-12 centromeres. While most of these distributions are consistent with Poisson sampling, we identify three centromeres with increased or reduced coverage in PacBio HiFi and/or ONT data (chromosomes 1, 3, 8). Two of these are due to sequencing biases in PacBio chemistry[14], which results in increased coverage of HSat2 sequences (chromosome 1) or reduced coverage of HSat1A sequences (chromosome 3). However, we also identify increased coverage of ONT data on the centromeres from chromosomes 3 and 8, which may indicate a possible collapse in sequence in these centromeres that is detected with longer ONT reads but not with shorter PacBio HiFi reads. Importantly, neither of these regions are the site of hypomethylation or CENP-A chromatin enrichment and are not thought to contribute to kinetochore assembly.

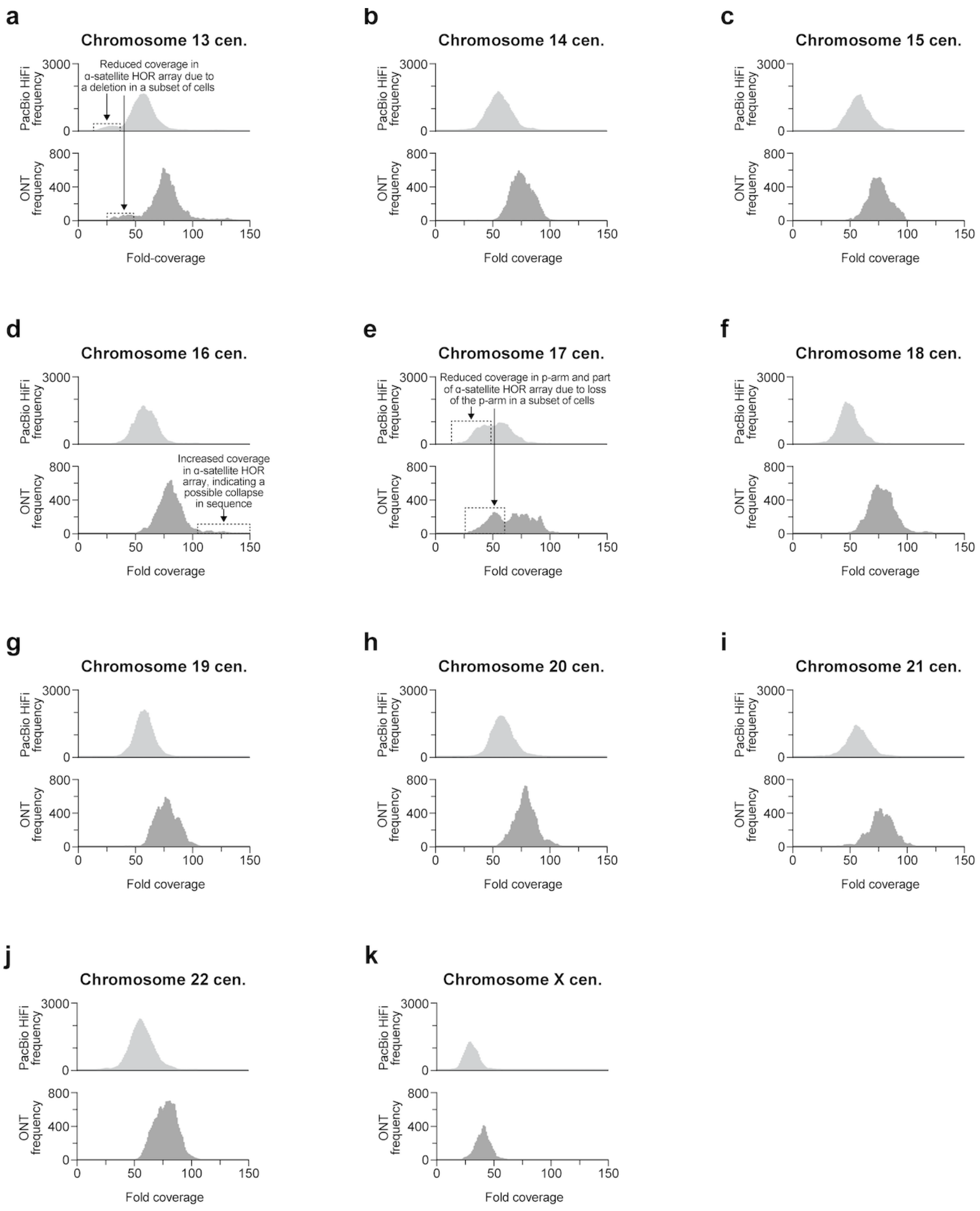


**Supplementary Figure 8. PacBio HiFi and ONT read-depth histograms for CHM1 chromosome 13-22 and X centromeres. a-k)** Histograms of the PacBio HiFi (top) and ONT (bottom) read depths across the CHM1 chromosome 13-22 and X centromeres. Most of these distributions are consistent with Poisson sampling. However, we identified three centromeres with increased or reduced coverage in PacBio HiFi and/or ONT data (chromosomes 13, 16, 17). Two of these (chromosomes 13 and 17) have reduced PacBio and ONT coverage due to a deletion in sequence in a subset of cells (**Supplementary Notes 1 and 2**). However, we also identify increased coverage of ONT data in the chromosome 16 centromere, which may indicate a possible collapse in sequence that is detected with longer ONT reads but not with shorter PacBio HiFi reads. Importantly, none of these regions are the site of hypomethylation or CENP-A chromatin enrichment and are not thought to contribute to kinetochore assembly.

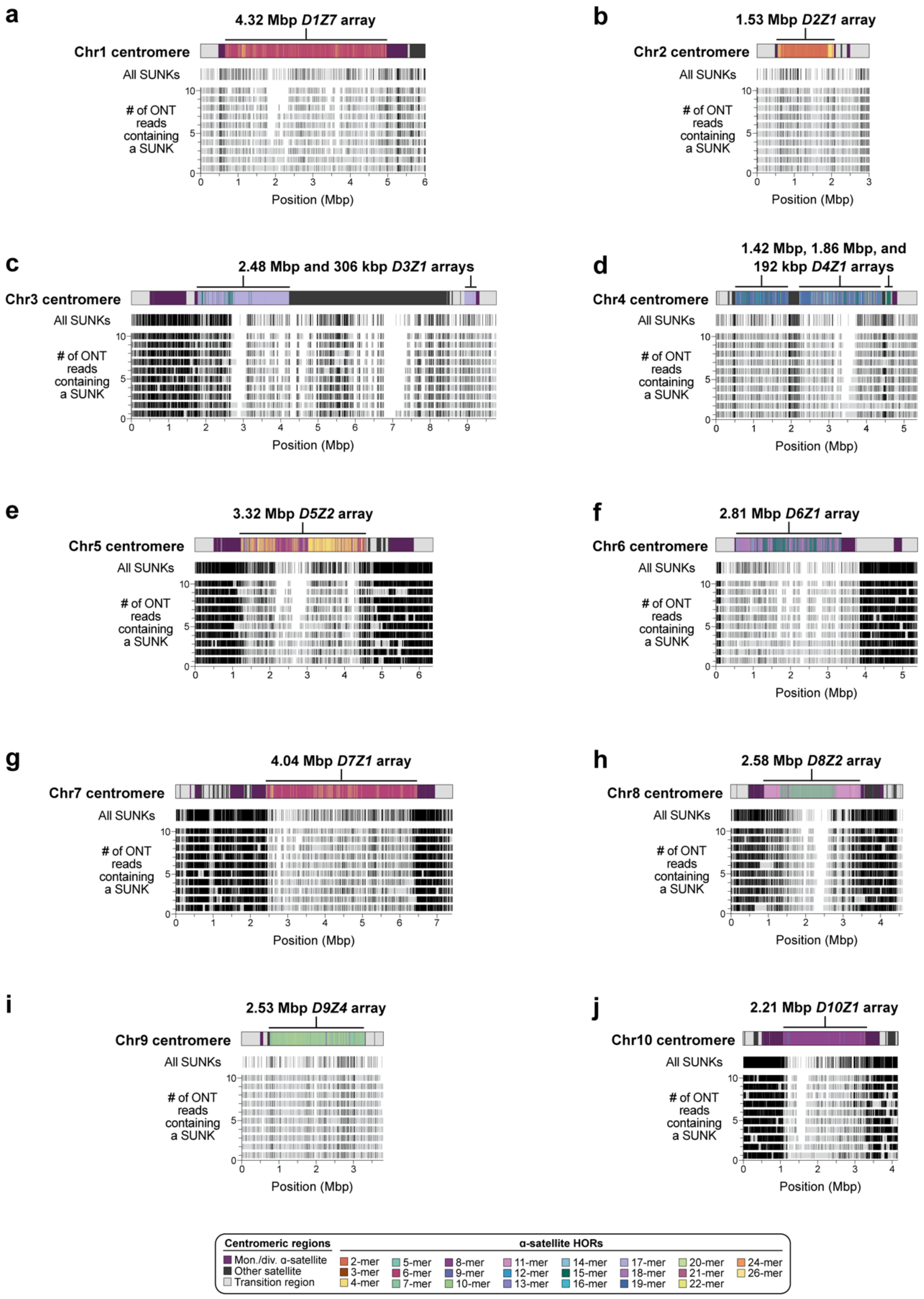


**Supplementary Figure 9. Validation of CHM1 chromosome 1-10 centromere assemblies with native ONT reads via GAVISUNK. a-j)** Plots showing the concordance between the CHM1 centromere assemblies and native ONT reads based on patterns of SUNKs (black vertical bars). Plots are generated with GAVISUNK[15].

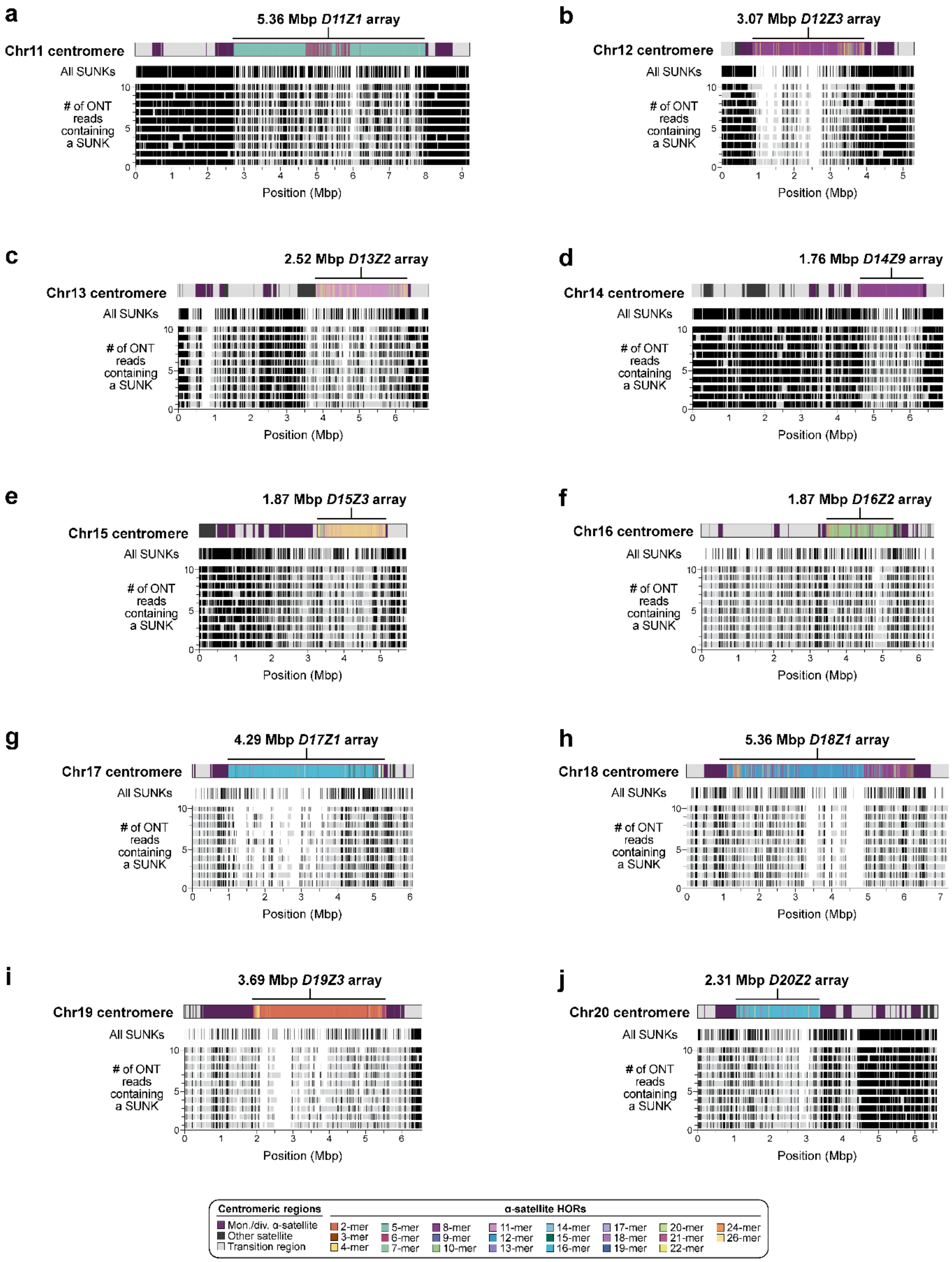


**Supplementary Figure 10. Validation of CHM1 chromosome 11-20 centromere assemblies with native ONT reads via GAVISUNK. a-j)** Plots showing the concordance between the CHM1 centromere assemblies and native ONT reads based on patterns of SUNKs (black vertical bars). Plots are generated with GAVISUNK[15].

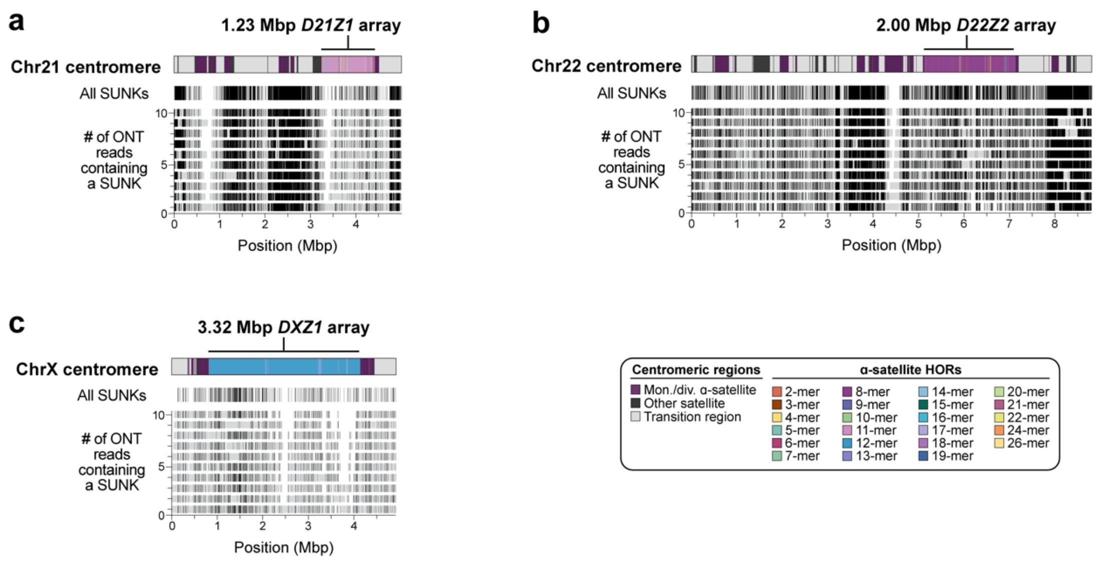


**Supplementary Figure 11. Validation of CHM1 chromosome 21, 22, and X centromere assemblies with native ONT reads via GAVISUNK. a-c)** Plots showing the concordance between the CHM1 centromere assemblies and native ONT reads based on patterns of SUNKs (black vertical bars). Plots are generated with GAVISUNK[15].

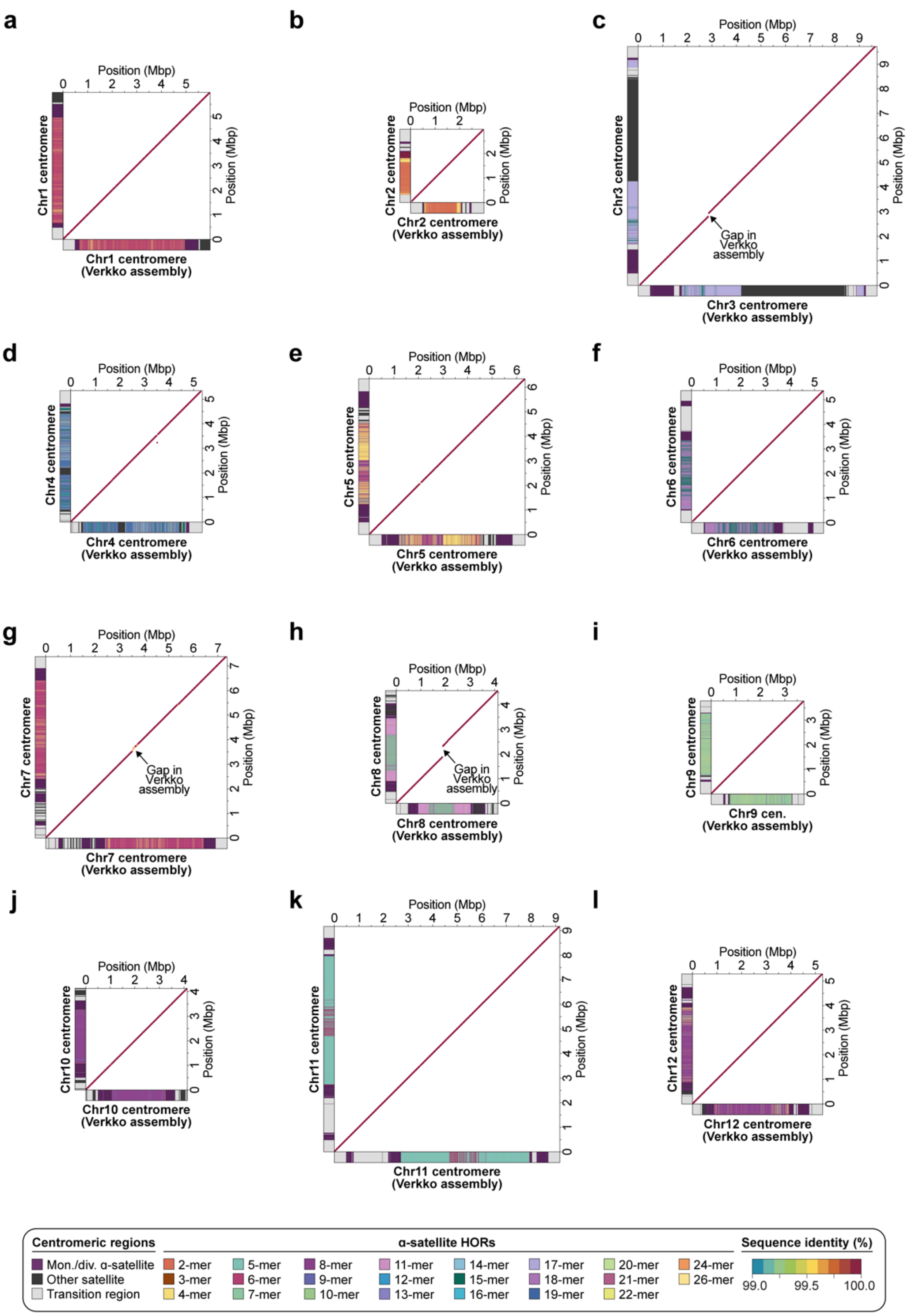


**Supplementary Figure 12. Comparison of CHM1 chromosome 1-12 centromere assemblies to those generated by another assembler, Verkko. a-l)** Plots showing the % sequence identity between the CHM1 centromere assemblies generated in this study and those generated via Verkko[16]. Each centromere is >99.9% identical in sequence. Gaps in the Verkko centromere assemblies are indicated. Plots were generated with StainedGlass[17].

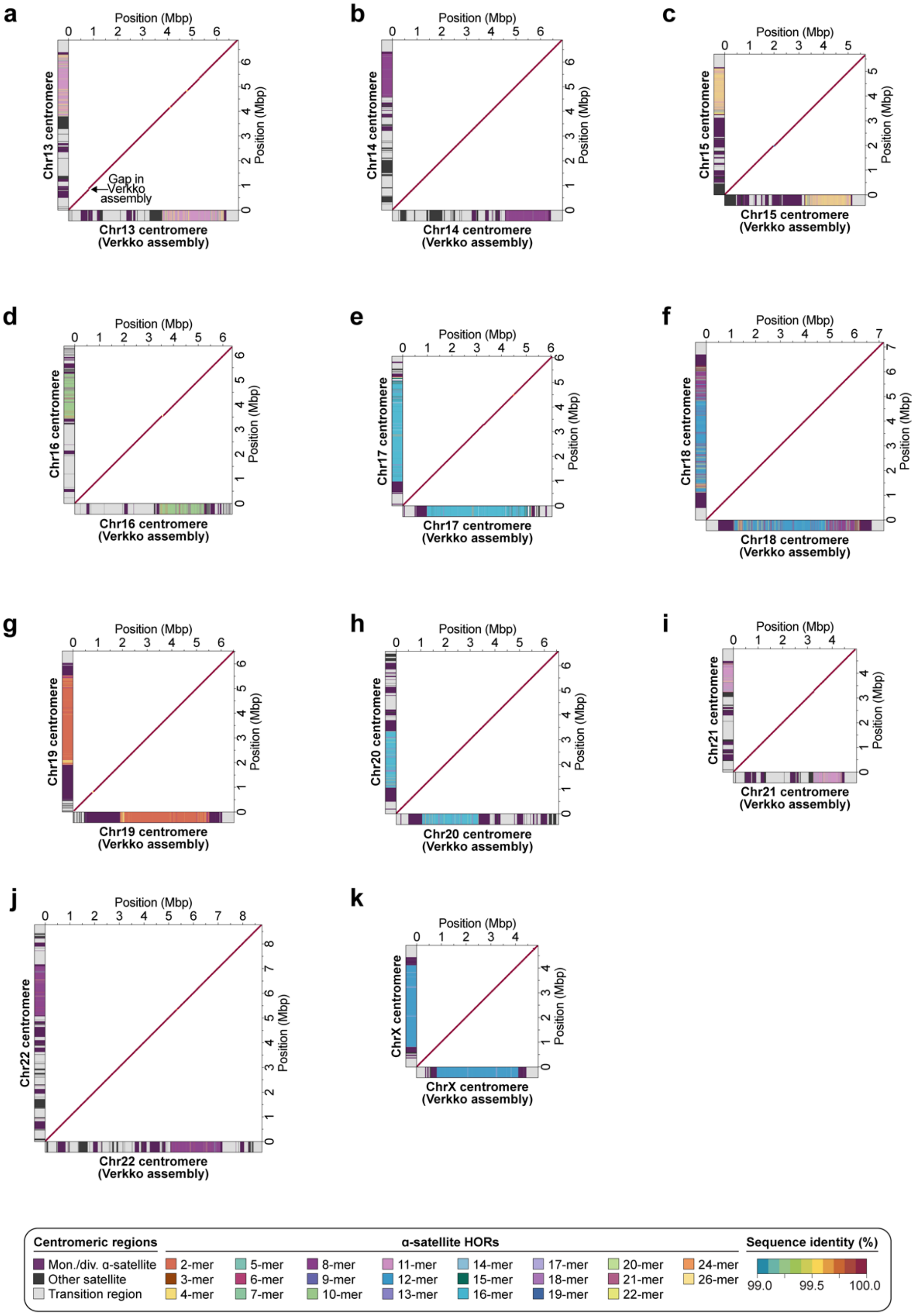


**Supplementary Figure 13. Comparison of CHM1 chromosome 13-22 and X centromere assemblies to those generated by another assembler, Verkko. a-k)** Plots showing the % sequence identity between the CHM1 centromere assemblies generated in this study and those generated via Verkko[16]. Each centromere is >99.9% identical in sequence. Gaps in the Verkko centromere assemblies are indicated. Plots were generated with StainedGlass[17].

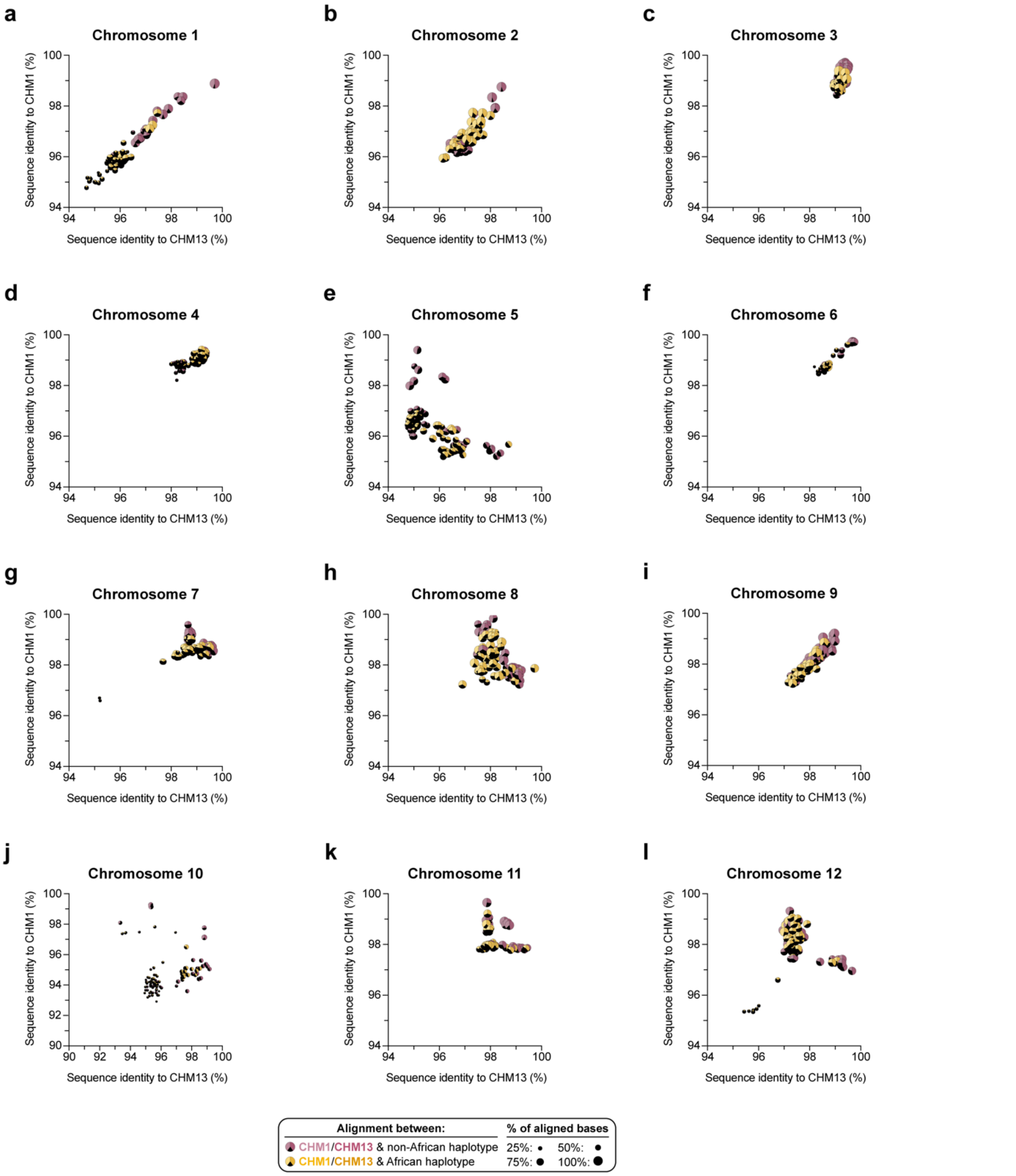


**Supplementary Figure 14. Variation in the sequence and structure of the chromosome 1-12 centromeric α-satellite higher-order repeat (HOR) arrays among 56 diverse human genomes. a-l)** Plots showing the percent sequence identity between centromeric α-satellite HOR arrays from CHM1 (y-axis), CHM13 (x-axis), and 56 other diverse human genomes (generated by the HPRC[18] and HGSVC[19]) for chromosomes 1-10. Each data point shows the percent of aligned bases from each human haplotype to either the CHM1 (left) or CHM13 (right) α-satellite HOR array(s). The percent of unaligned bases is shown in black. The size of each data point corresponds to the total percent of aligned bases among the CHM1 and CHM13 centromeric α-satellite HOR arrays. Precise quantification of the sequence identity and proportion of aligned versus unaligned sequences is provided in **Supplementary Table 6**.

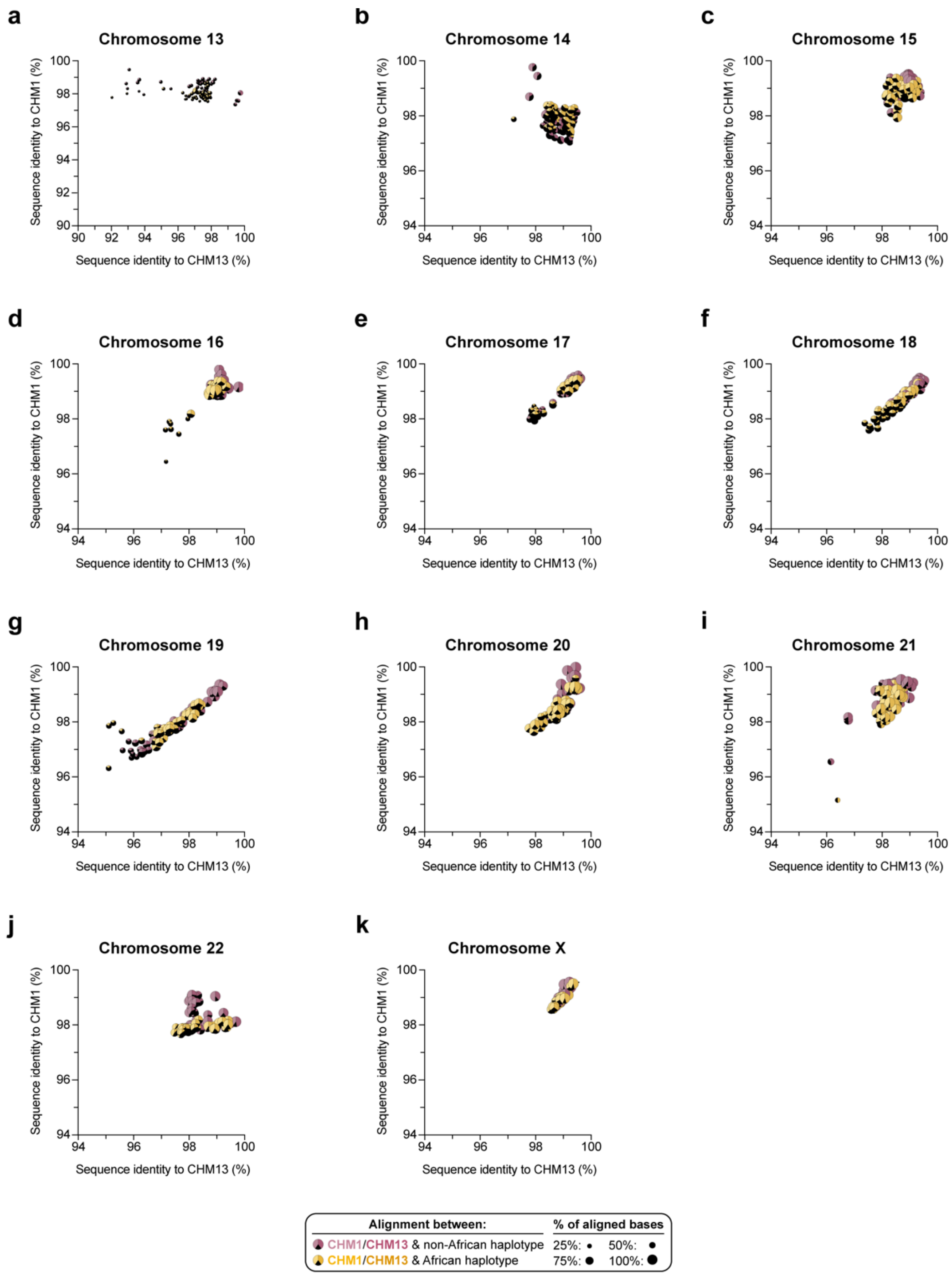


**Supplementary Figure 15. Variation in the sequence and structure of the chromosome 13-22 and X centromeric α-satellite HOR arrays among 56 diverse human genomes. a-k)** Plots showing the percent sequence identity between centromeric α-satellite HOR arrays from CHM1 (y-axis), CHM13 (x-axis), and 56 other diverse human genomes (generated by the HPRC[9] and HGSVC[10]). Each data point shows the proportion of aligned bases from each human haplotype to either the CHM1 (left) or CHM13 (right) α-satellite HOR array(s). The proportion of unaligned bases is shown in black. The size of each data point corresponds to the total proportion of aligned bases among the CHM1 and CHM13 centromeric α-satellite HOR arrays. Precise quantification of the sequence identity and proportions of aligned versus unaligned sequences is provided in **Supplementary Table 6**.

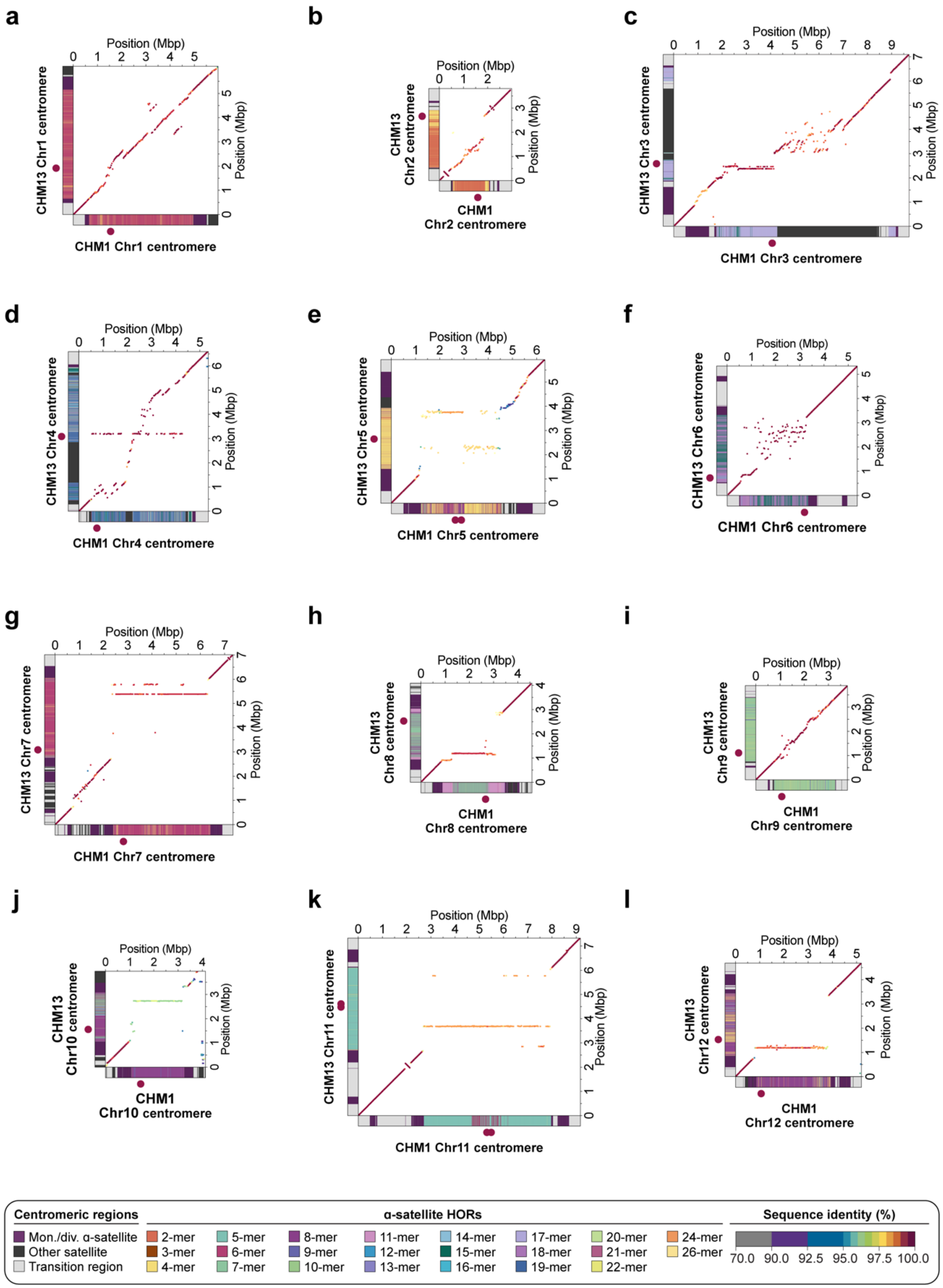


**Supplementary Figure 16. Comparison of the CHM1 and CHM13 chromosome 1-12 centromeric regions. a-l)** Dot plots showing the percent sequence identity between the CHM1 and CHM13 centromeric regions for chromosomes 1-12. Plots were generated with StainedGlass[17].

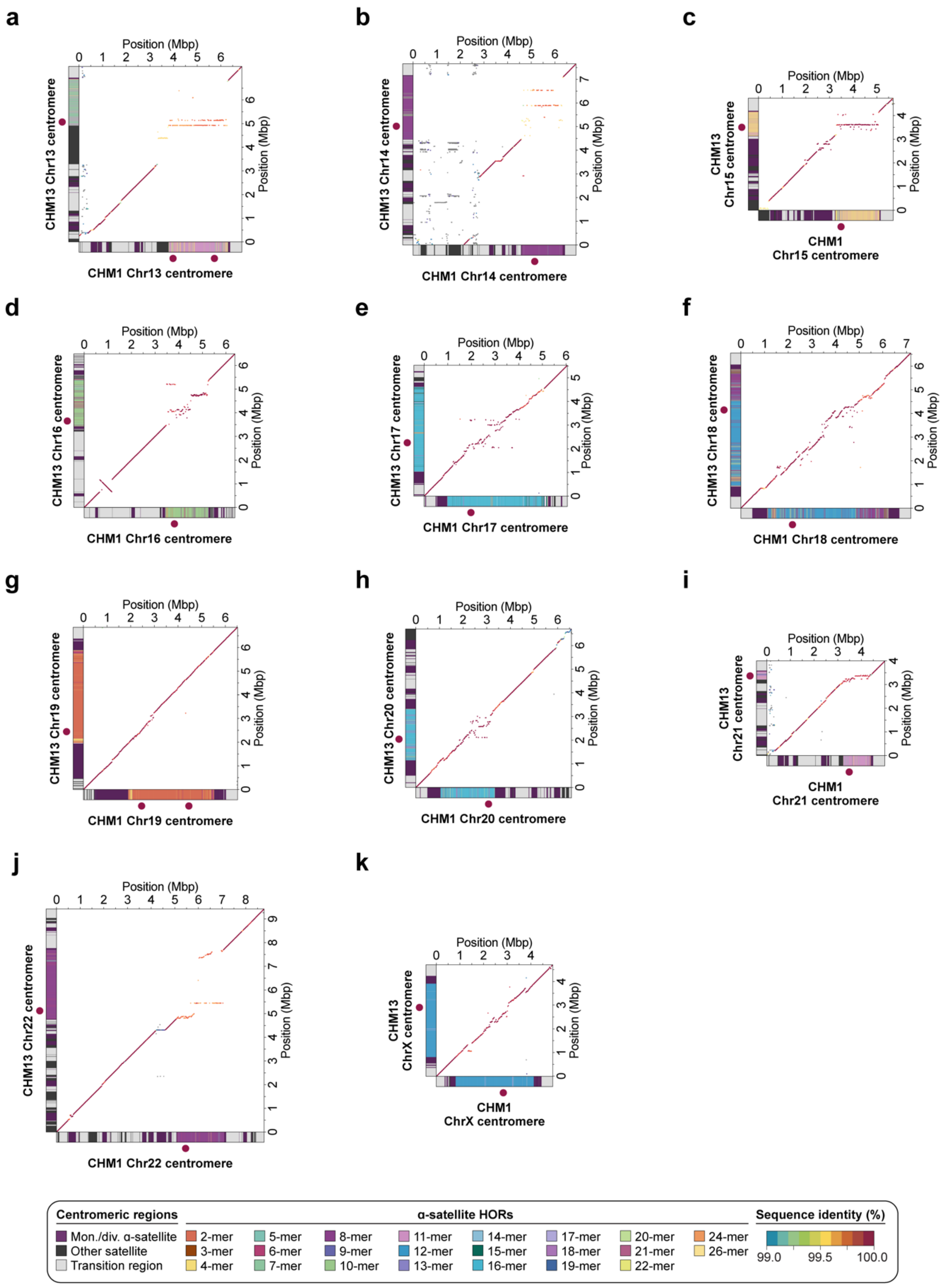


**Supplementary Figure 17. Comparison of the CHM1 and CHM13 chromosome 13-22 and X centromeric regions. a-k)** Dot plots showing the percent sequence identity between the CHM1 and CHM13 centromeric regions for chromosomes 13-22 and X. Plots were generated with StainedGlass[17].

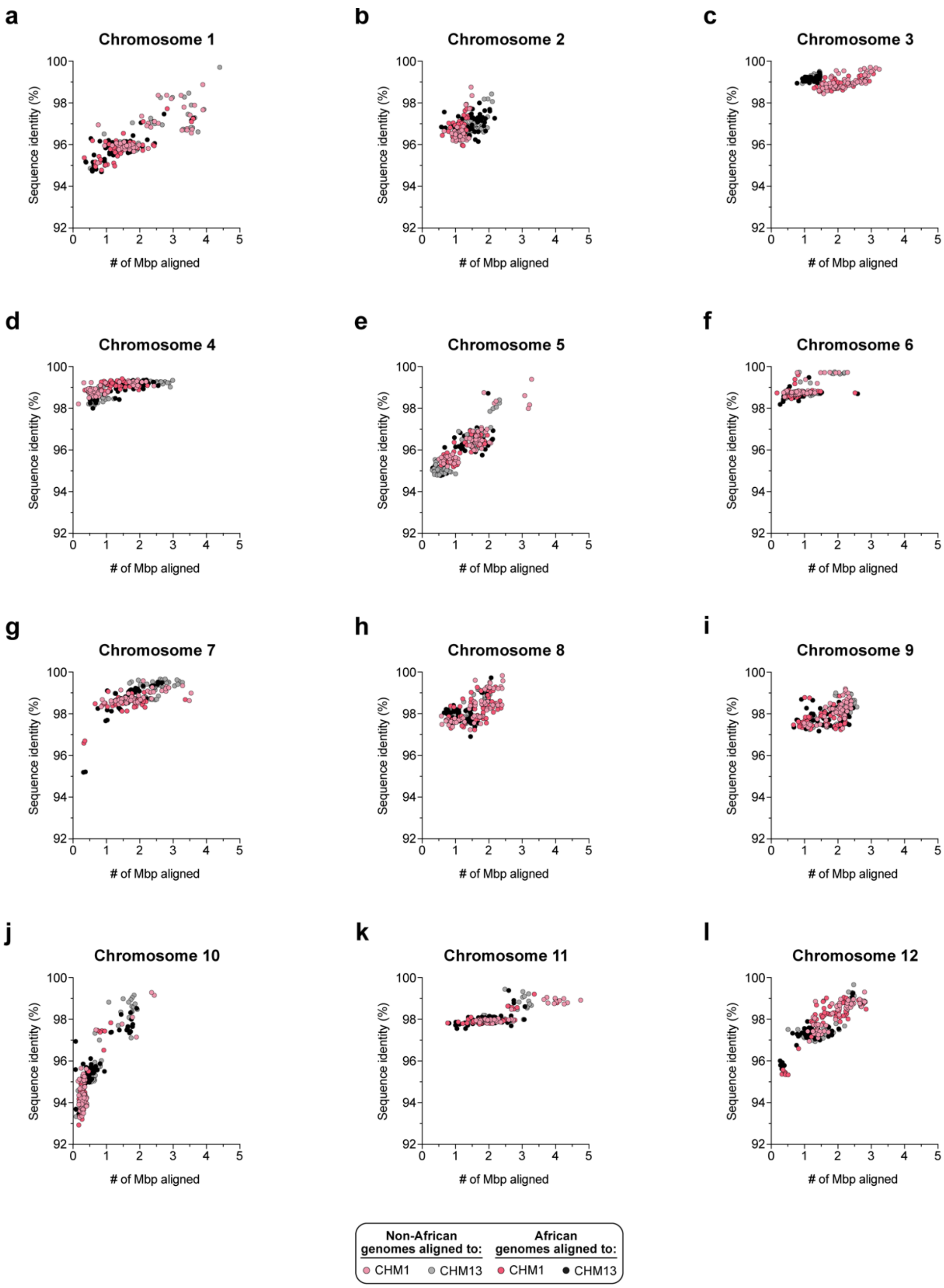


**Supplementary Figure 18. Comparison of the CHM1 and CHM13 chromosome 1-12 centromeric α-satellite HOR arrays to those from 56 diverse human genomes. a-l)** Plots showing the percent sequence identity and number of megabase pairs (Mbp) aligned for 56 diverse human genomes (112 haplotypes), generated by the HPRC[18] and HGSVC[19], mapped to the CHM1 and CHM13 chromosome 1-12 centromeric regions. Note that each data point represents a haplotype with 1:1 best mapping, although many of the centromeres are not yet complete in the HPRC and HGSVC assemblies.

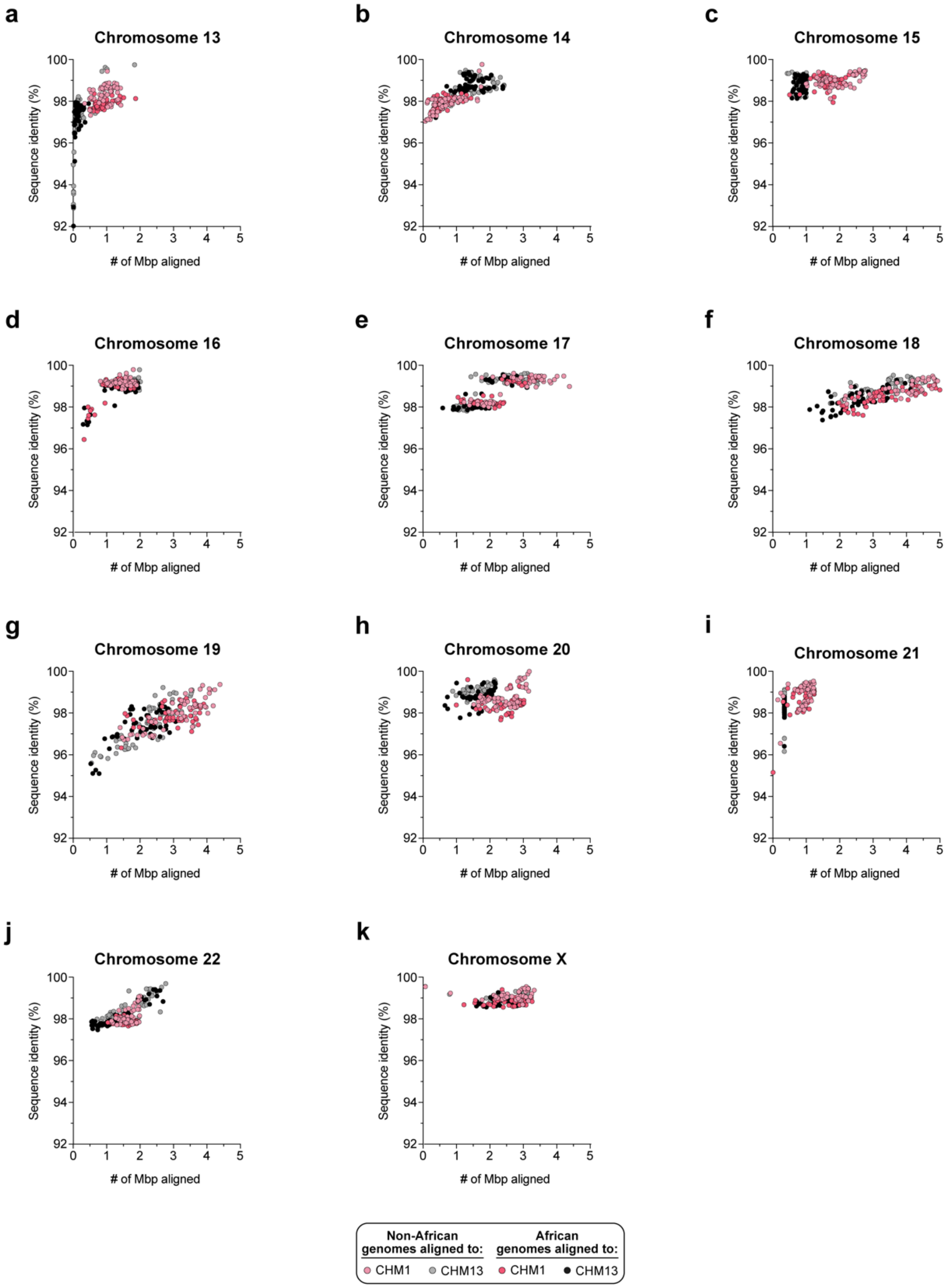


**Supplementary Figure 19. Comparison of the CHM1 and CHM13 chromosome 13-22 and X centromeric α-satellite HOR arrays to those from 56 diverse human genomes. a-k)** Plots showing the percent sequence identity and number of megabase pairs (Mbp) aligned for 56 diverse human genomes (112 haplotypes), generated by the HPRC[18] and HGSVC[19], mapped to the CHM1 and CHM13 chromosome 13-22 and X centromeric regions. Note that each data point represents a haplotype with 1:1 best mapping, although many of the centromeres are not yet complete in the HPRC and HGSVC assemblies.

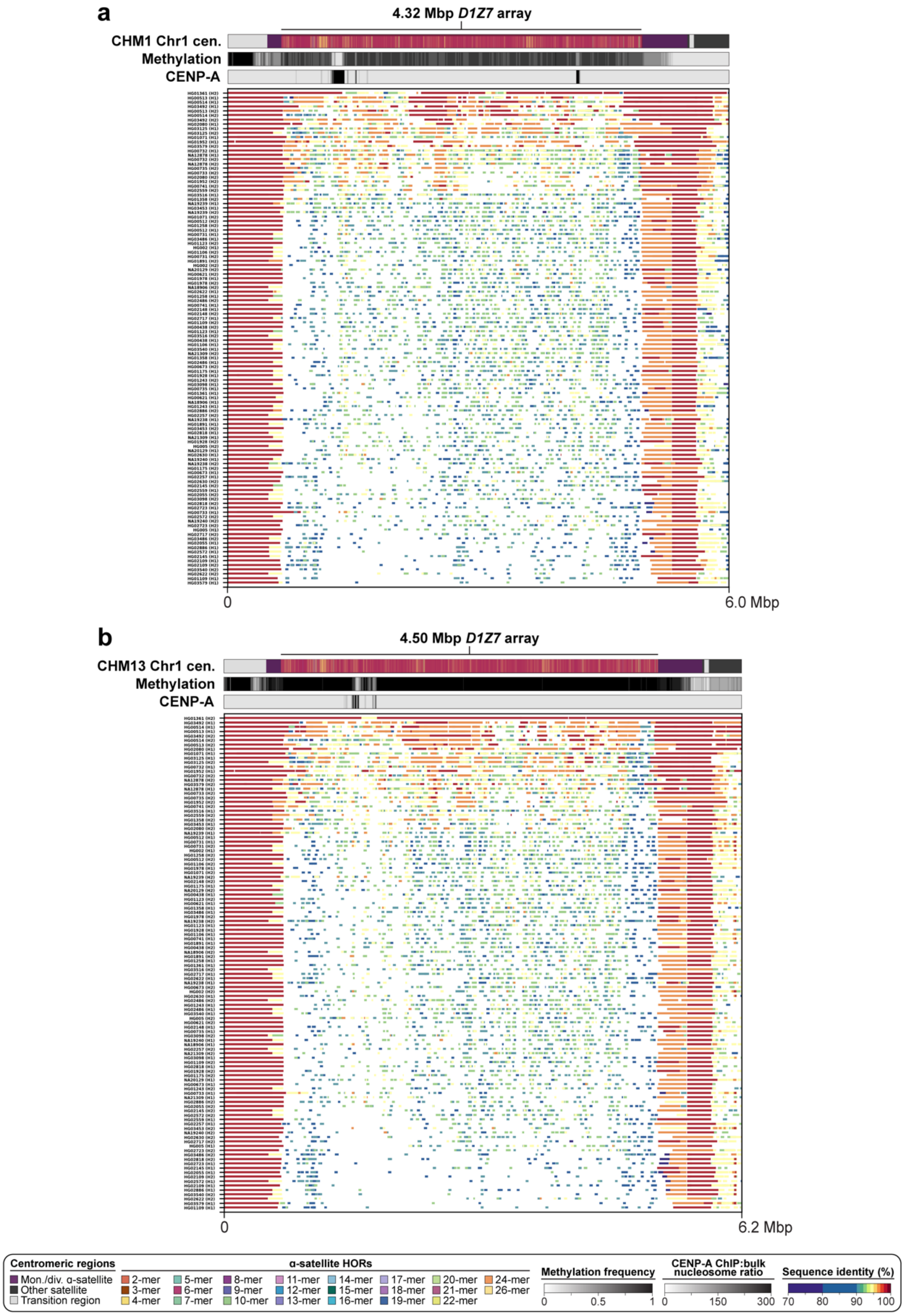


**Supplementary Figure 20. Comparison of the sequence and structure of the chromosome 1 centromeric region from the CHM1, CHM13, and 56 diverse human genomes. a,b)** Plots showing the sequence organization (top track), CpG methylation frequency (second track), CENP-A nucleosome enrichment (third track), and percent sequence identity of contigs from 56 diverse human genomes (112 haplotypes[18,19]) relative to the **a)** CHM1 and **b)** CHM13 genomes.

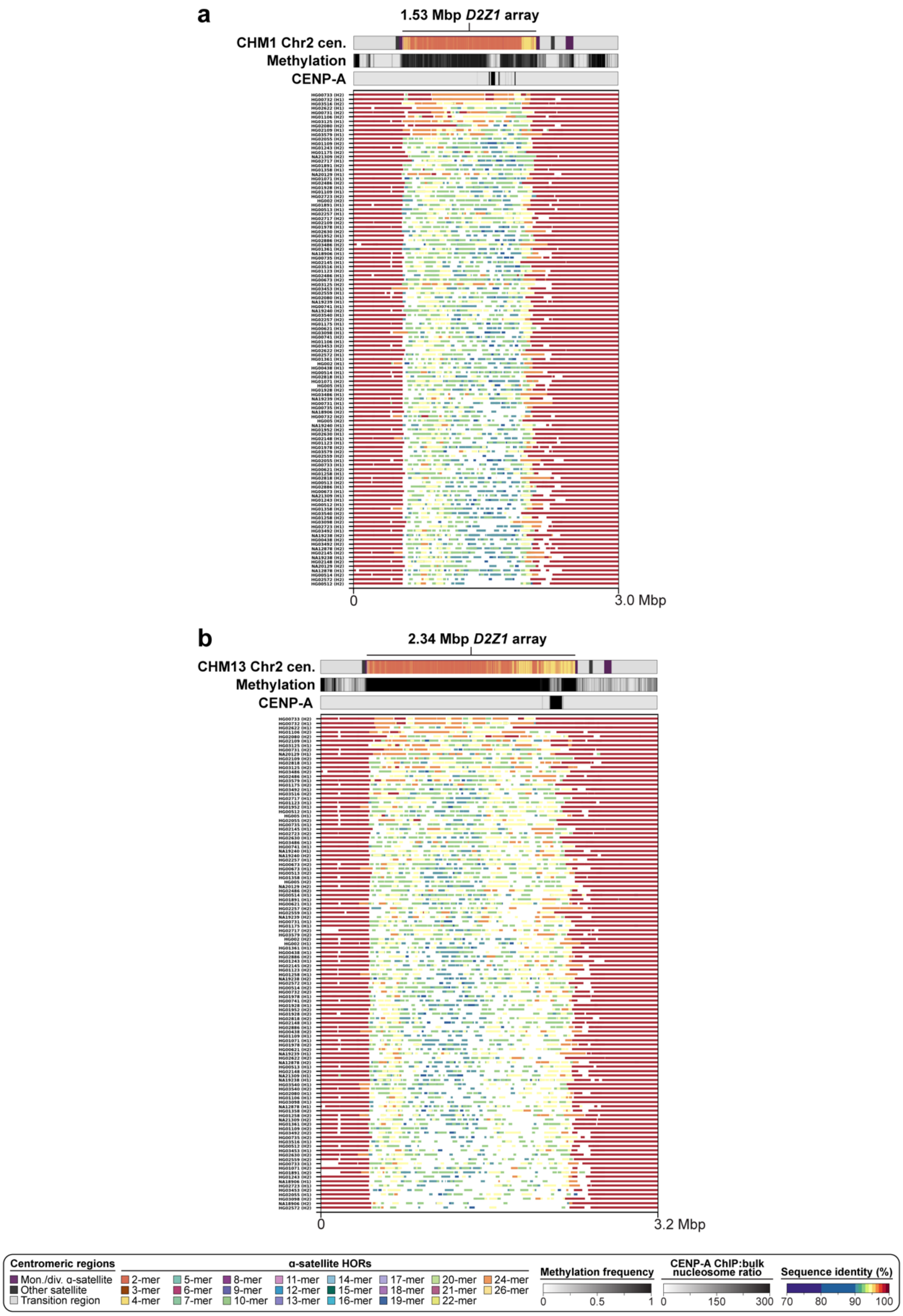


**Supplementary Figure 21. Comparison of the sequence and structure of the chromosome 2 centromeric region from the CHM1, CHM13, and 56 diverse human genomes. a,b)** Plots showing the sequence organization (top track), CpG methylation frequency (second track), CENP-A nucleosome enrichment (third track), and percent sequence identity of contigs from 56 diverse human genomes (112 haplotypes[18,19]) relative to the **a)** CHM1 and **b)** CHM13 genomes.

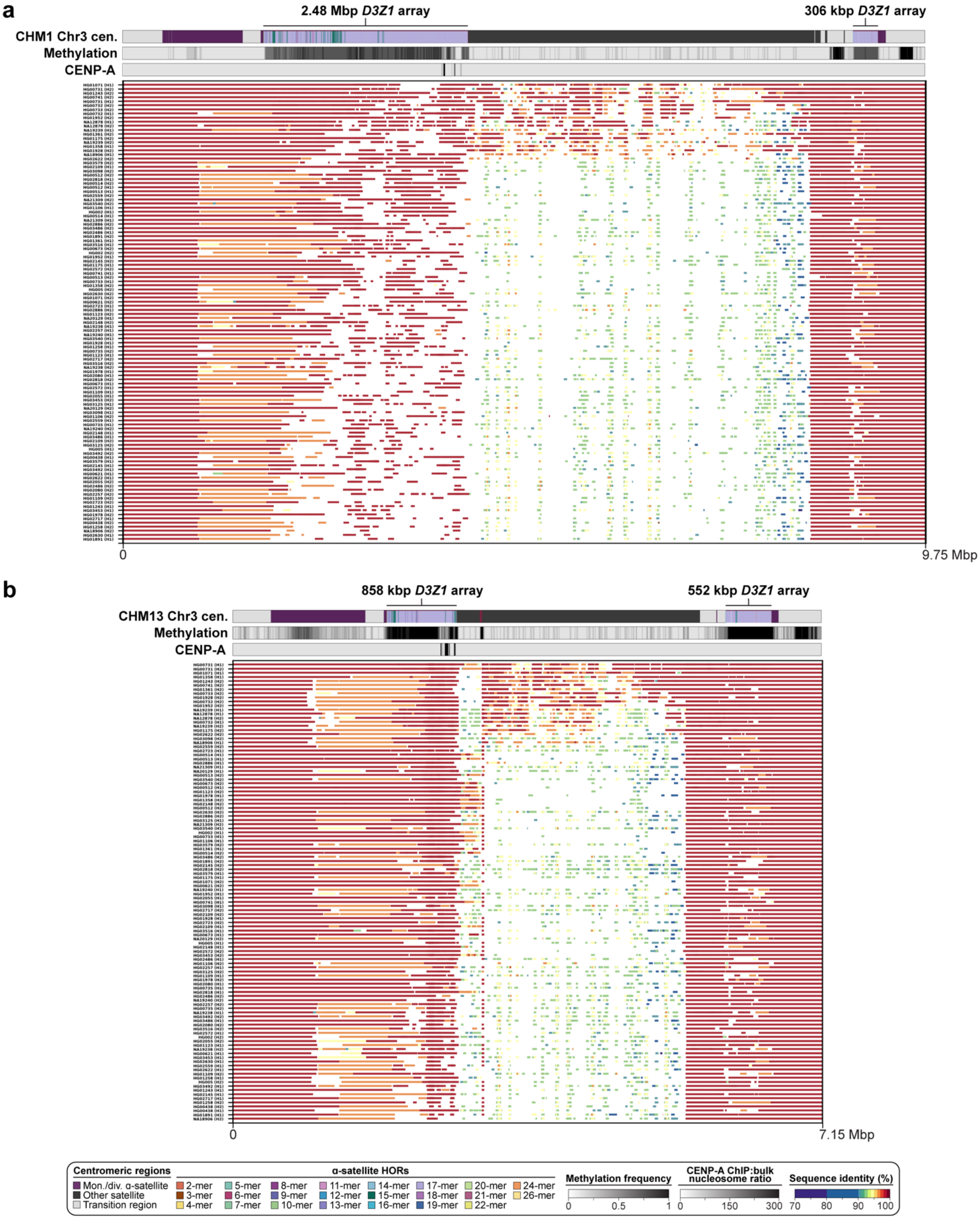


**Supplementary Figure 22. Comparison of the sequence and structure of the chromosome 3 centromeric region from the CHM1, CHM13, and 56 diverse human genomes. a,b)** Plots showing the sequence organization (top track), CpG methylation frequency (second track), CENP-A nucleosome enrichment (third track), and percent sequence identity of contigs from 56 diverse human genomes (112 haplotypes[18,19]) relative to the **a)** CHM1 and **b)** CHM13 genomes.

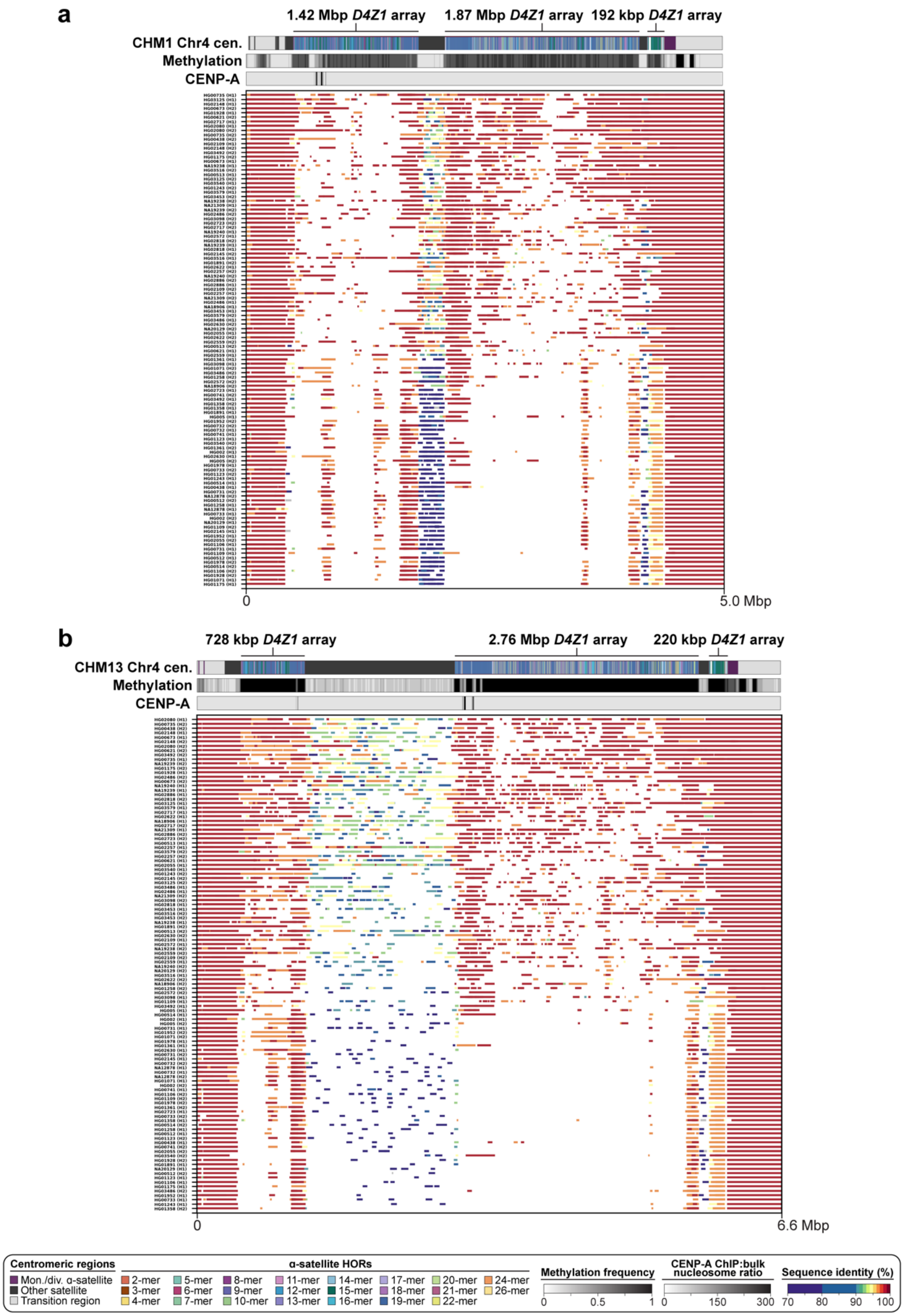


**Supplementary Figure 23. Comparison of the sequence and structure of the chromosome 4 centromeric region from the CHM1, CHM13, and 56 diverse human genomes. a,b)** Plots showing the sequence organization (top track), CpG methylation frequency (second track), CENP-A nucleosome enrichment (third track), and percent sequence identity of contigs from 56 diverse human genomes (112 haplotypes[18,19]) relative to the **a)** CHM1 and **b)** CHM13 genomes.

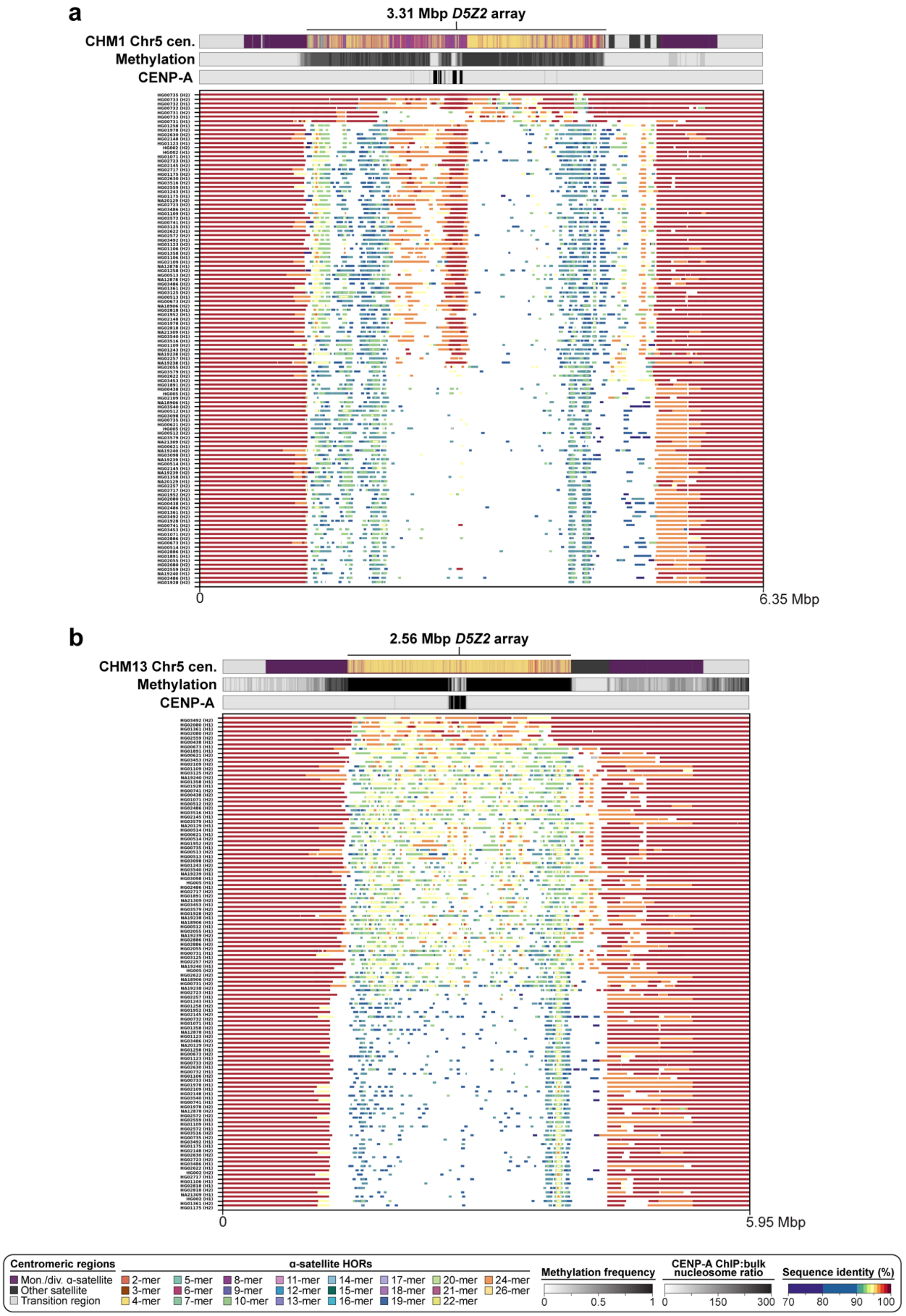


**Supplementary Figure 24. Comparison of the sequence and structure of the chromosome 5 centromeric region from the CHM1, CHM13, and 56 diverse human genomes. a,b)** Plots showing the sequence organization (top track), CpG methylation frequency (second track), CENP-A nucleosome enrichment (third track), and percent sequence identity of contigs from 56 diverse human genomes (112 haplotypes[18,19]) relative to the **a)** CHM1 and **b)** CHM13 genomes.

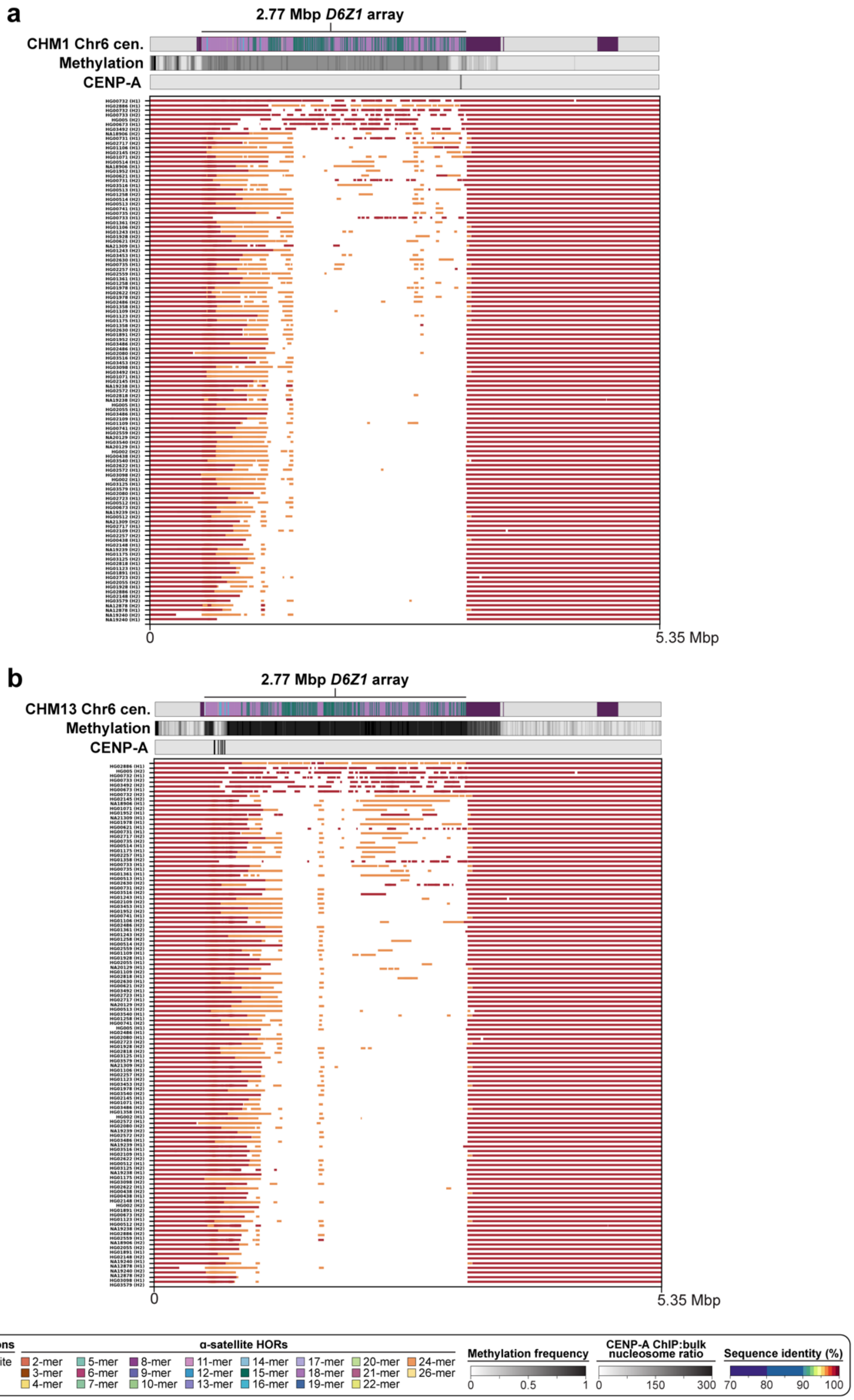


**Supplementary Figure 25. Comparison of the sequence and structure of the chromosome 6 centromeric region from the CHM1, CHM13, and 56 diverse human genomes. a,b)** Plots showing the sequence organization (top track), CpG methylation frequency (second track), CENP-A nucleosome enrichment (third track), and percent sequence identity of contigs from 56 diverse human genomes (112 haplotypes[18,19]) relative to the **a)** CHM1 and **b)** CHM13 genomes.

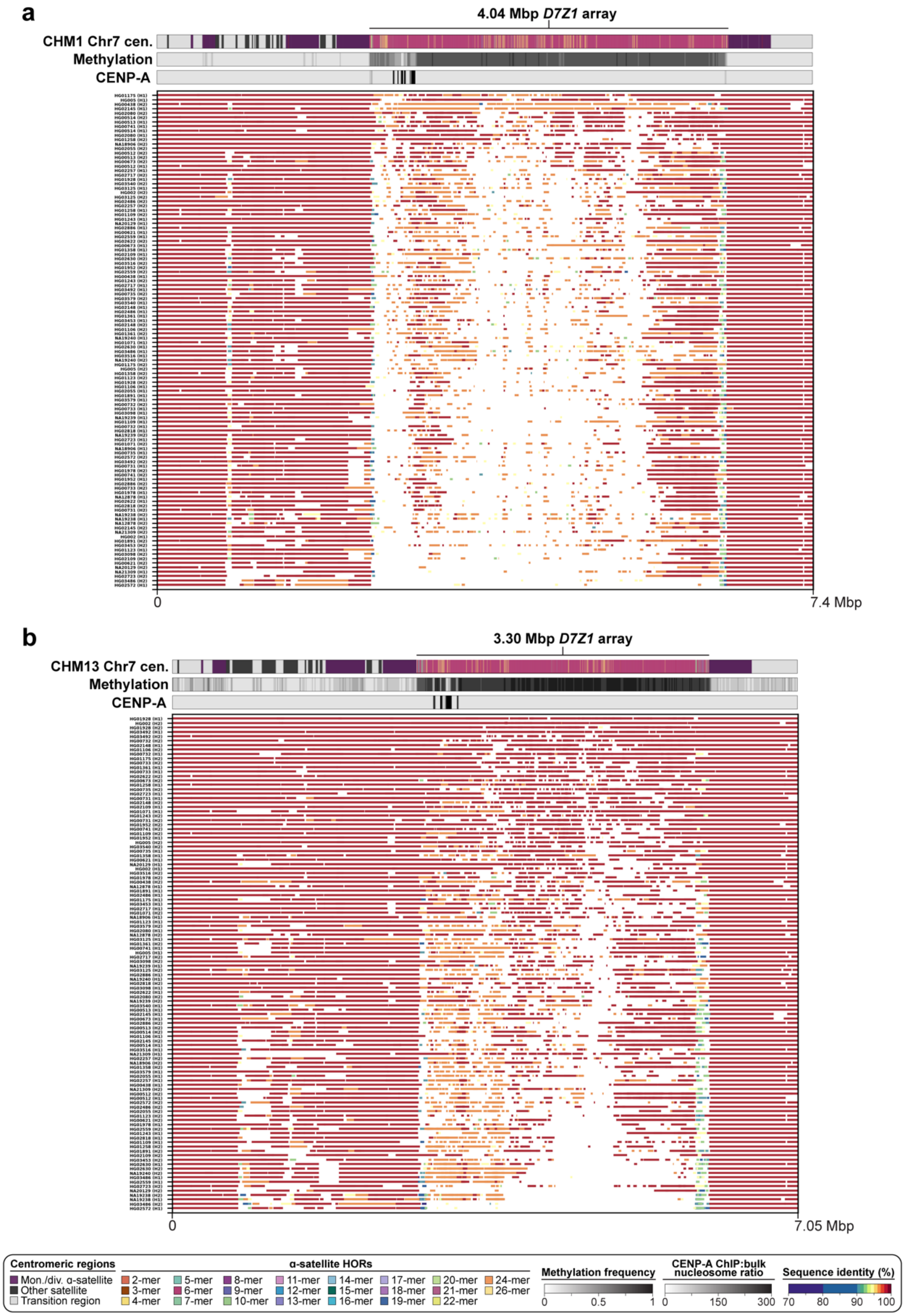


**Supplementary Figure 26. Comparison of the sequence and structure of the chromosome 7 centromeric region from the CHM1, CHM13, and 56 diverse human genomes. a,b)** Plots showing the sequence organization (top track), CpG methylation frequency (second track), CENP-A nucleosome enrichment (third track), and percent sequence identity of contigs from 56 diverse human genomes (112 haplotypes[18,19]) relative to the **a)** CHM1 and **b)** CHM13 genomes.

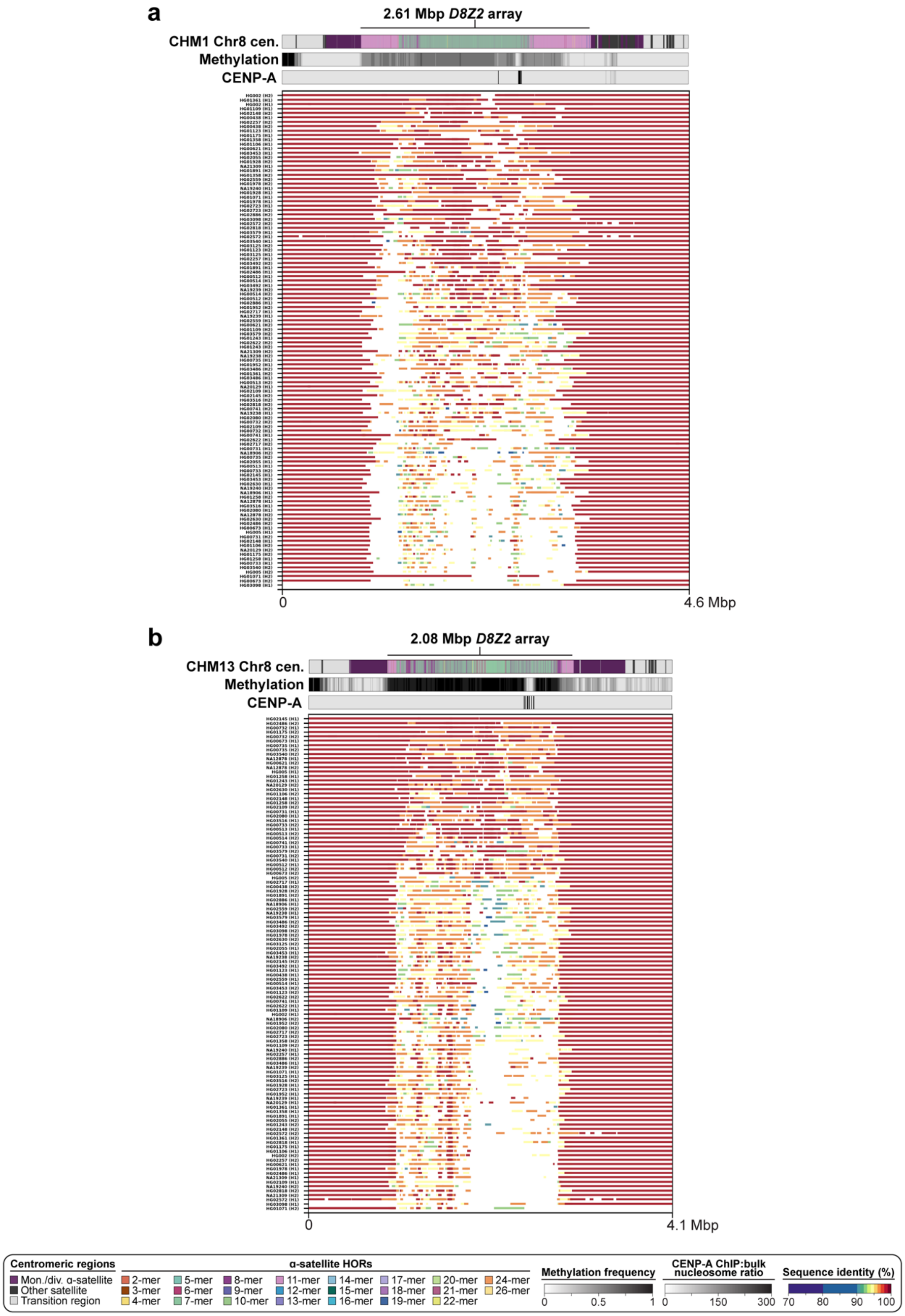


**Supplementary Figure 27. Comparison of the sequence and structure of the chromosome 8 centromeric region from the CHM1, CHM13, and 56 diverse human genomes. a,b)** Plots showing the sequence organization (top track), CpG methylation frequency (second track), CENP-A nucleosome enrichment (third track), and percent sequence identity of contigs from 56 diverse human genomes (112 haplotypes[18,19]) relative to the **a)** CHM1 and **b)** CHM13 genomes.

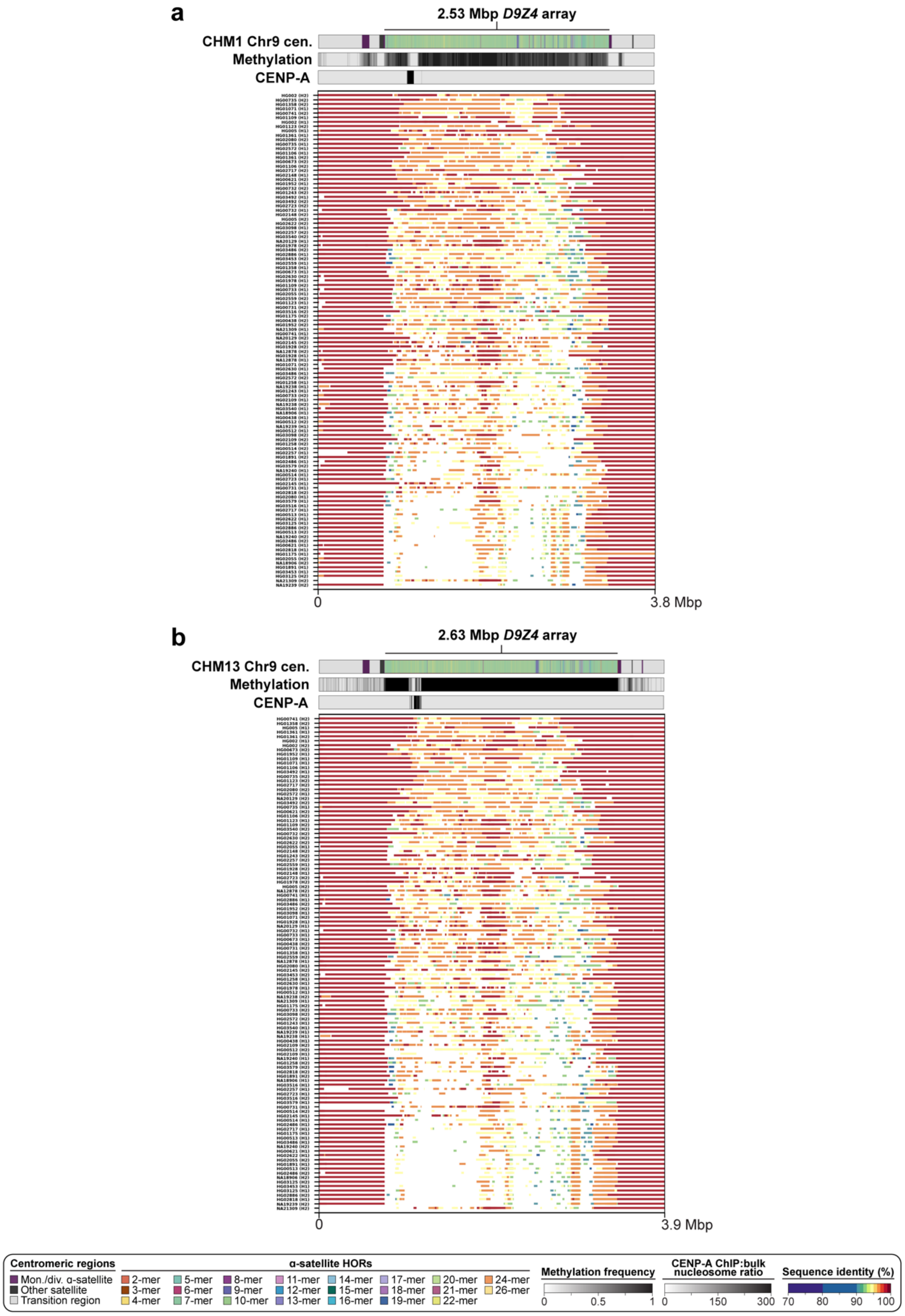


**Supplementary Figure 28. Comparison of the sequence and structure of the chromosome 9 centromeric region from the CHM1, CHM13, and 56 diverse human genomes. a,b)** Plots showing the sequence organization (top track), CpG methylation frequency (second track), CENP-A nucleosome enrichment (third track), and percent sequence identity of contigs from 56 diverse human genomes (112 haplotypes[18,19]) relative to the **a)** CHM1 and **b)** CHM13 genomes.

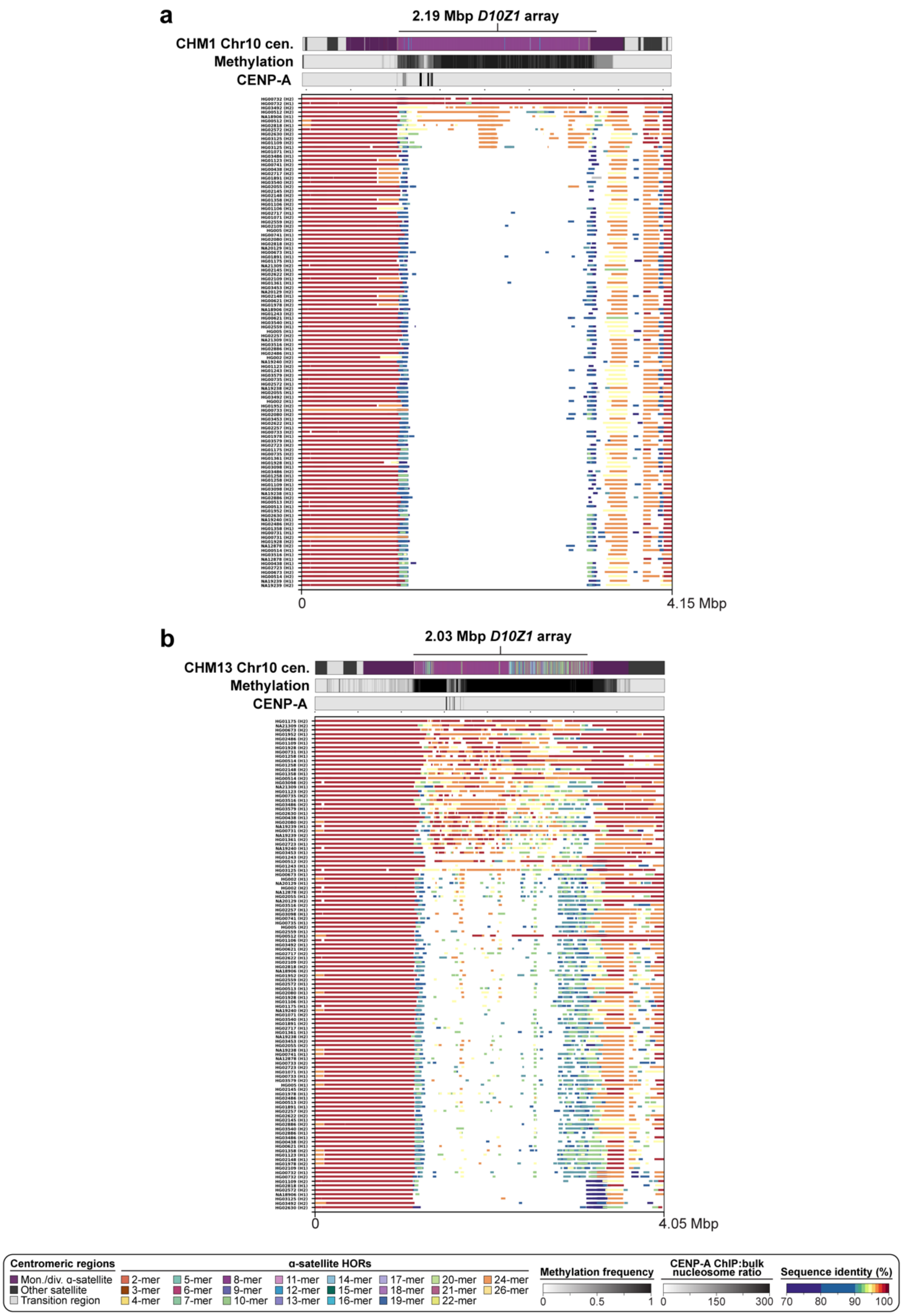


**Supplementary Figure 29. Comparison of the sequence and structure of the chromosome 10 centromeric region from the CHM1, CHM13, and 56 diverse human genomes. a,b)** Plots showing the sequence organization (top track), CpG methylation frequency (second track), CENP-A nucleosome enrichment (third track), and percent sequence identity of contigs from 56 diverse human genomes (112 haplotypes[18,19]) relative to the **a)** CHM1 and **b)** CHM13 genomes.

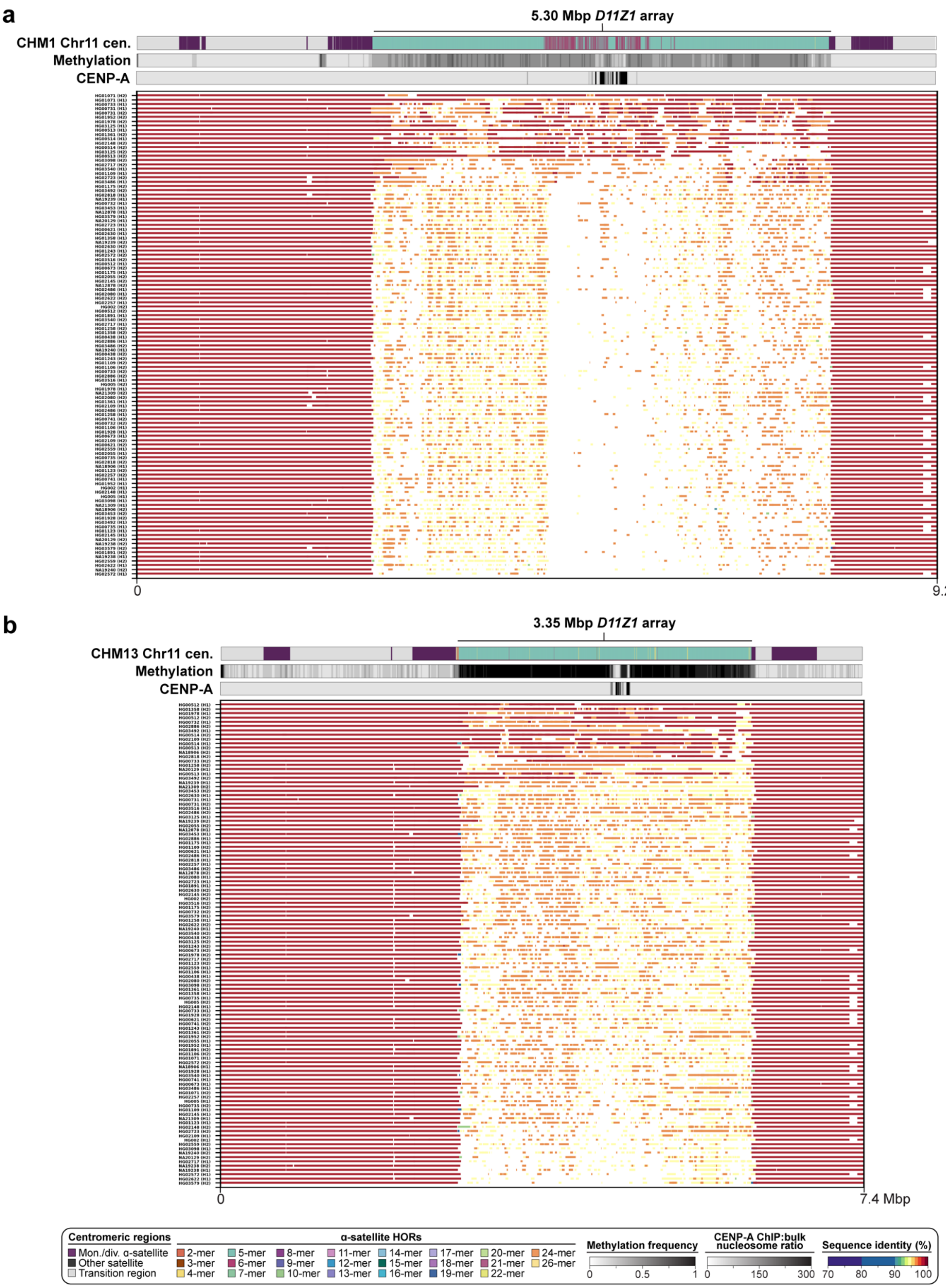


**Supplementary Figure 30. Comparison of the sequence and structure of the chromosome 11 centromeric region from the CHM1, CHM13, and 56 diverse human genomes. a,b)** Plots showing the sequence organization (top track), CpG methylation frequency (second track), CENP-A nucleosome enrichment (third track), and percent sequence identity of contigs from 56 diverse human genomes (112 haplotypes[18,19]) relative to the **a)** CHM1 and **b)** CHM13 genomes.

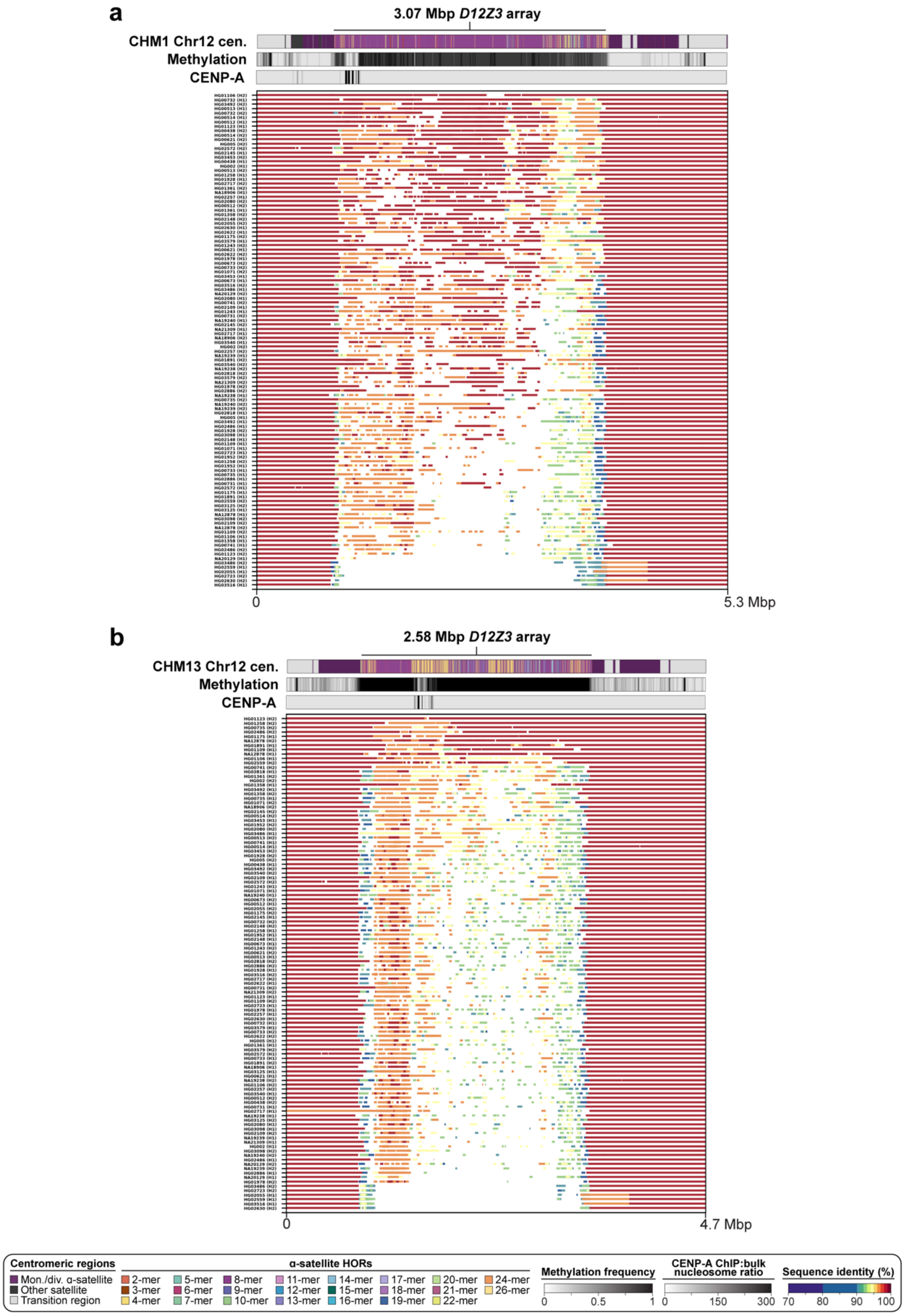


**Supplementary Figure 31. Comparison of the sequence and structure of the chromosome 12 centromeric region from the CHM1, CHM13, and 56 diverse human genomes. a,b)** Plots showing the sequence organization (top track), CpG methylation frequency (second track), CENP-A nucleosome enrichment (third track), and percent sequence identity of contigs from 56 diverse human genomes (112 haplotypes[18,19]) relative to the **a)** CHM1 and **b)** CHM13 genomes.

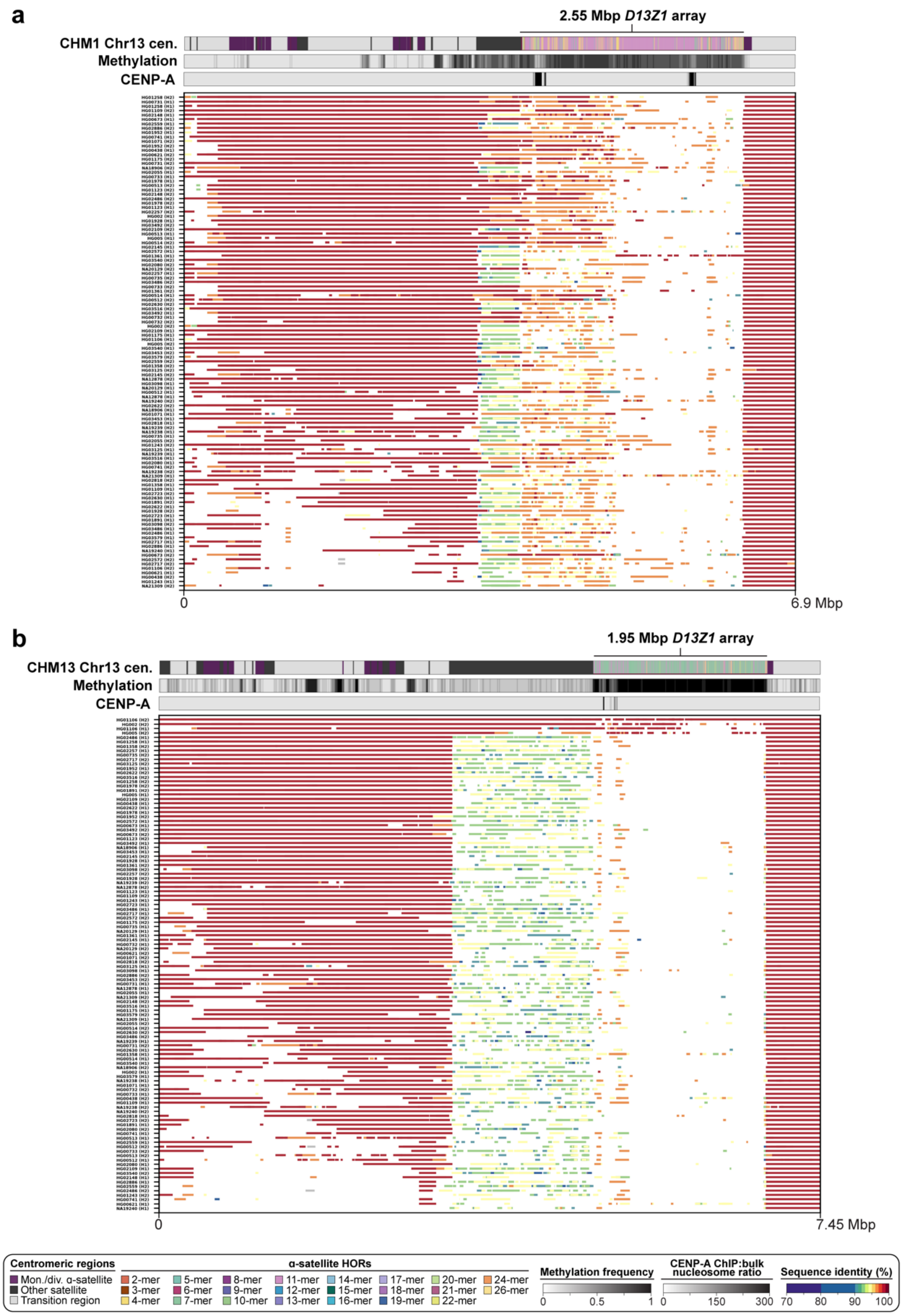


**Supplementary Figure 32. Comparison of the sequence and structure of the chromosome 13 centromeric region from the CHM1, CHM13, and 56 diverse human genomes. a,b)** Plots showing the sequence organization (top track), CpG methylation frequency (second track), CENP-A nucleosome enrichment (third track), and percent sequence identity of contigs from 56 diverse human genomes (112 haplotypes[18,19]) relative to the **a)** CHM1 and **b)** CHM13 genomes.

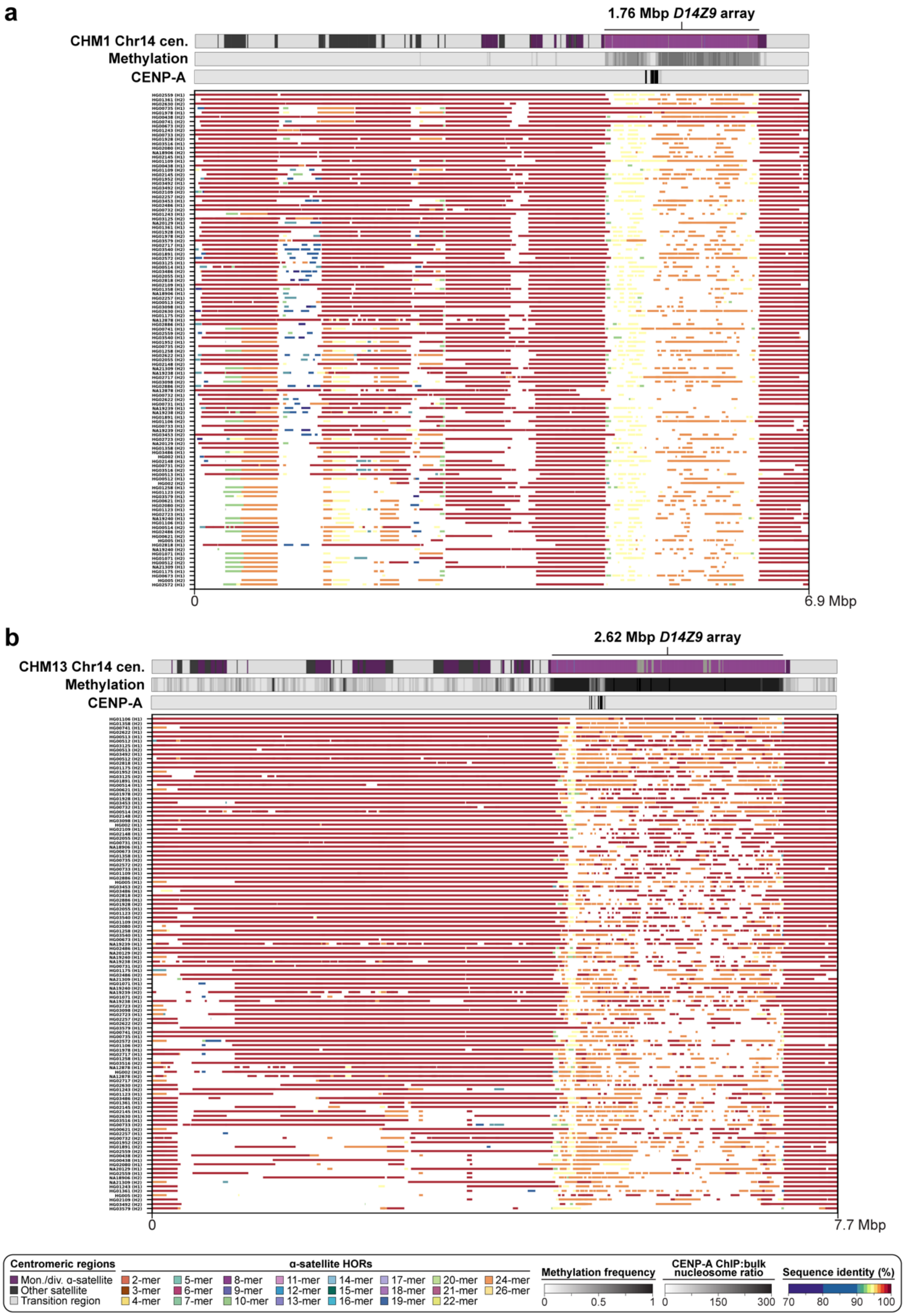


**Supplementary Figure 33. Comparison of the sequence and structure of the chromosome 14 centromeric region from the CHM1, CHM13, and 56 diverse human genomes. a,b)** Plots showing the sequence organization (top track), CpG methylation frequency (second track), CENP-A nucleosome enrichment (third track), and percent sequence identity of contigs from 56 diverse human genomes (112 haplotypes[18,19]) relative to the **a)** CHM1 and **b)** CHM13 genomes.

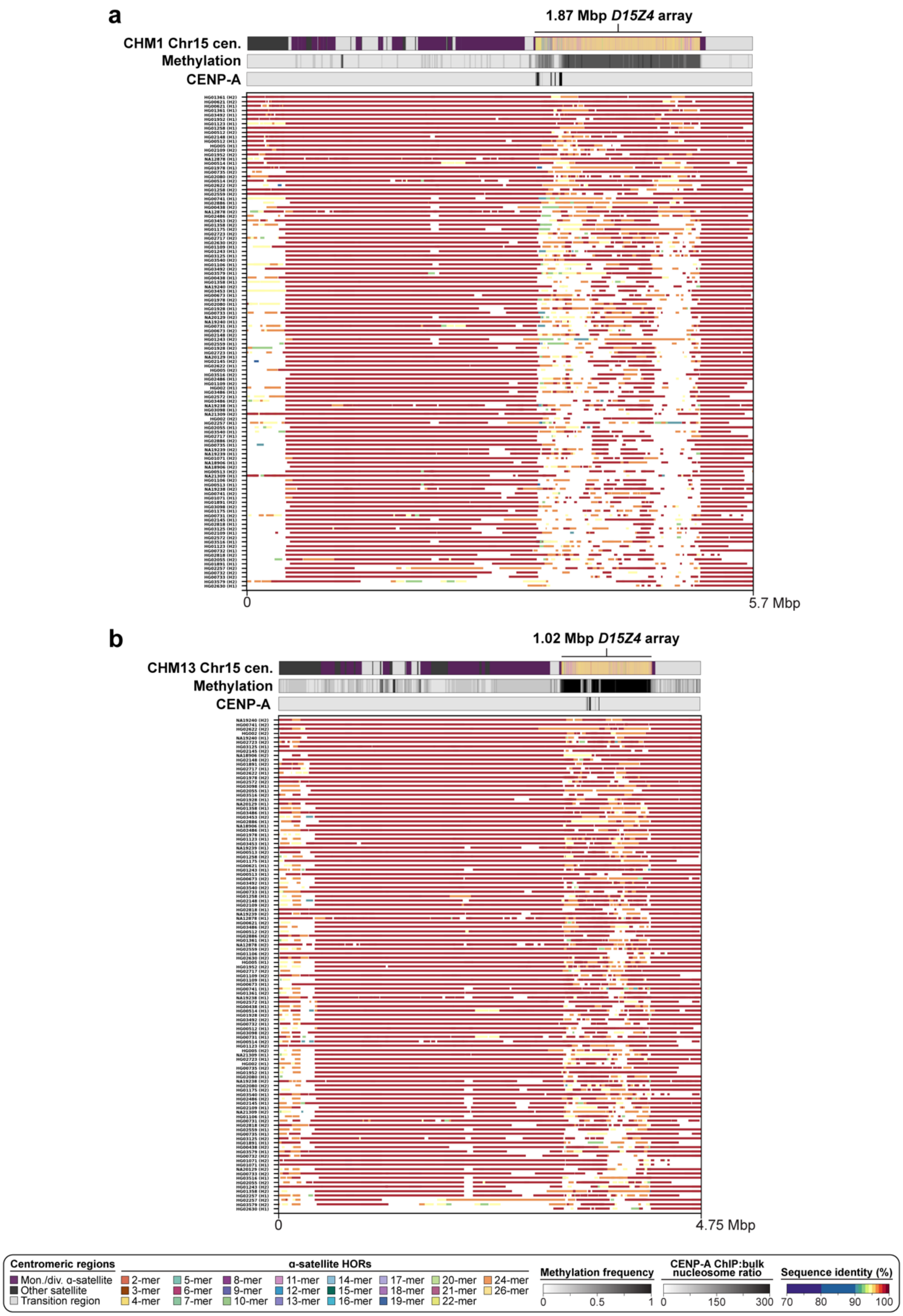


**Supplementary Figure 34. Comparison of the sequence and structure of the chromosome 15 centromeric region from the CHM1, CHM13, and 56 diverse human genomes. a,b)** Plots showing the sequence organization (top track), CpG methylation frequency (second track), CENP-A nucleosome enrichment (third track), and percent sequence identity of contigs from 56 diverse human genomes (112 haplotypes[18,19]) relative to the **a)** CHM1 and **b)** CHM13 genomes.

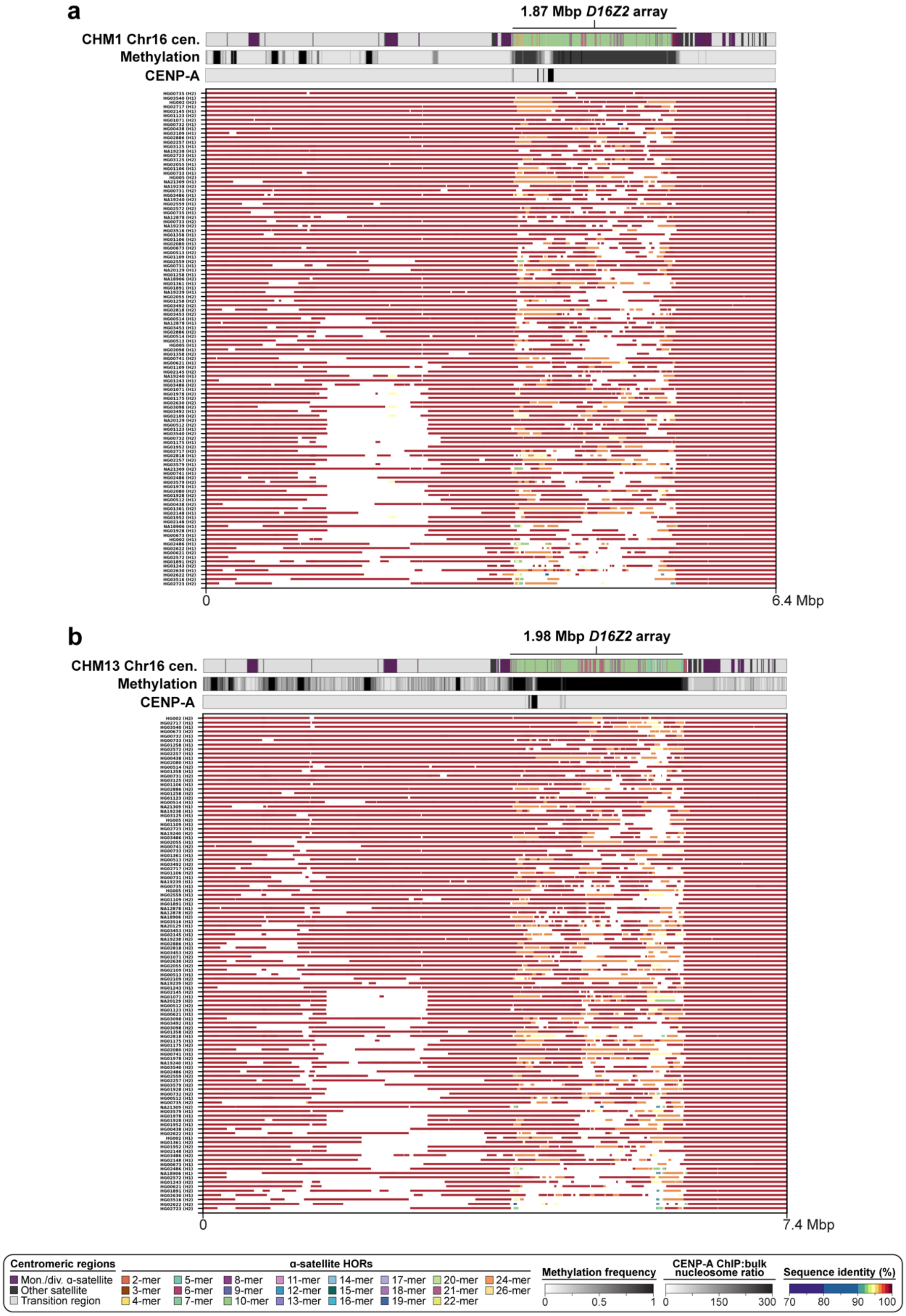


**Supplementary Figure 35. Comparison of the sequence and structure of the chromosome 16 centromeric region from the CHM1, CHM13, and 56 diverse human genomes. a,b)** Plots showing the sequence organization (top track), CpG methylation frequency (second track), CENP-A nucleosome enrichment (third track), and percent sequence identity of contigs from 56 diverse human genomes (112 haplotypes[18,19]) relative to the **a)** CHM1 and **b)** CHM13 genomes.

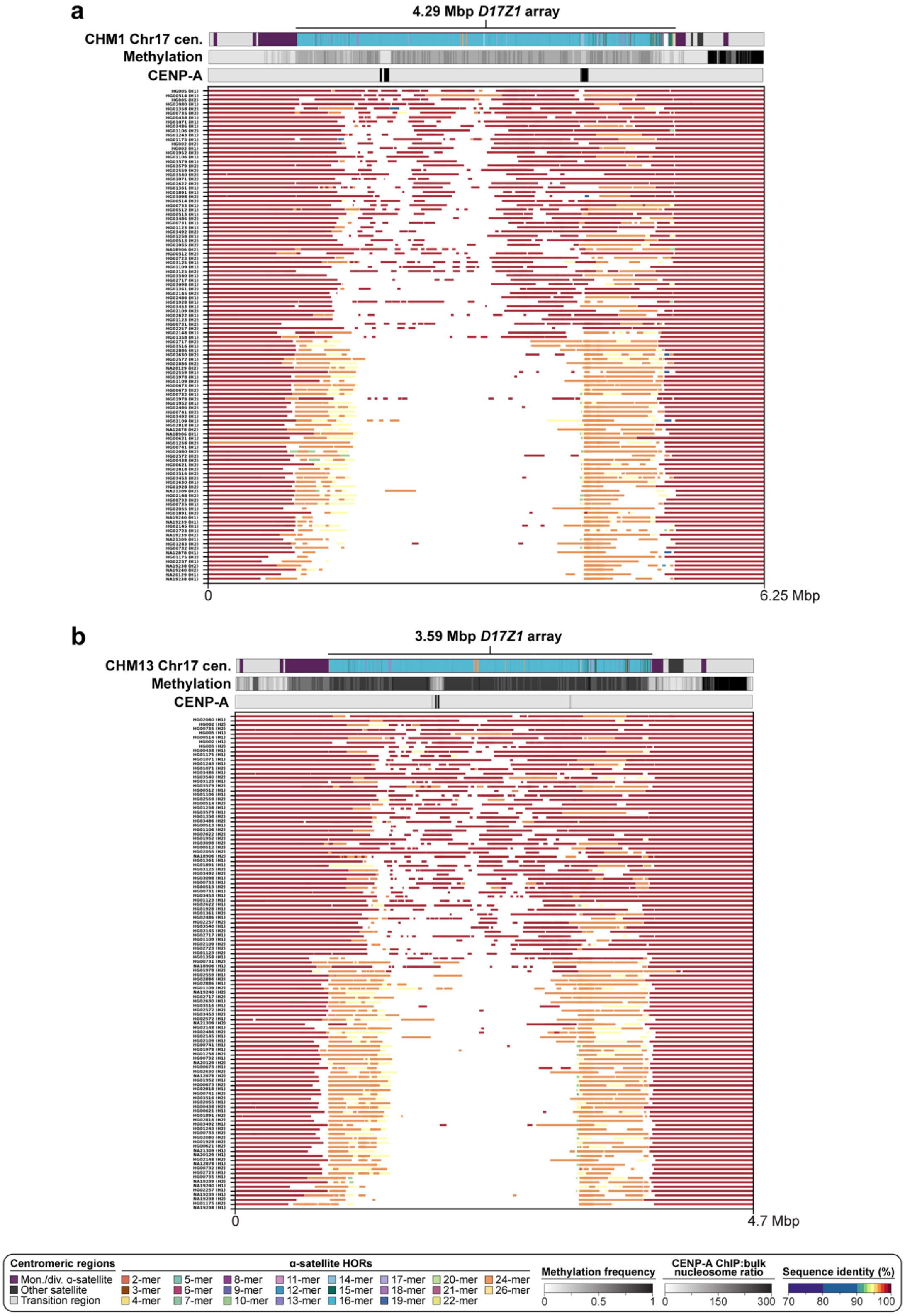


**Supplementary Figure 36. Comparison of the sequence and structure of the chromosome 17 centromeric region from the CHM1, CHM13, and 56 diverse human genomes. a,b)** Plots showing the sequence organization (top track), CpG methylation frequency (second track), CENP-A nucleosome enrichment (third track), and percent sequence identity of contigs from 56 diverse human genomes (112 haplotypes[18,19]) relative to the **a)** CHM1 and **b)** CHM13 genomes.

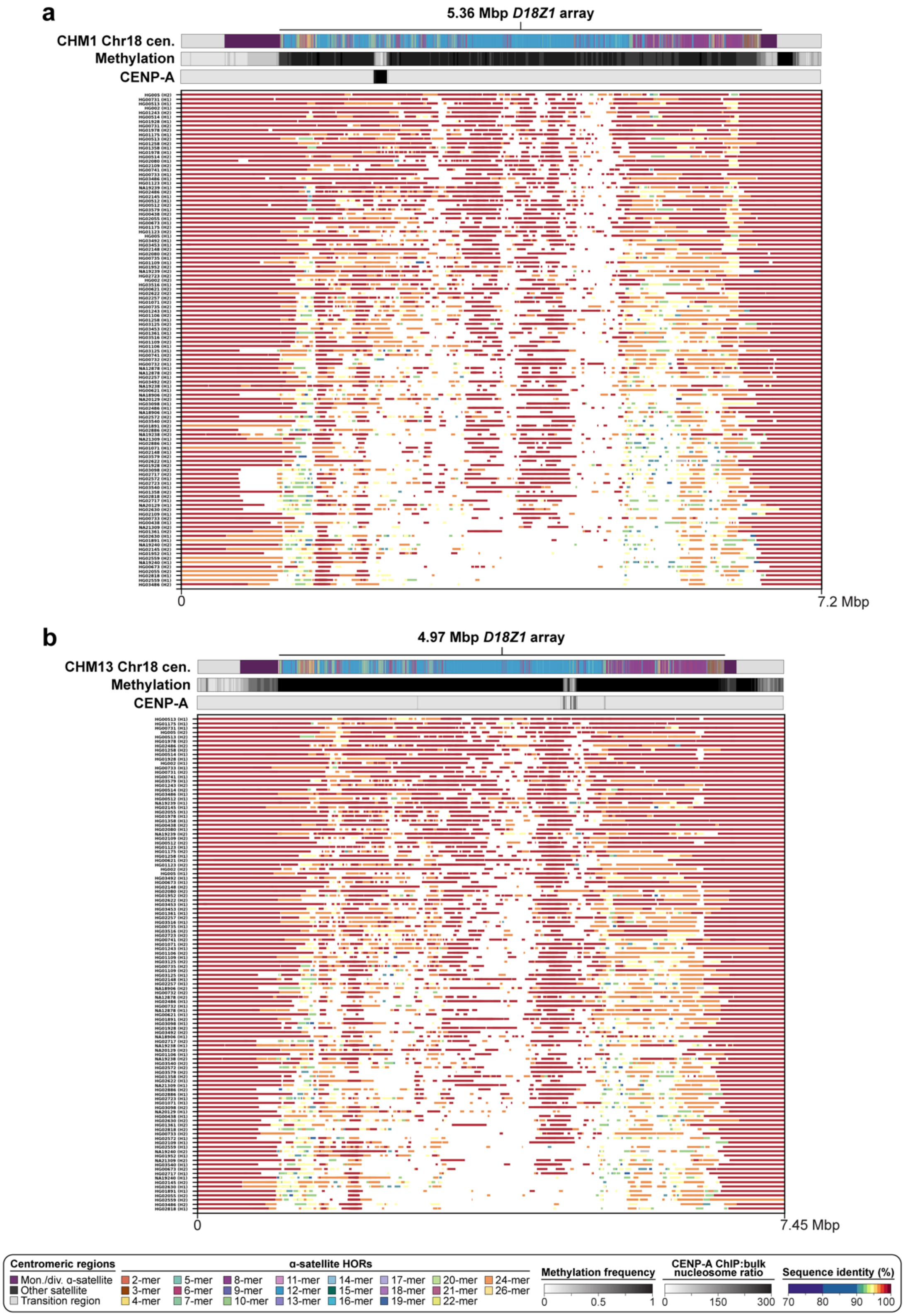


**Supplementary Figure 37. Comparison of the sequence and structure of the chromosome 18 centromeric region from the CHM1, CHM13, and 56 diverse human genomes. a,b)** Plots showing the sequence organization (top track), CpG methylation frequency (second track), CENP-A nucleosome enrichment (third track), and percent sequence identity of contigs from 56 diverse human genomes (112 haplotypes[18,19]) relative to the **a)** CHM1 and **b)** CHM13 genomes.

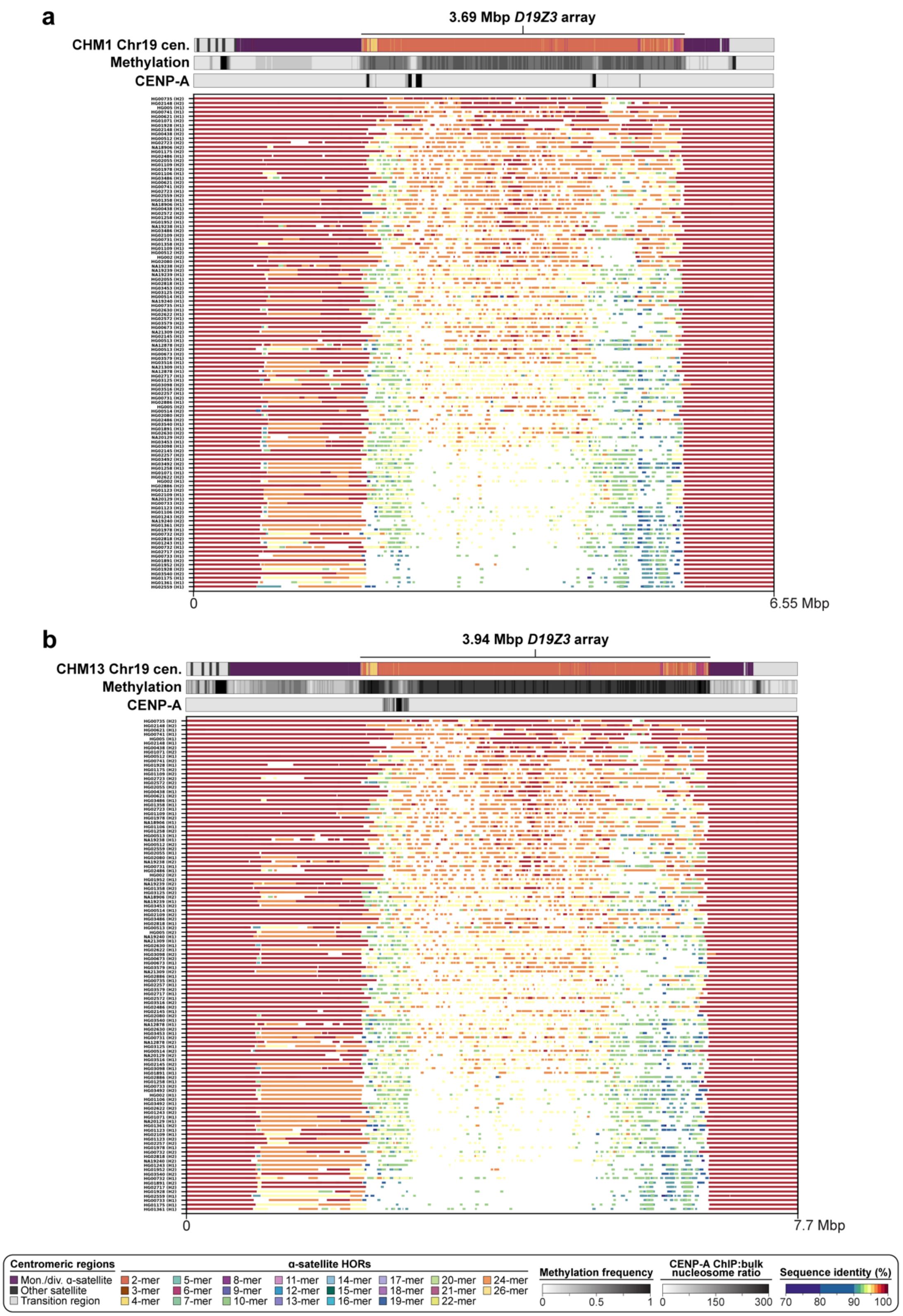


**Supplementary Figure 38. Comparison of the sequence and structure of the chromosome 19 centromeric region from the CHM1, CHM13, and 56 diverse human genomes. a,b)** Plots showing the sequence organization (top track), CpG methylation frequency (second track), CENP-A nucleosome enrichment (third track), and percent sequence identity of contigs from 56 diverse human genomes (112 haplotypes[18,19]) relative to the **a)** CHM1 and **b)** CHM13 genomes.

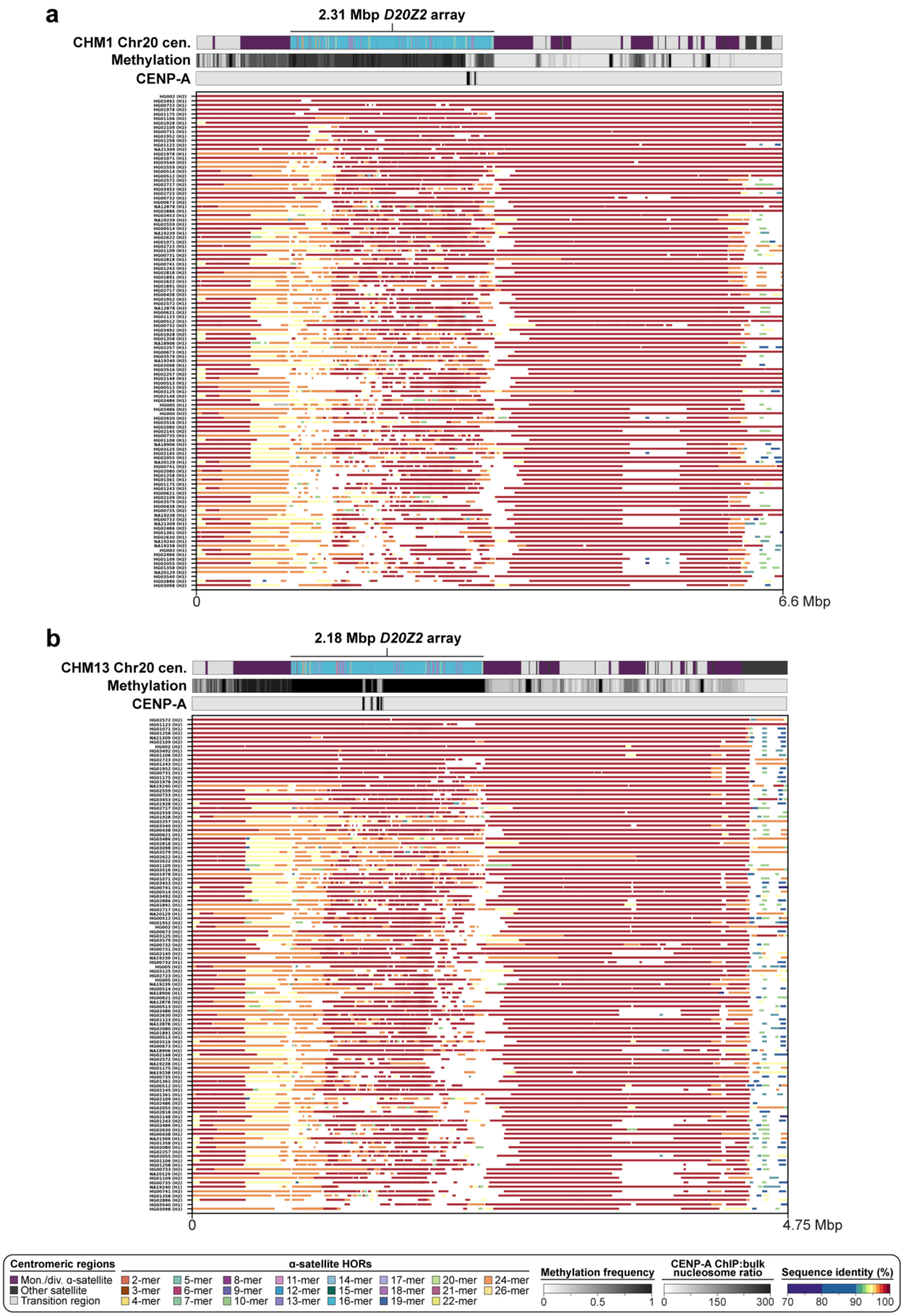


**Supplementary Figure 39. Comparison of the sequence and structure of the chromosome 20 centromeric region from the CHM1, CHM13, and 56 diverse human genomes. a,b)** Plots showing the sequence organization (top track), CpG methylation frequency (second track), CENP-A nucleosome enrichment (third track), and percent sequence identity of contigs from 56 diverse human genomes (112 haplotypes[18,19]) relative to the **a)** CHM1 and **b)** CHM13 genomes.

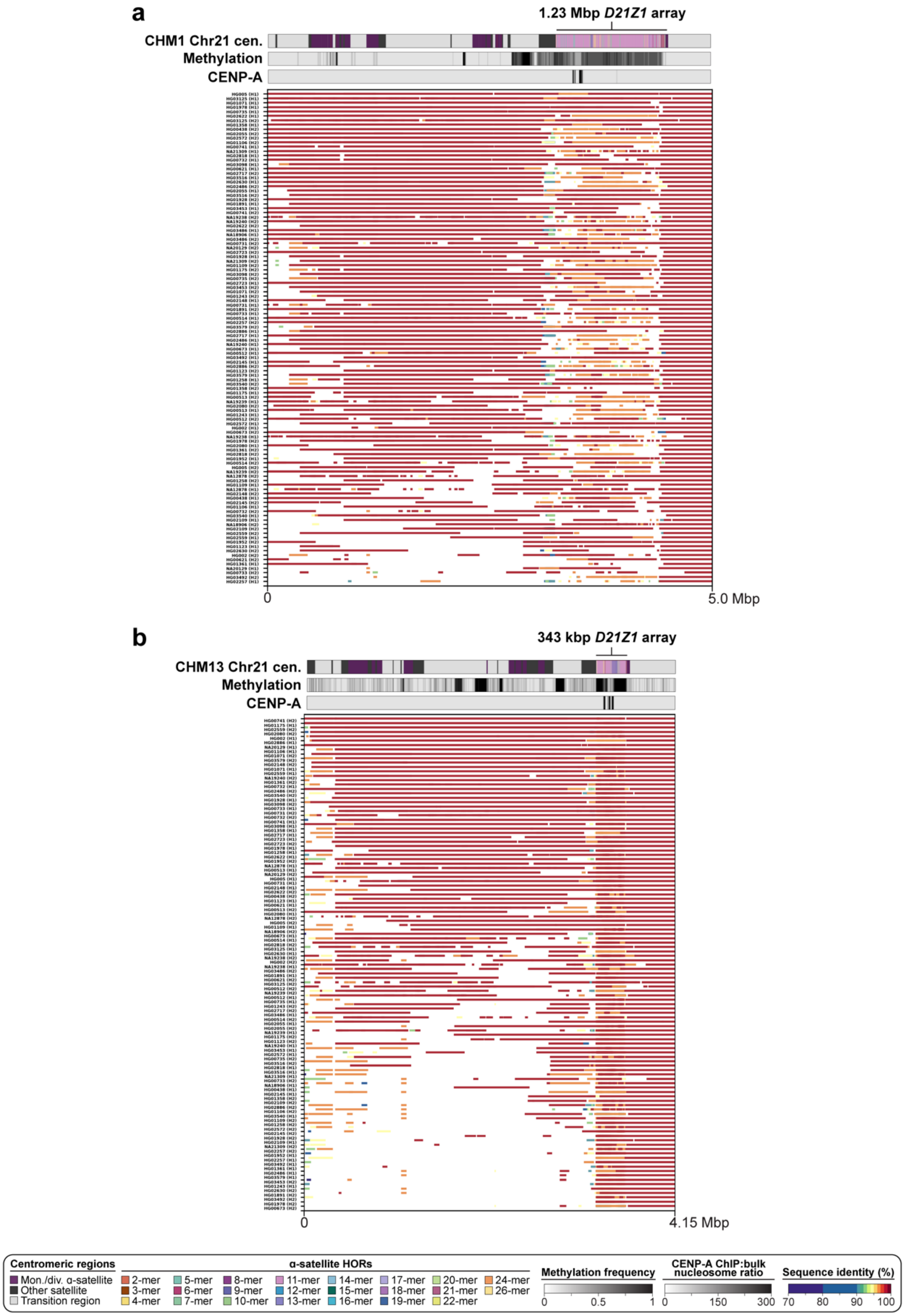


**Supplementary Figure 40. Comparison of the sequence and structure of the chromosome 21 centromeric region from the CHM1, CHM13, and 56 diverse human genomes. a,b)** Plots showing the sequence organization (top track), CpG methylation frequency (second track), CENP-A nucleosome enrichment (third track), and percent sequence identity of contigs from 56 diverse human genomes (112 haplotypes[18,19]) relative to the **a)** CHM1 and **b)** CHM13 genomes.

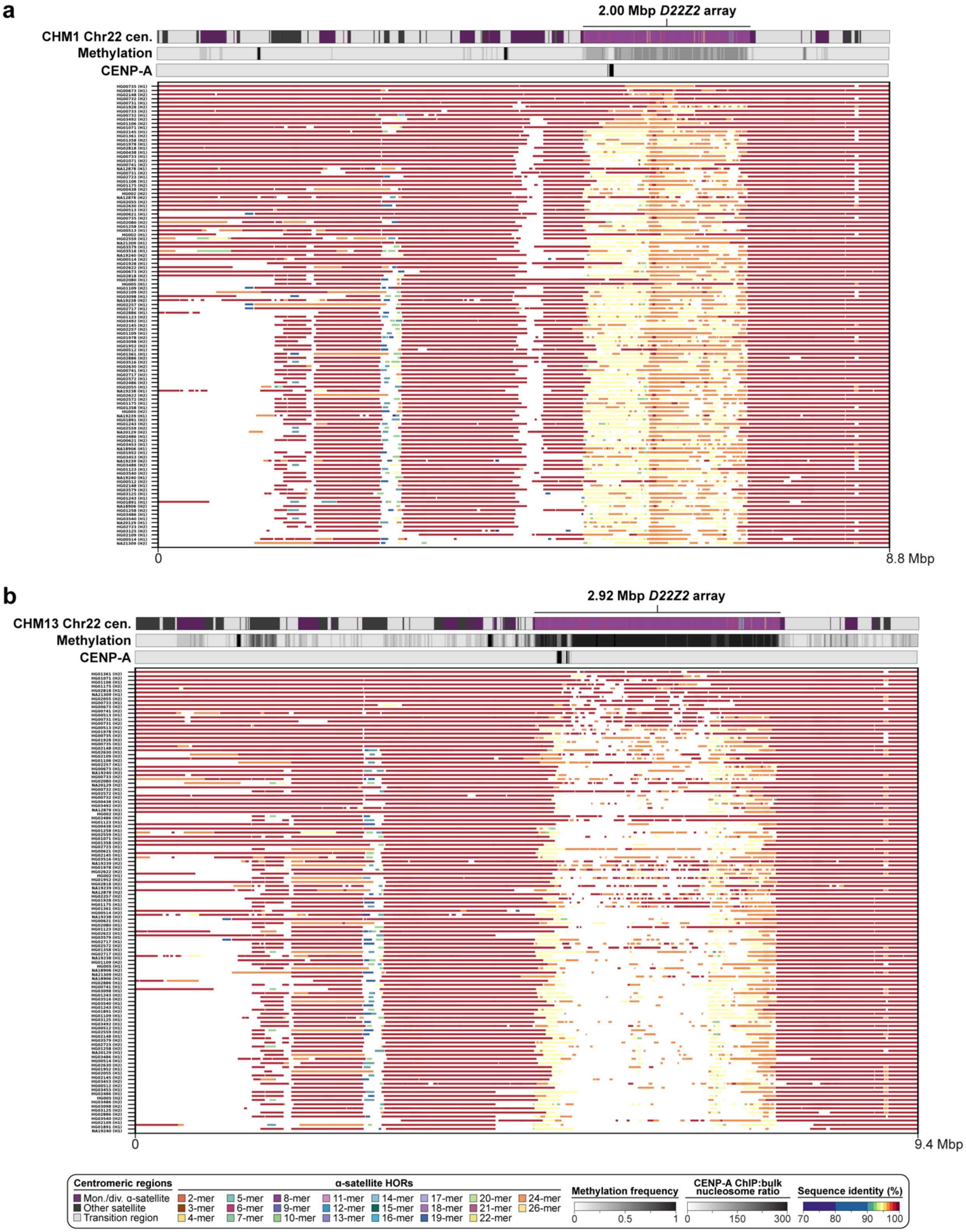


**Supplementary Figure 41. Comparison of the sequence and structure of the chromosome 22 centromeric region from the CHM1, CHM13, and 56 diverse human genomes. a,b)** Plots showing the sequence organization (top track), CpG methylation frequency (second track), CENP-A nucleosome enrichment (third track), and percent sequence identity of contigs from 56 diverse human genomes (112 haplotypes[18,19]) relative to the **a)** CHM1 and **b)** CHM13 genomes.

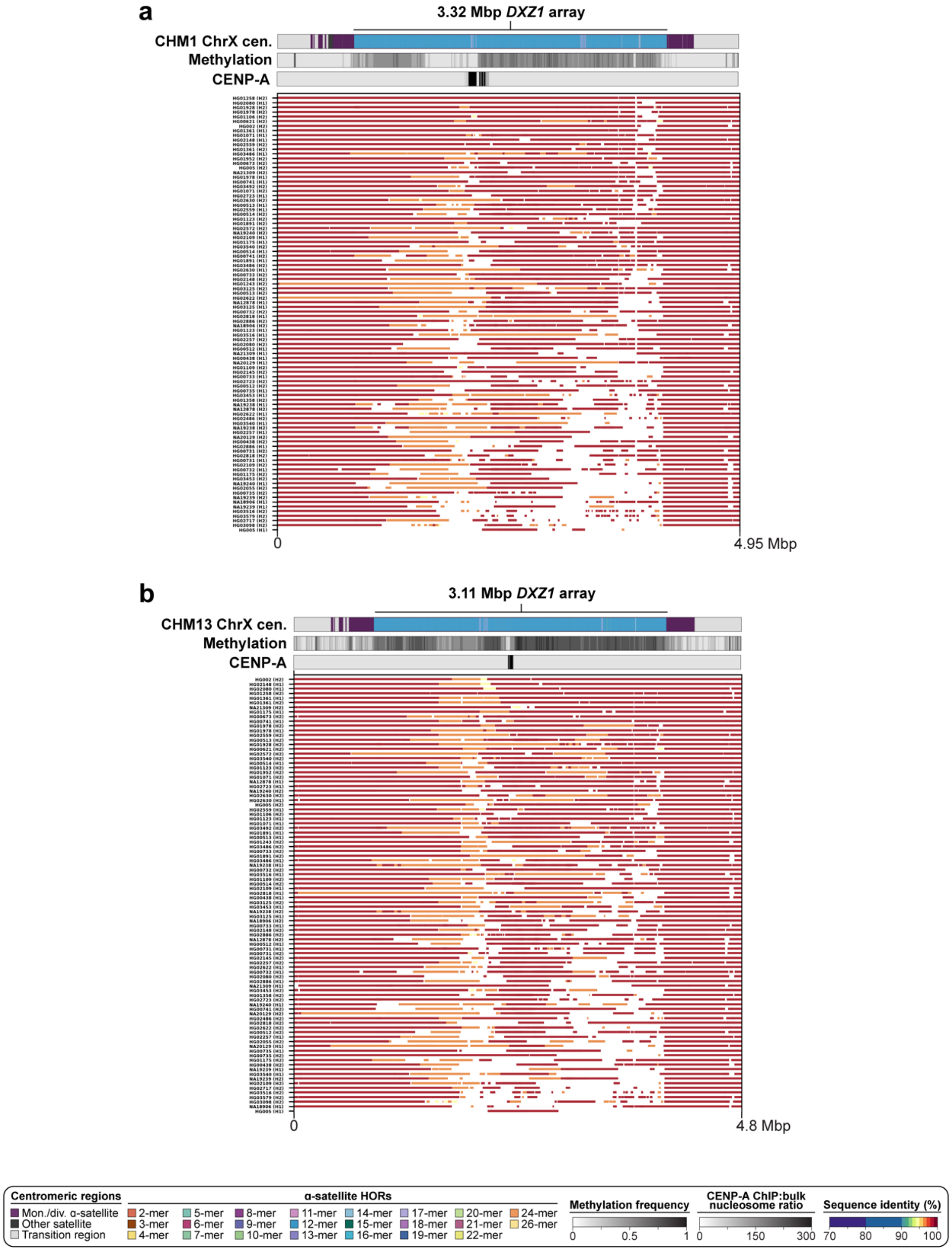


**Supplementary Figure 42. Comparison of the sequence and structure of the chromosome X centromeric region from the CHM1, CHM13, and 56 diverse human genomes. a,b)** Plots showing the sequence organization (top track), CpG methylation frequency (second track), CENP-A nucleosome enrichment (third track), and percent sequence identity of contigs from 56 diverse human genomes (112 haplotypes[18,19]) relative to the **a)** CHM1 and **b)** CHM13 genomes.

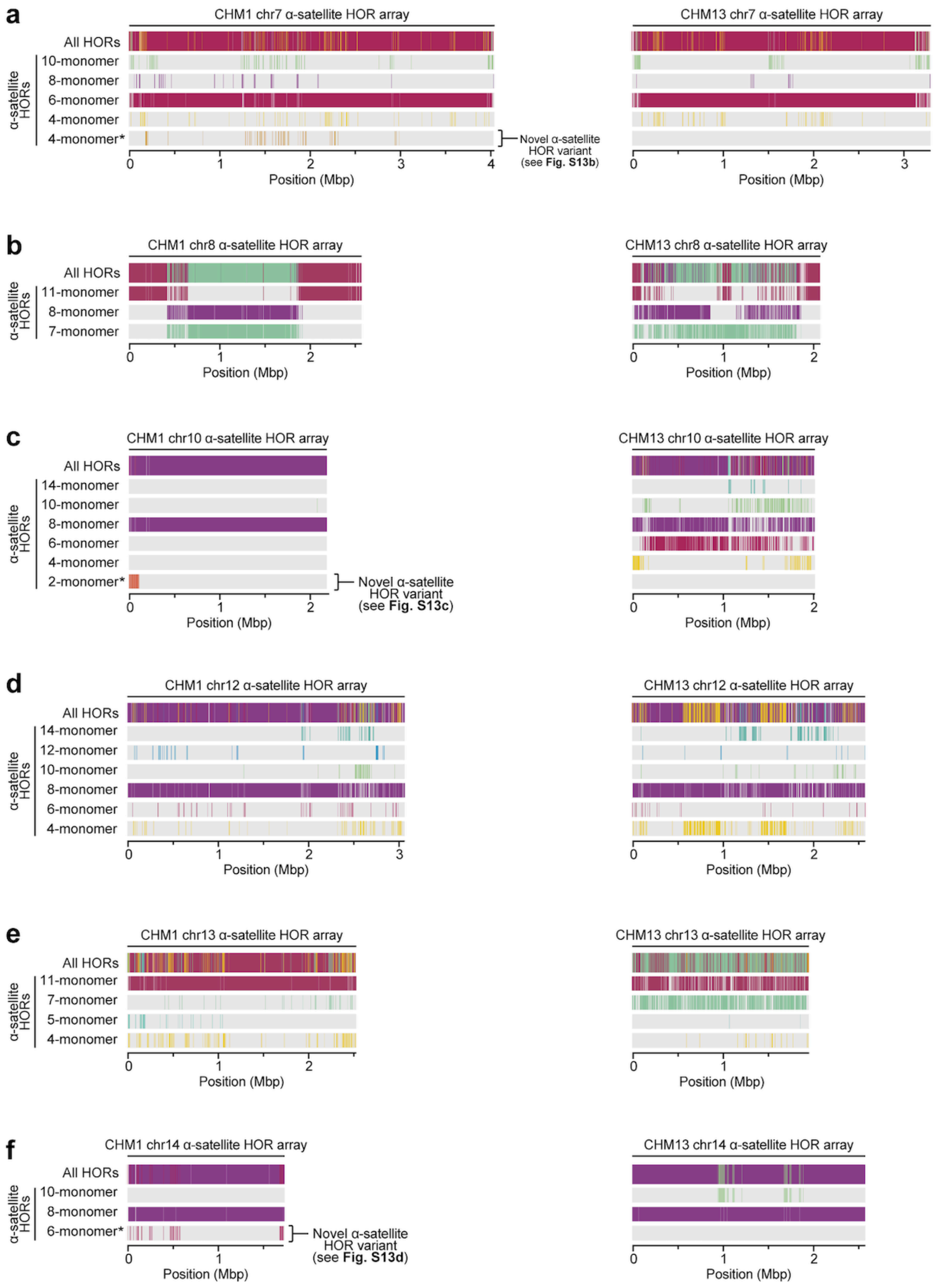


**Supplementary Figure 43. Variation in the sequence and structure of the α-satellite HOR arrays between the CHM1 and CHM13 centromeres. a-f)** Structure of the CHM1 (left) and CHM13 (right) α-satellite HOR arrays from chromosomes **a)** 7, **b)** 8, **c)** 10, **d)** 12, **e)** 13, and **f)** 14. Novel α-satellite HOR variants are indicated.

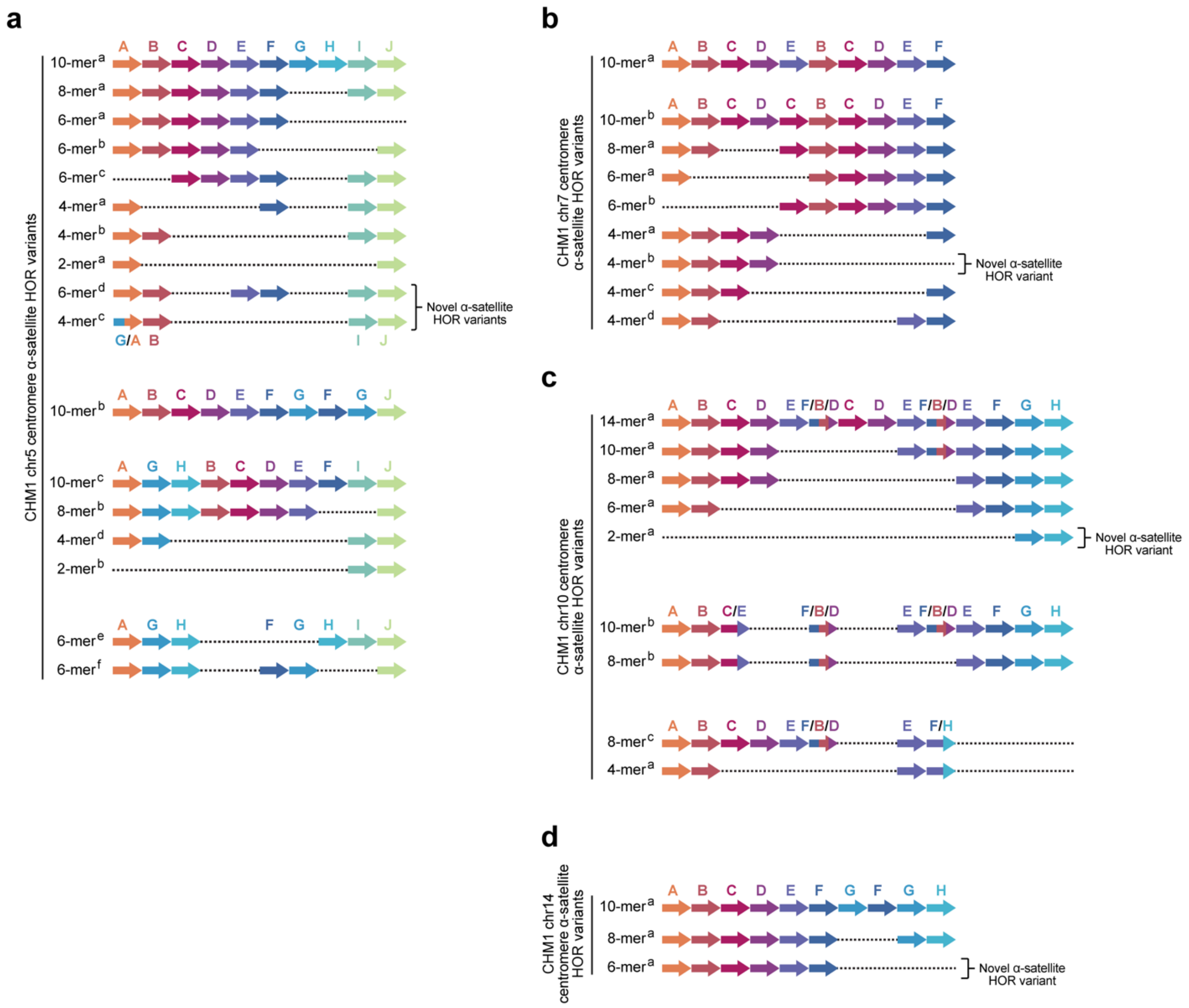


**Supplementary Figure 44. Novel α-satellite HOR variants within the CHM1 centromeres. a-d)** Structures of the α-satellite HOR variants within the CHM1 centromeres from chromosomes **a)** 5, **b)** 7, **c)** 10, and **d)** 14. Novel α-satellite HOR variants are indicated.

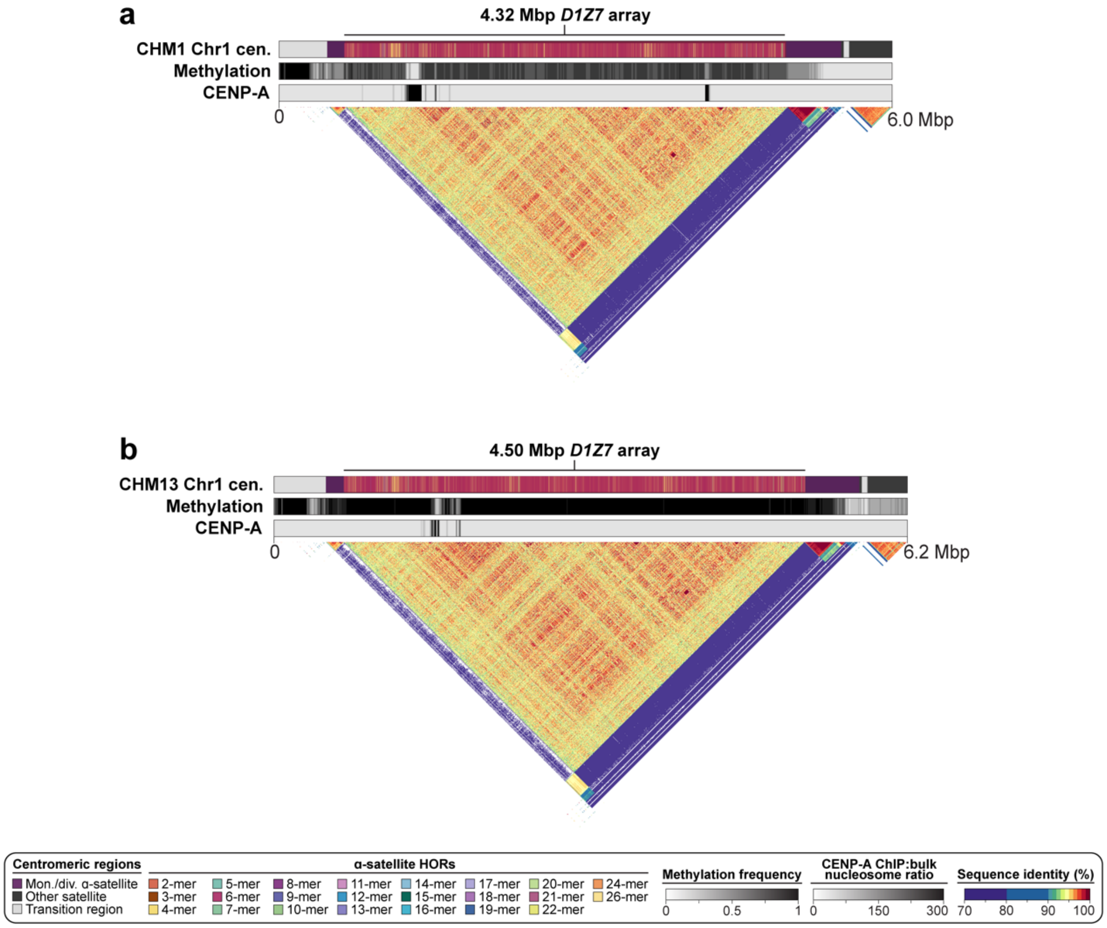


**Supplementary Figure 45. Comparison of the genetic, epigenetic, and evolutionary landscapes between the CHM1 and CHM13 chromosome 1 centromeric regions.** Plots showing the sequence organization (top track), CpG methylation frequency (second track), CENP-A nucleosome enrichment (third track), and evolutionary layers (bottom triangle) for the **a)** CHM1 and **b)** CHM13 chromosome 1 centromeric regions.

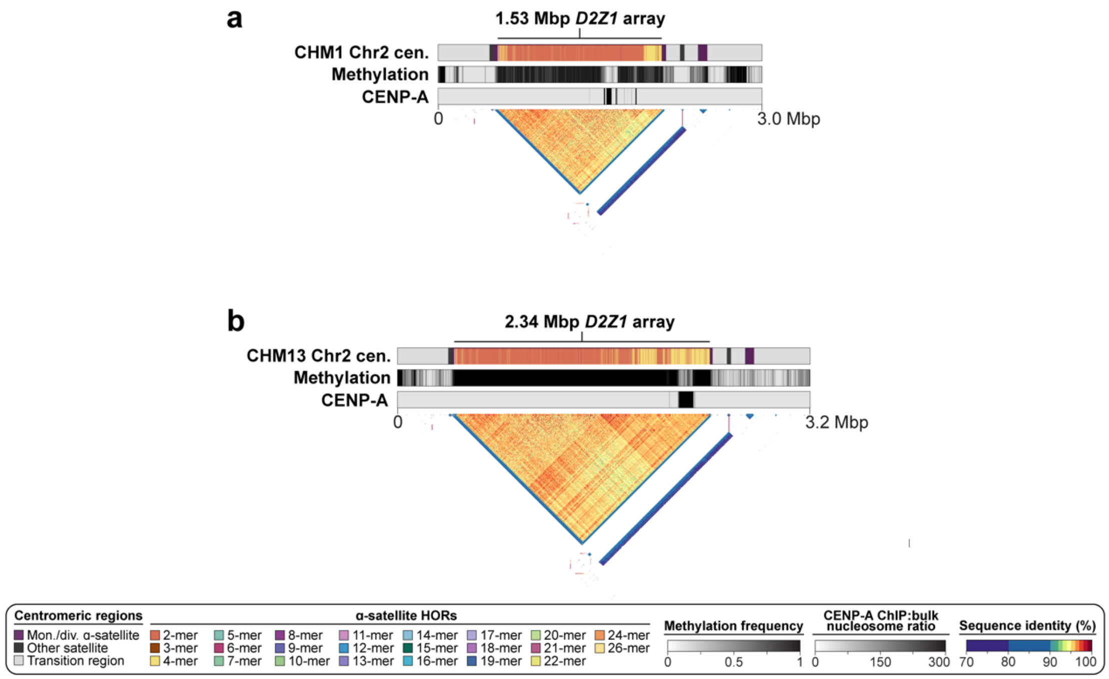


**Supplementary Figure 46. Comparison of the genetic, epigenetic, and evolutionary landscapes between the CHM1 and CHM13 chromosome 2 centromeric regions.** Plots showing the sequence organization (top track), CpG methylation frequency (second track), CENP-A nucleosome enrichment (third track), and evolutionary layers (bottom triangle) for the **a)** CHM1 and **b)** CHM13 chromosome 2 centromeric regions.

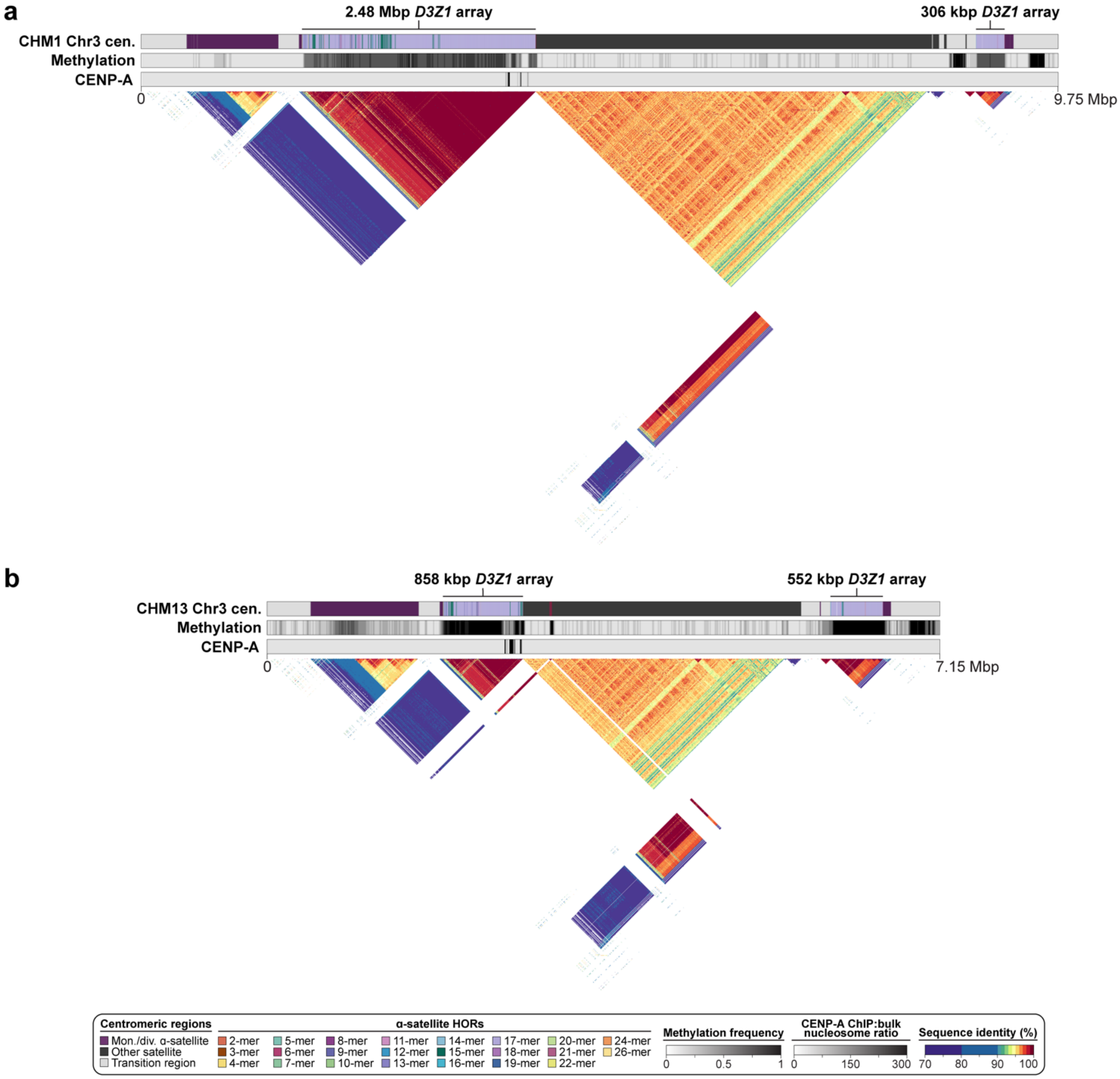


**Supplementary Figure 47. Comparison of the genetic, epigenetic, and evolutionary landscapes between the CHM1 and CHM13 chromosome 3 centromeric regions.** Plots showing the sequence organization (top track), CpG methylation frequency (second track), CENP-A nucleosome enrichment (third track), and evolutionary layers (bottom triangle) for the **a)** CHM1 and **b)** CHM13 chromosome 3 centromeric regions.

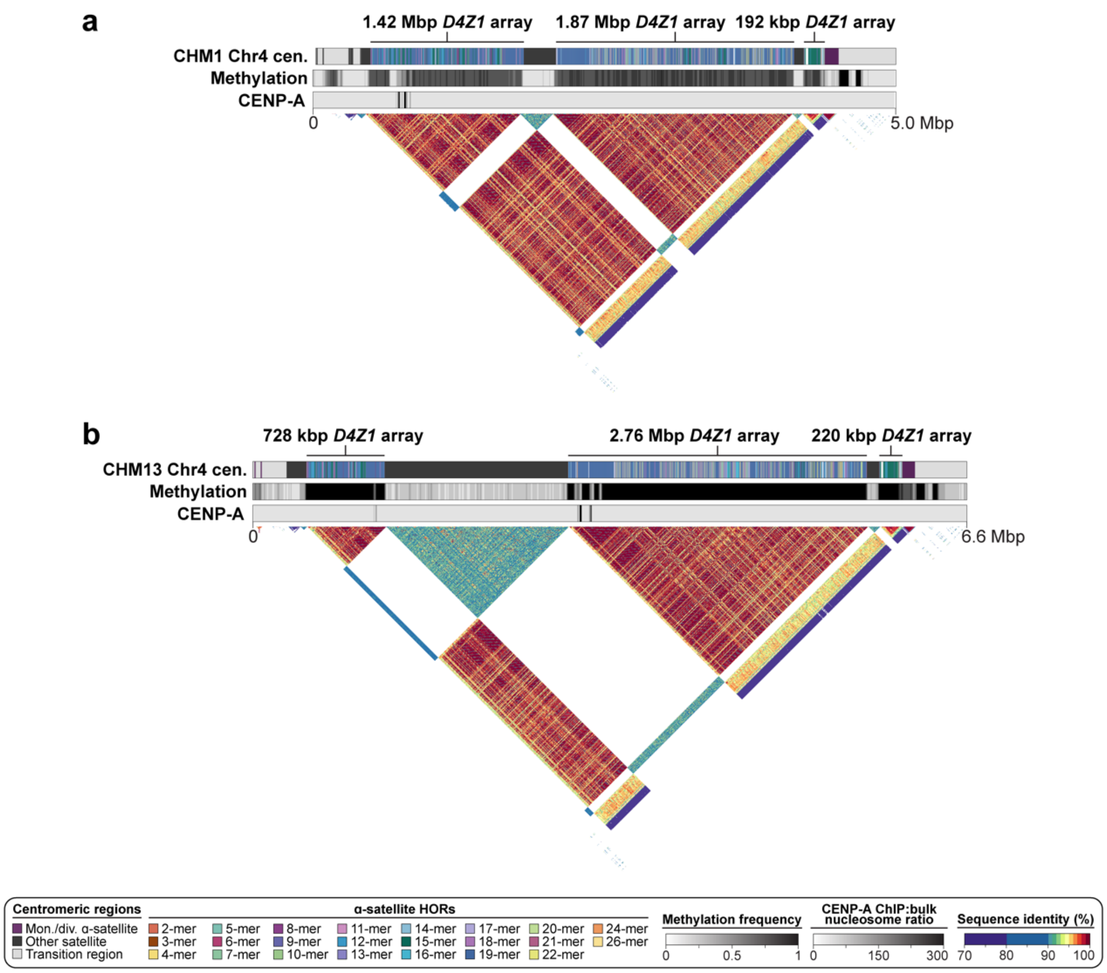


**Supplementary Figure 48. Comparison of the genetic, epigenetic, and evolutionary landscapes between the CHM1 and CHM13 chromosome 4 centromeric regions.** Plots showing the sequence organization (top track), CpG methylation frequency (second track), CENP-A nucleosome enrichment (third track), and evolutionary layers (bottom triangle) for the **a)** CHM1 and **b)** CHM13 chromosome 4 centromeric regions.

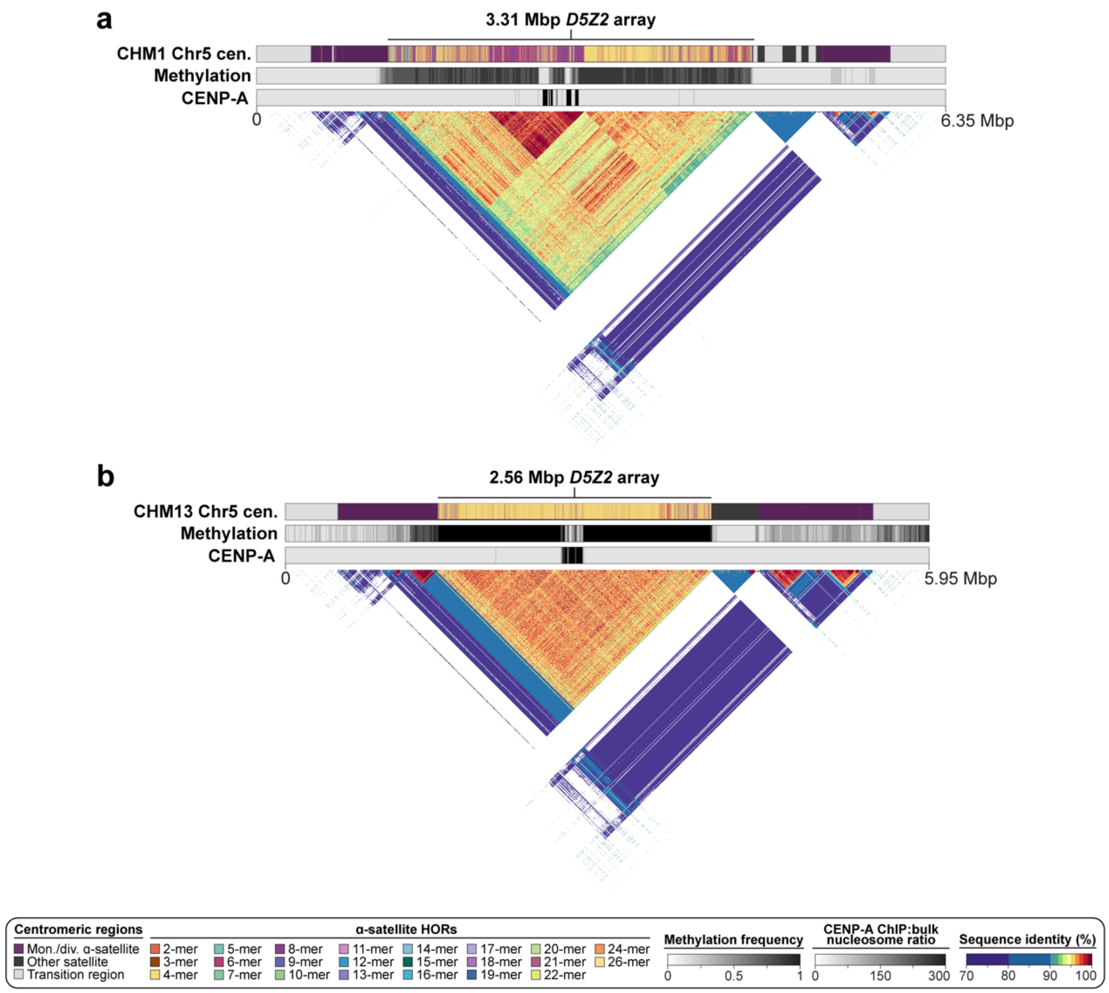


**Supplementary Figure 49. Comparison of the genetic, epigenetic, and evolutionary landscapes between the CHM1 and CHM13 chromosome 5 centromeric regions.** Plots showing the sequence organization (top track), CpG methylation frequency (second track), CENP-A nucleosome enrichment (third track), and evolutionary layers (bottom triangle) for the **a)** CHM1 and **b)** CHM13 chromosome 5 centromeric regions.

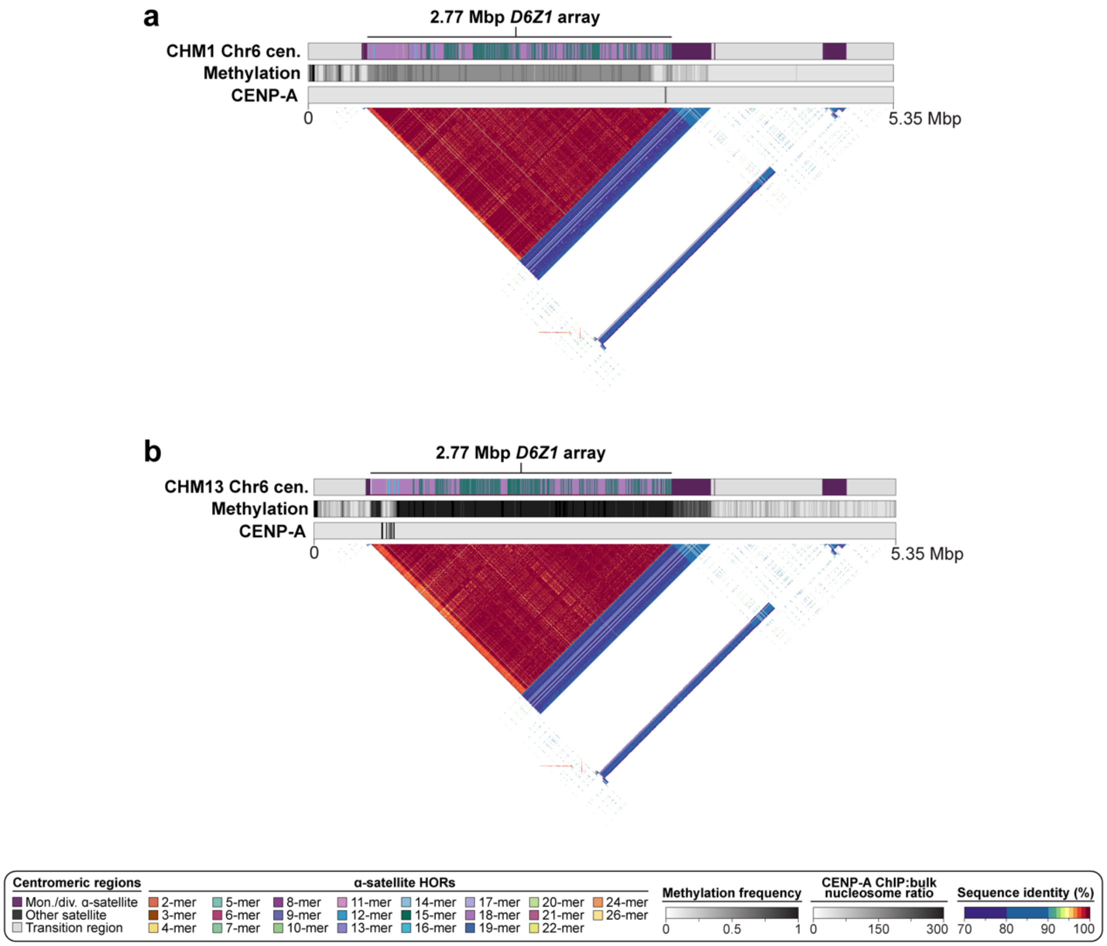


**Supplementary Figure 50. Comparison of the genetic, epigenetic, and evolutionary landscapes between the CHM1 and CHM13 chromosome 6 centromeric regions.** Plots showing the sequence organization (top track), CpG methylation frequency (second track), CENP-A nucleosome enrichment (third track), and evolutionary layers (bottom triangle) for the **a)** CHM1 and **b)** CHM13 chromosome 6 centromeric regions.

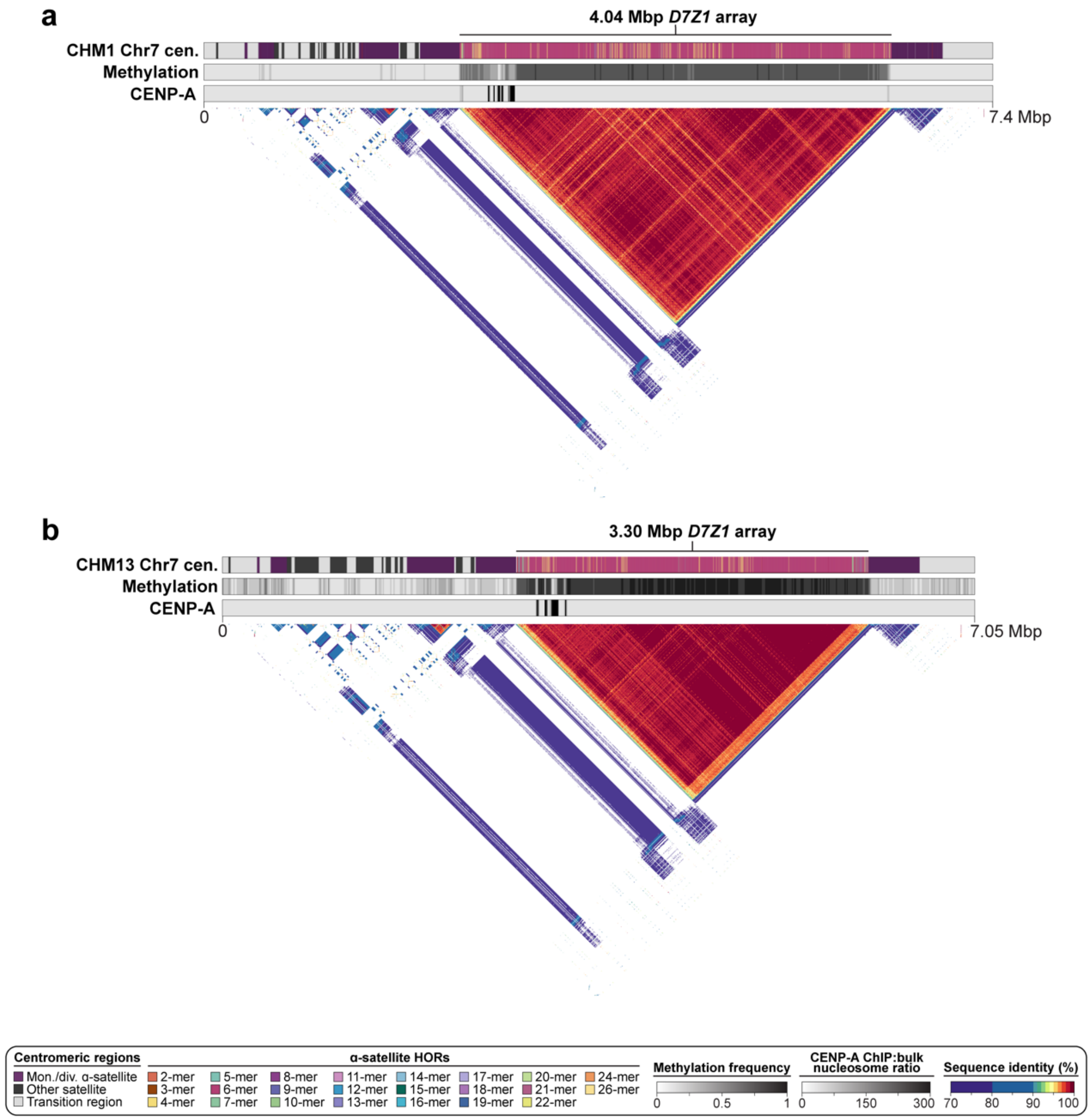


**Supplementary Figure 51. Comparison of the genetic, epigenetic, and evolutionary landscapes between the CHM1 and CHM13 chromosome 7 centromeric regions.** Plots showing the sequence organization (top track), CpG methylation frequency (second track), CENP-A nucleosome enrichment (third track), and evolutionary layers (bottom triangle) for the **a)** CHM1 and **b)** CHM13 chromosome 7 centromeric regions.

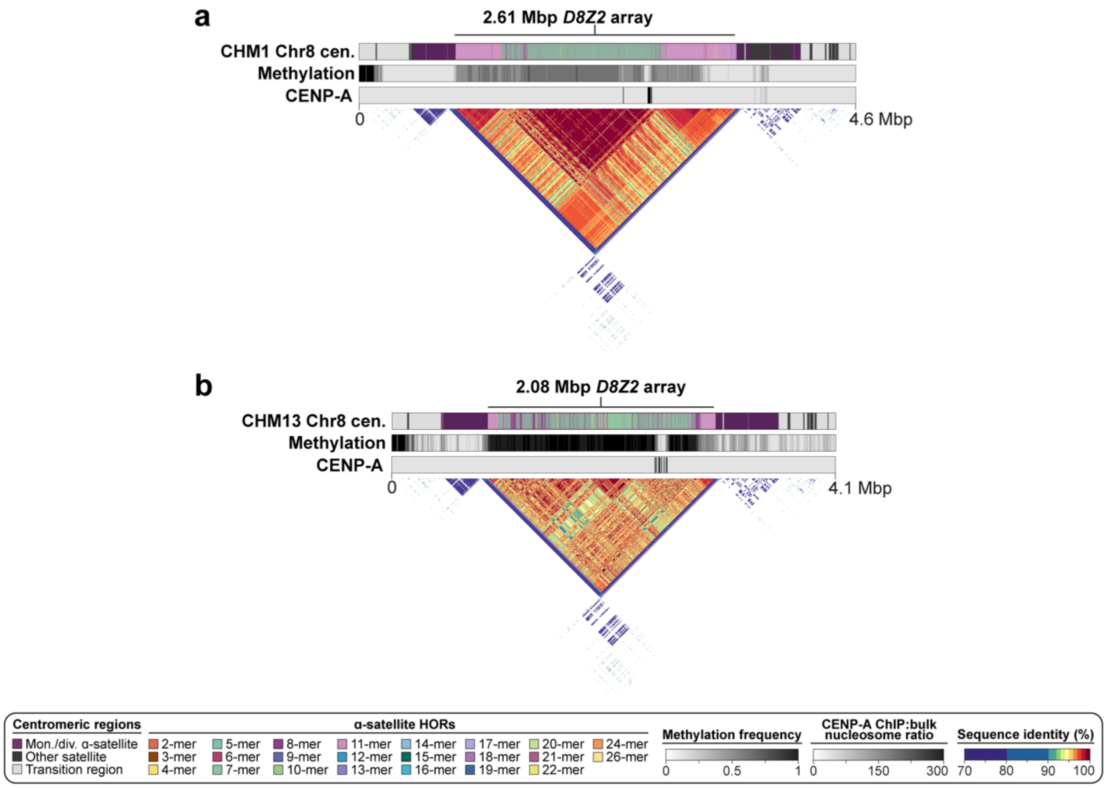


**Supplementary Figure 52. Comparison of the genetic, epigenetic, and evolutionary landscapes between the CHM1 and CHM13 chromosome 8 centromeric regions.** Plots showing the sequence organization (top track), CpG methylation frequency (second track), CENP-A nucleosome enrichment (third track), and evolutionary layers (bottom triangle) for the **a)** CHM1 and **b)** CHM13 chromosome 8 centromeric regions.

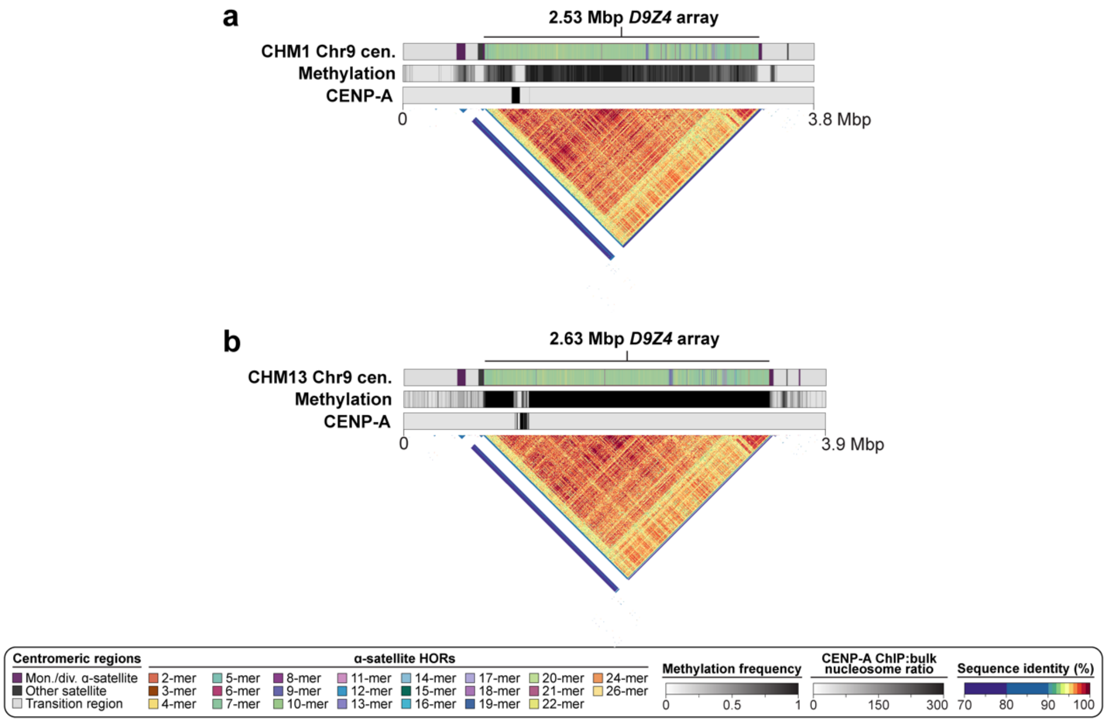


**Supplementary Figure 53. Comparison of the genetic, epigenetic, and evolutionary landscapes between the CHM1 and CHM13 chromosome 9 centromeric regions.** Plots showing the sequence organization (top track), CpG methylation frequency (second track), CENP-A nucleosome enrichment (third track), and evolutionary layers (bottom triangle) for the **a)** CHM1 and **b)** CHM13 chromosome 9 centromeric regions.

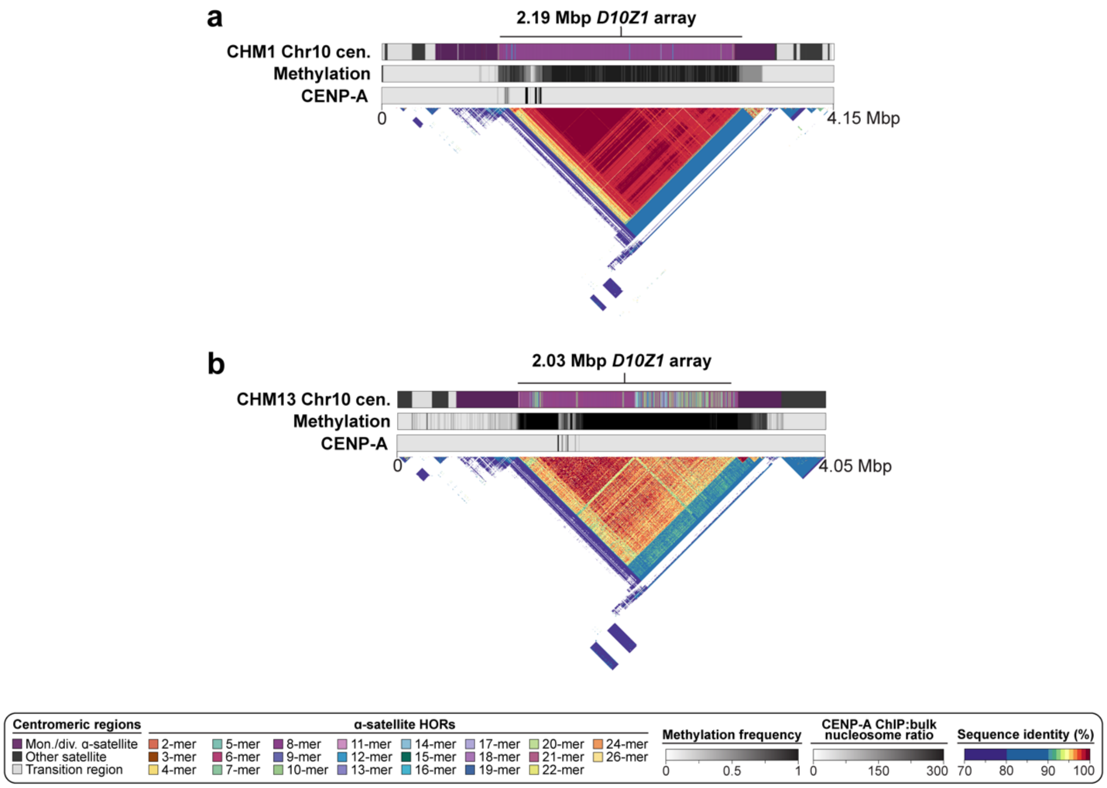


**Supplementary Figure 54. Comparison of the genetic, epigenetic, and evolutionary landscapes between the CHM1 and CHM13 chromosome 10 centromeric regions.** Plots showing the sequence organization (top track), CpG methylation frequency (second track), CENP-A nucleosome enrichment (third track), and evolutionary layers (bottom triangle) for the **a)** CHM1 and **b)** CHM13 chromosome 10 centromeric regions.

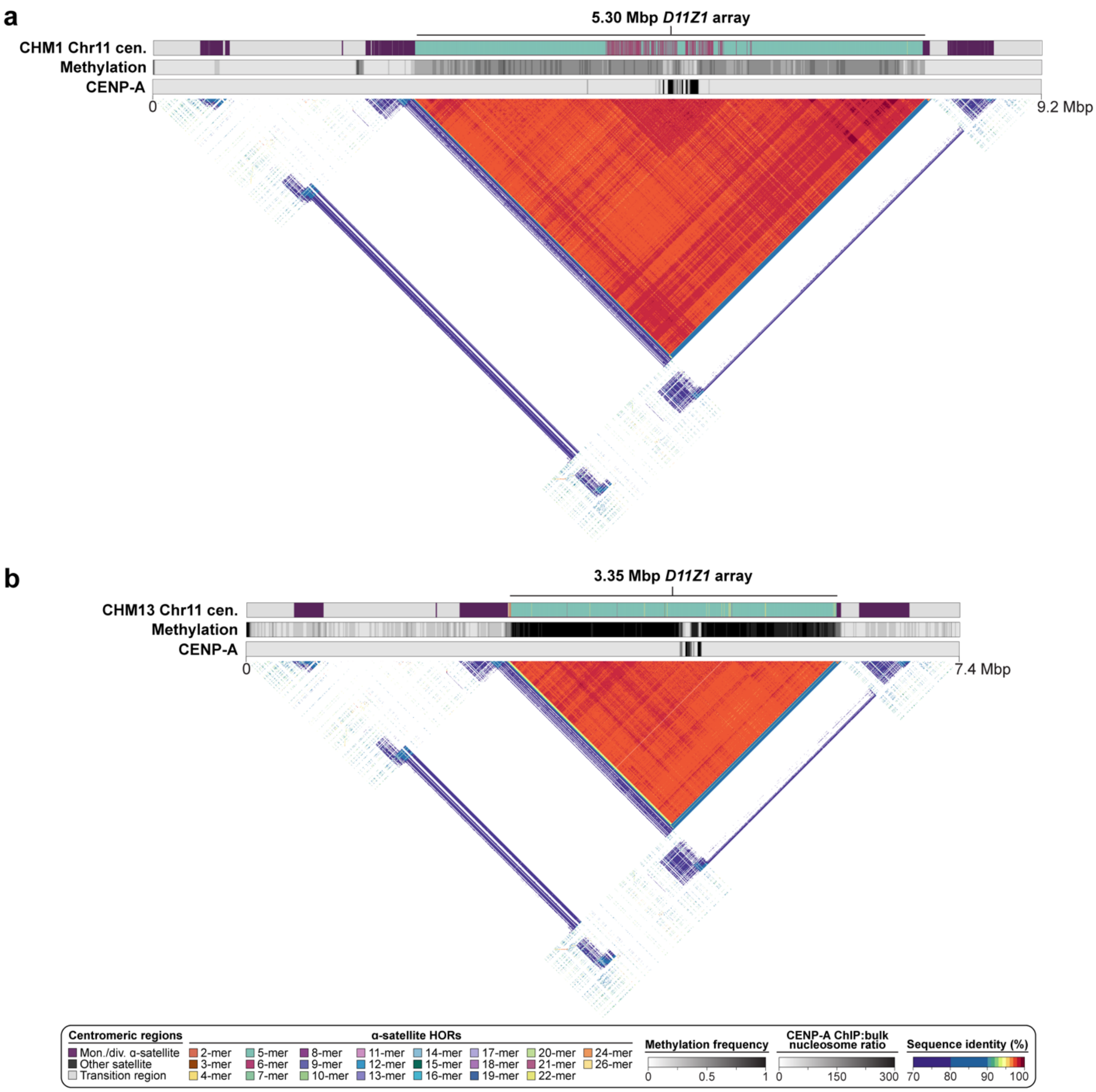


**Supplementary Figure 55. Comparison of the genetic, epigenetic, and evolutionary landscapes between the CHM1 and CHM13 chromosome 11 centromeric regions.** Plots showing the sequence organization (top track), CpG methylation frequency (second track), CENP-A nucleosome enrichment (third track), and evolutionary layers (bottom triangle) for the **a)** CHM1 and **b)** CHM13 chromosome 11 centromeric regions.

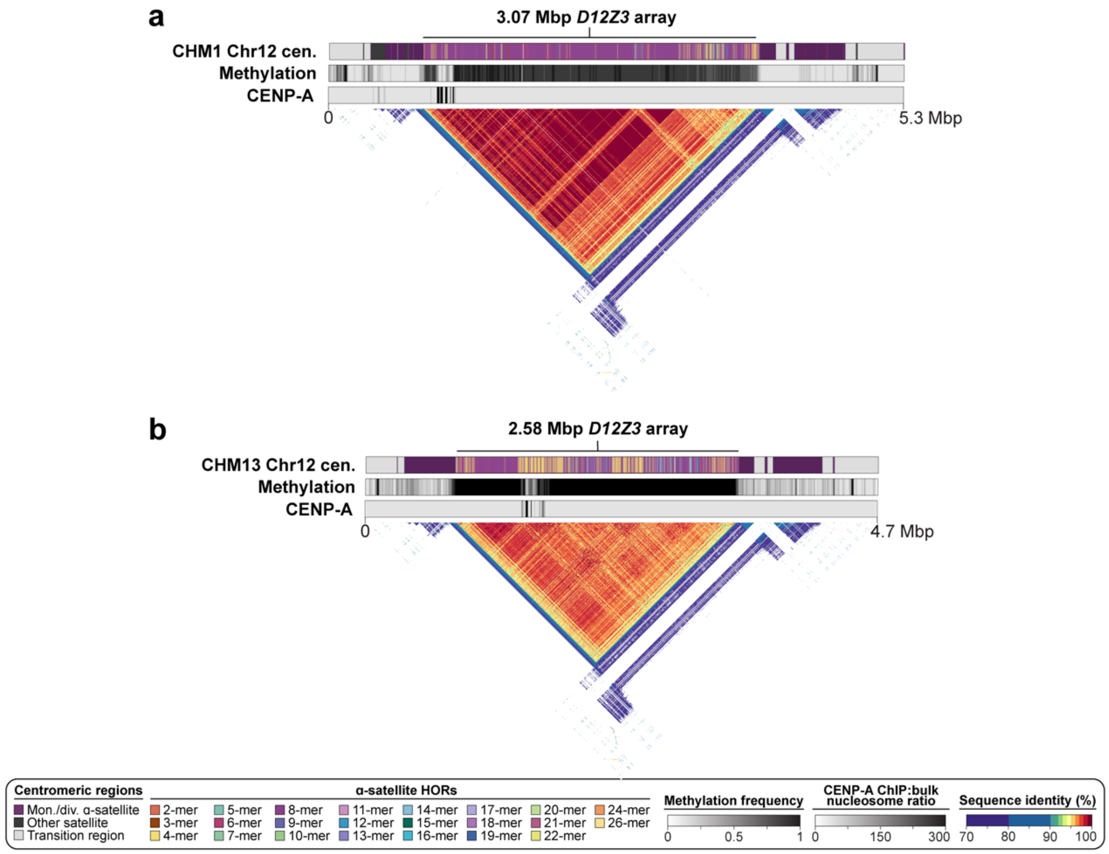


**Supplementary Figure 56. Comparison of the genetic, epigenetic, and evolutionary landscapes between the CHM1 and CHM13 chromosome 12 centromeric regions.** Plots showing the sequence organization (top track), CpG methylation frequency (second track), CENP-A nucleosome enrichment (third track), and evolutionary layers (bottom triangle) for the **a)** CHM1 and **b)** CHM13 chromosome 12 centromeric regions.

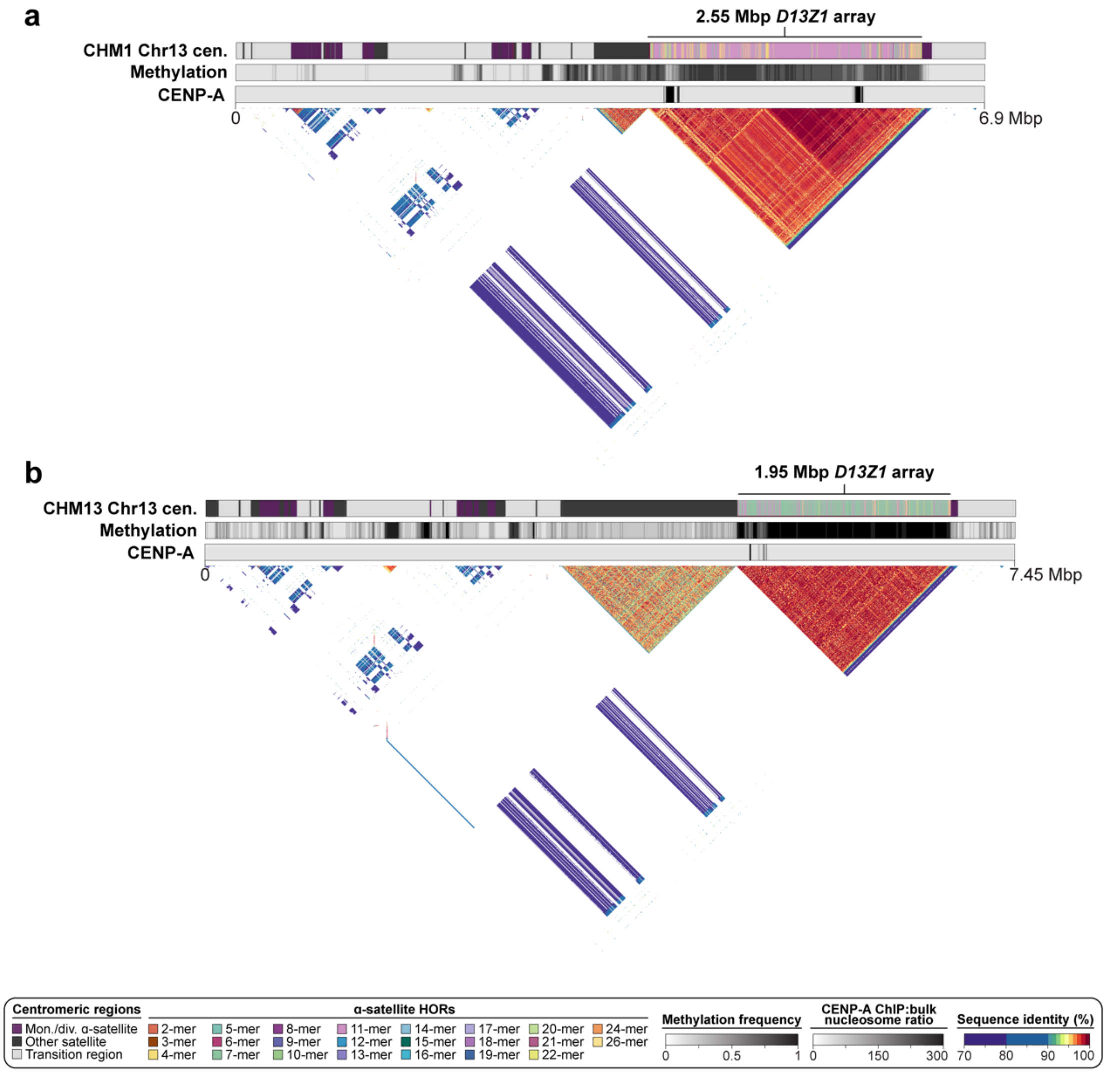


**Supplementary Figure 57. Comparison of the genetic, epigenetic, and evolutionary landscapes between the CHM1 and CHM13 chromosome 13 centromeric regions.** Plots showing the sequence organization (top track), CpG methylation frequency (second track), CENP-A nucleosome enrichment (third track), and evolutionary layers (bottom triangle) for the **a)** CHM1 and **b)** CHM13 chromosome 13 centromeric regions.

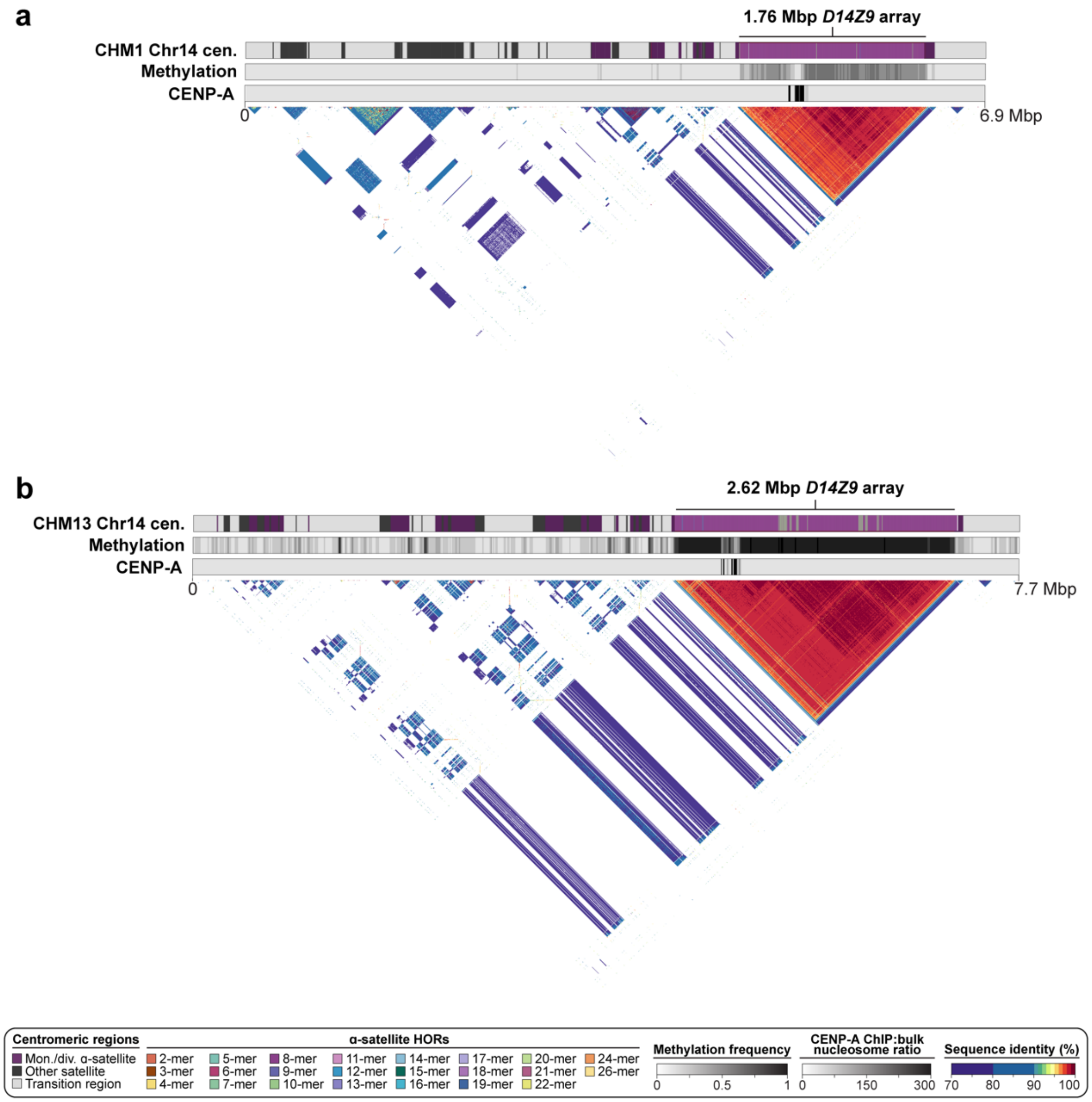


**Supplementary Figure 58. Comparison of the genetic, epigenetic, and evolutionary landscapes between the CHM1 and CHM13 chromosome 14 centromeric regions.** Plots showing the sequence organization (top track), CpG methylation frequency (second track), CENP-A nucleosome enrichment (third track), and evolutionary layers (bottom triangle) for the **a)** CHM1 and **b)** CHM13 chromosome 14 centromeric regions.

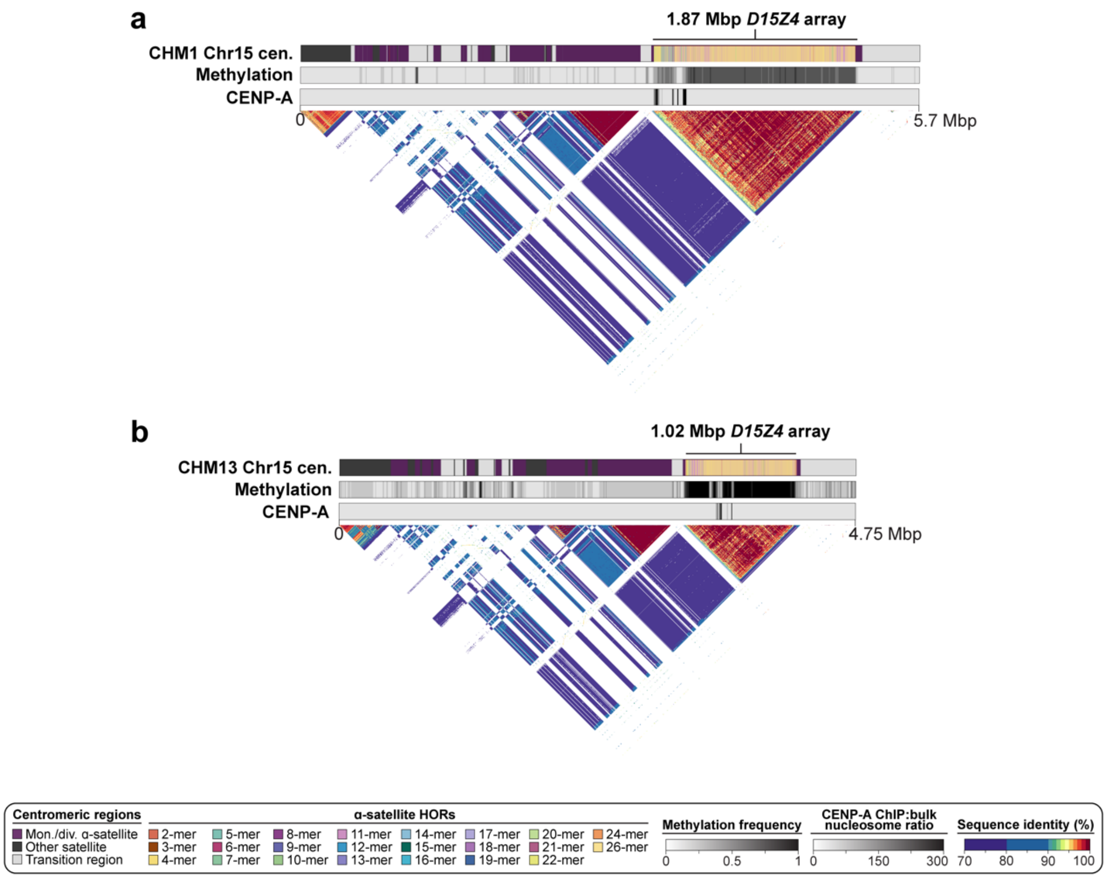


**Supplementary Figure 59. Comparison of the genetic, epigenetic, and evolutionary landscapes between the CHM1 and CHM13 chromosome 15 centromeric regions.** Plots showing the sequence organization (top track), CpG methylation frequency (second track), CENP-A nucleosome enrichment (third track), and evolutionary layers (bottom triangle) for the **a)** CHM1 and **b)** CHM13 chromosome 15 centromeric regions.

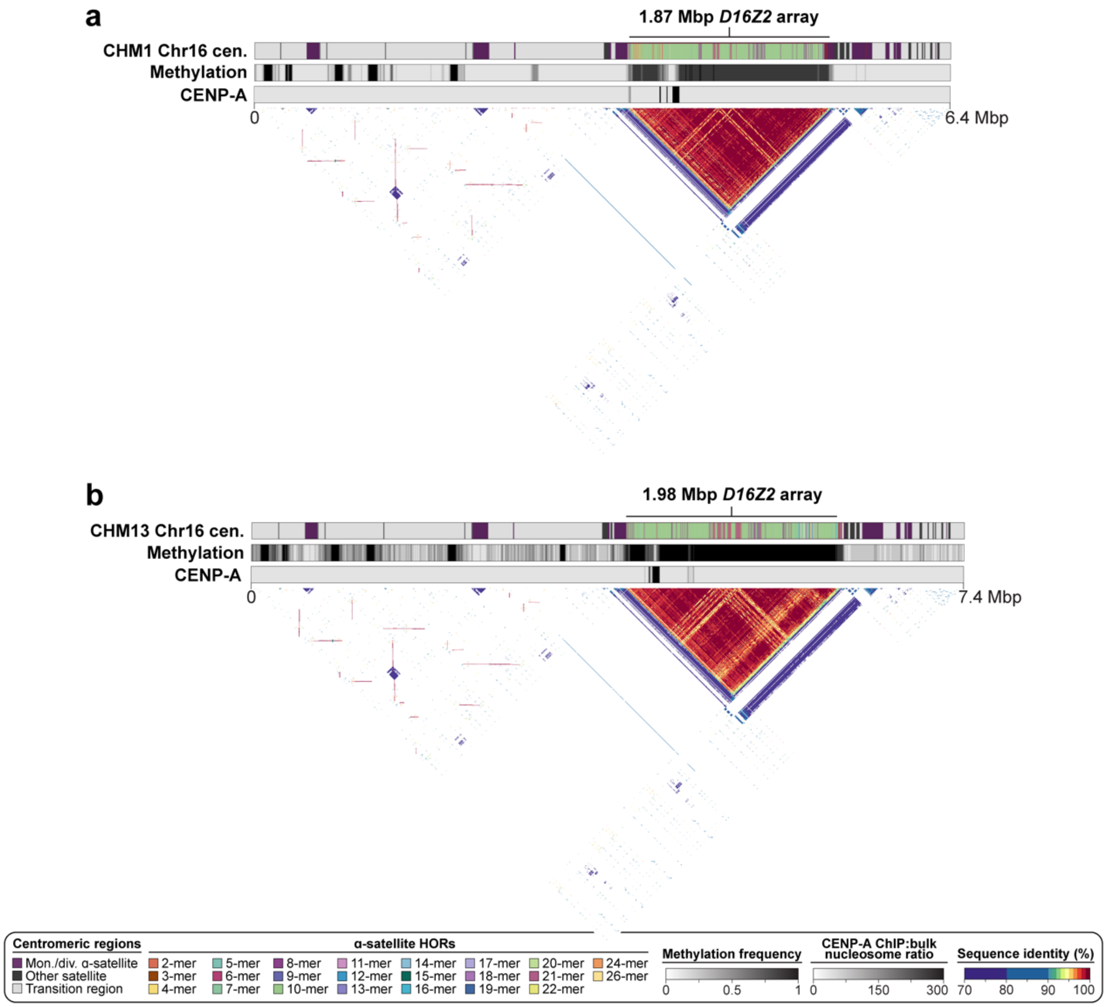


**Supplementary Figure 60. Comparison of the genetic, epigenetic, and evolutionary landscapes between the CHM1 and CHM13 chromosome 16 centromeric regions.** Plots showing the sequence organization (top track), CpG methylation frequency (second track), CENP-A nucleosome enrichment (third track), and evolutionary layers (bottom triangle) for the **a)** CHM1 and **b)** CHM13 chromosome 16 centromeric regions. We note the presence of a secondary CENP-A enrichment site that coincides with reduced CpG methylation on both CHM1 and CHM13 chromosome 16 centromeres, although the location of these sites are different.

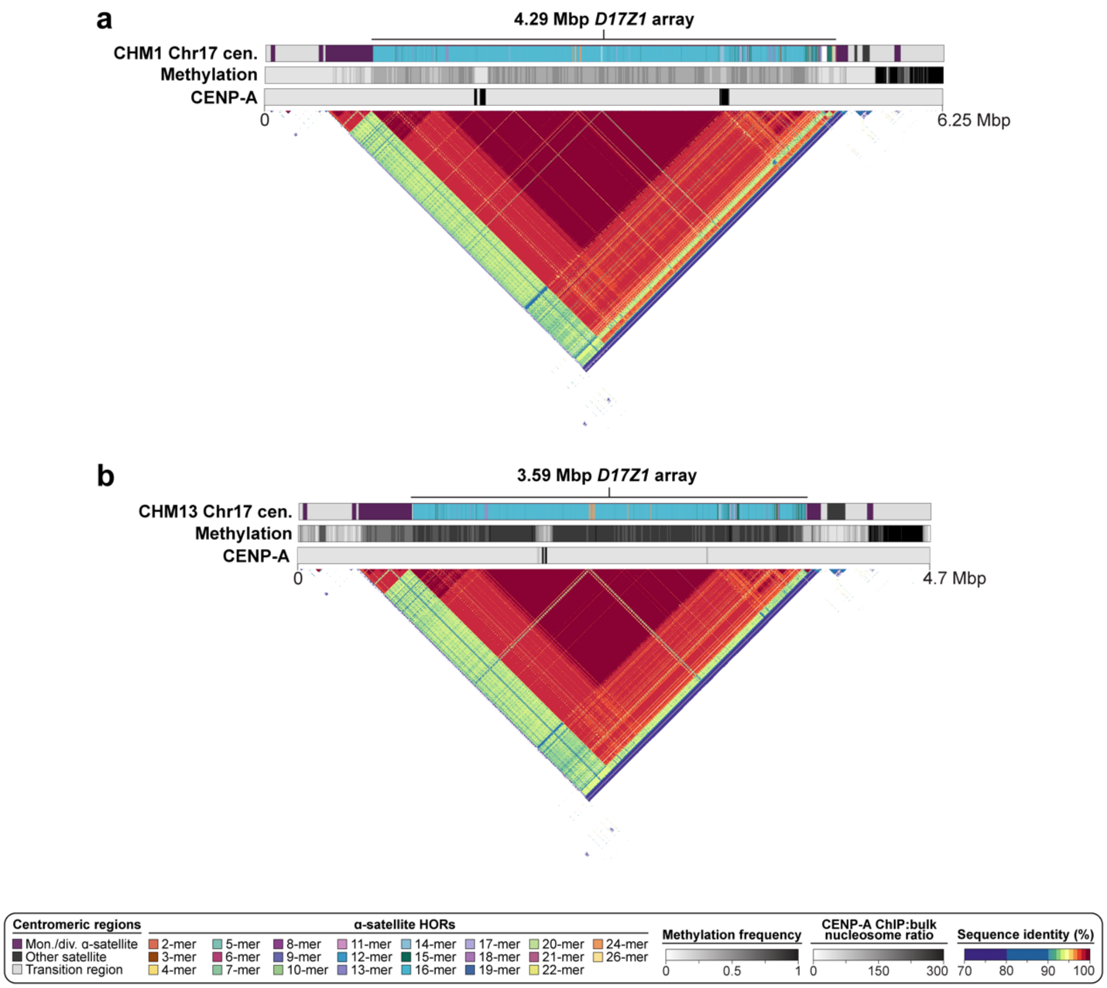


**Supplementary Figure 61. Comparison of the genetic, epigenetic, and evolutionary landscapes between the CHM1 and CHM13 chromosome 17 centromeric regions.** Plots showing the sequence organization (top track), CpG methylation frequency (second track), CENP-A nucleosome enrichment (third track), and evolutionary layers (bottom triangle) for the **a)** CHM1 and **b)** CHM13 chromosome 17 centromeric regions.

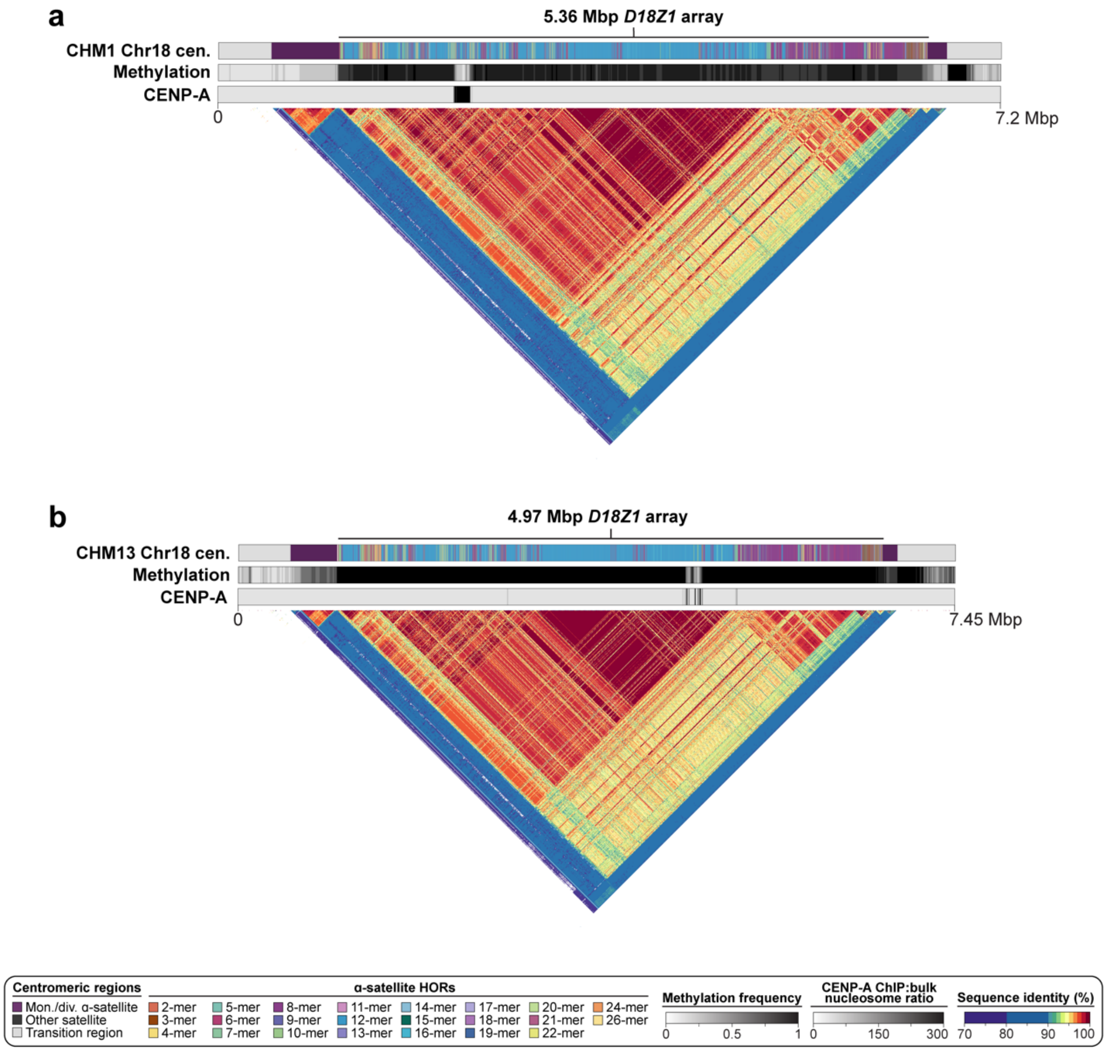


**Supplementary Figure 62. Comparison of the genetic, epigenetic, and evolutionary landscapes between the CHM1 and CHM13 chromosome 18 centromeric regions.** Plots showing the sequence organization (top track), CpG methylation frequency (second track), CENP-A nucleosome enrichment (third track), and evolutionary layers (bottom triangle) for the **a)** CHM1 and **b)** CHM13 chromosome 18 centromeric regions.

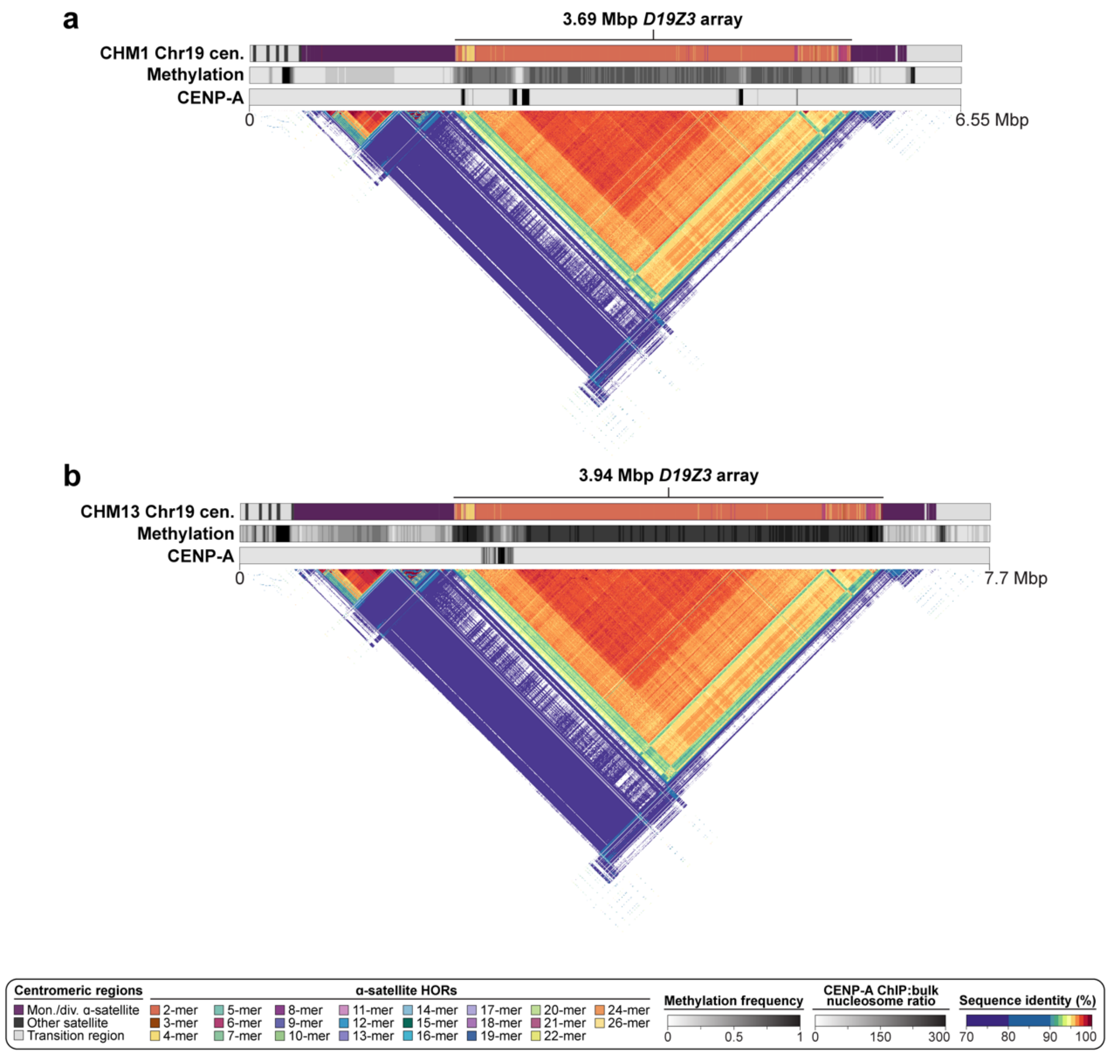


**Supplementary Figure 63. Comparison of the genetic, epigenetic, and evolutionary landscapes between the CHM1 and CHM13 chromosome 19 centromeric regions.** Plots showing the sequence organization (top track), CpG methylation frequency (second track), CENP-A nucleosome enrichment (third track), and evolutionary layers (bottom triangle) for the **a)** CHM1 and **b)** CHM13 chromosome 19 centromeric regions.

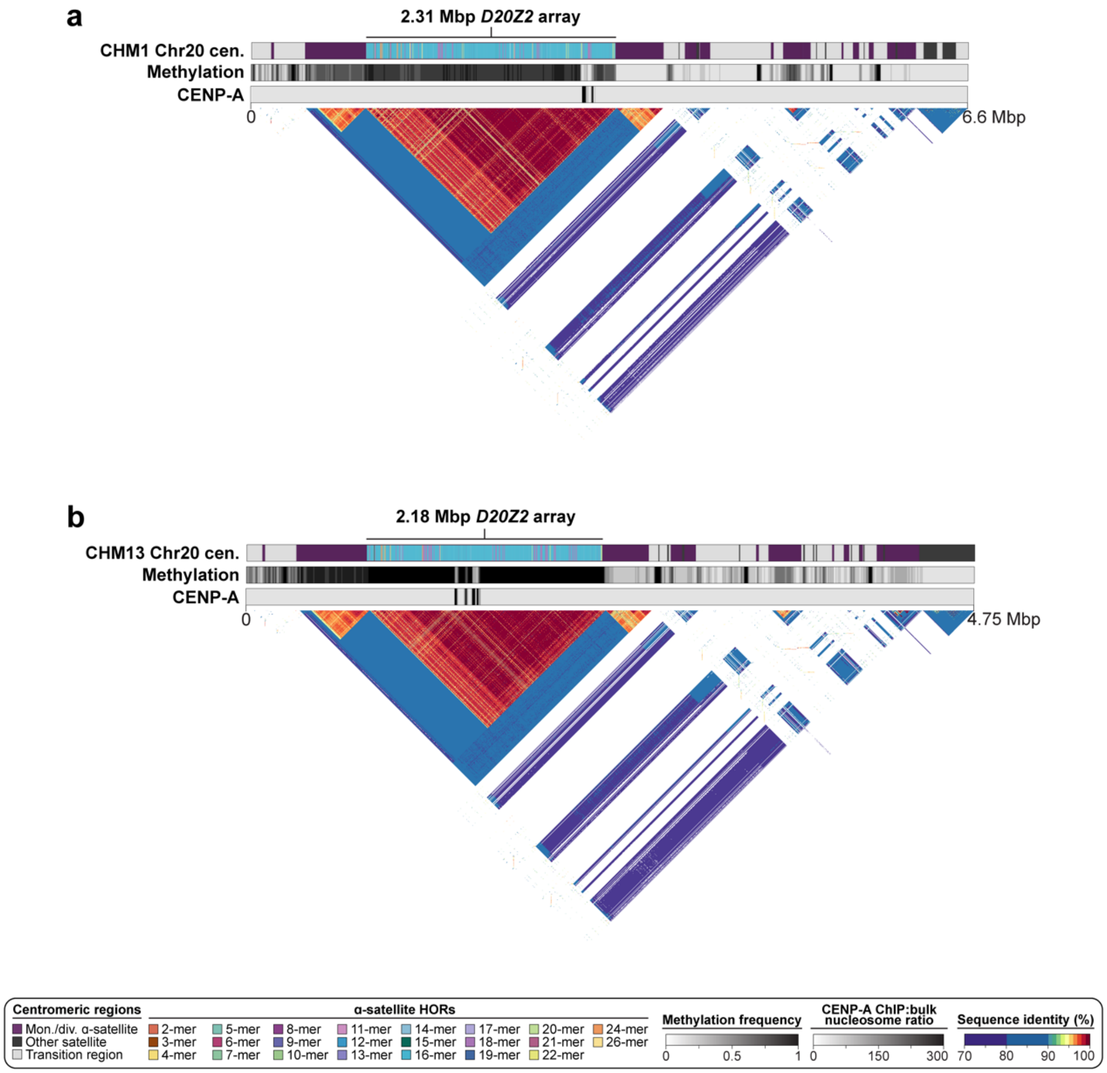


**Supplementary Figure 64. Comparison of the genetic, epigenetic, and evolutionary landscapes between the CHM1 and CHM13 chromosome 20 centromeric regions.** Plots showing the sequence organization (top track), CpG methylation frequency (second track), CENP-A nucleosome enrichment (third track), and evolutionary layers (bottom triangle) for the **a)** CHM1 and **b)** CHM13 chromosome 20 centromeric regions.

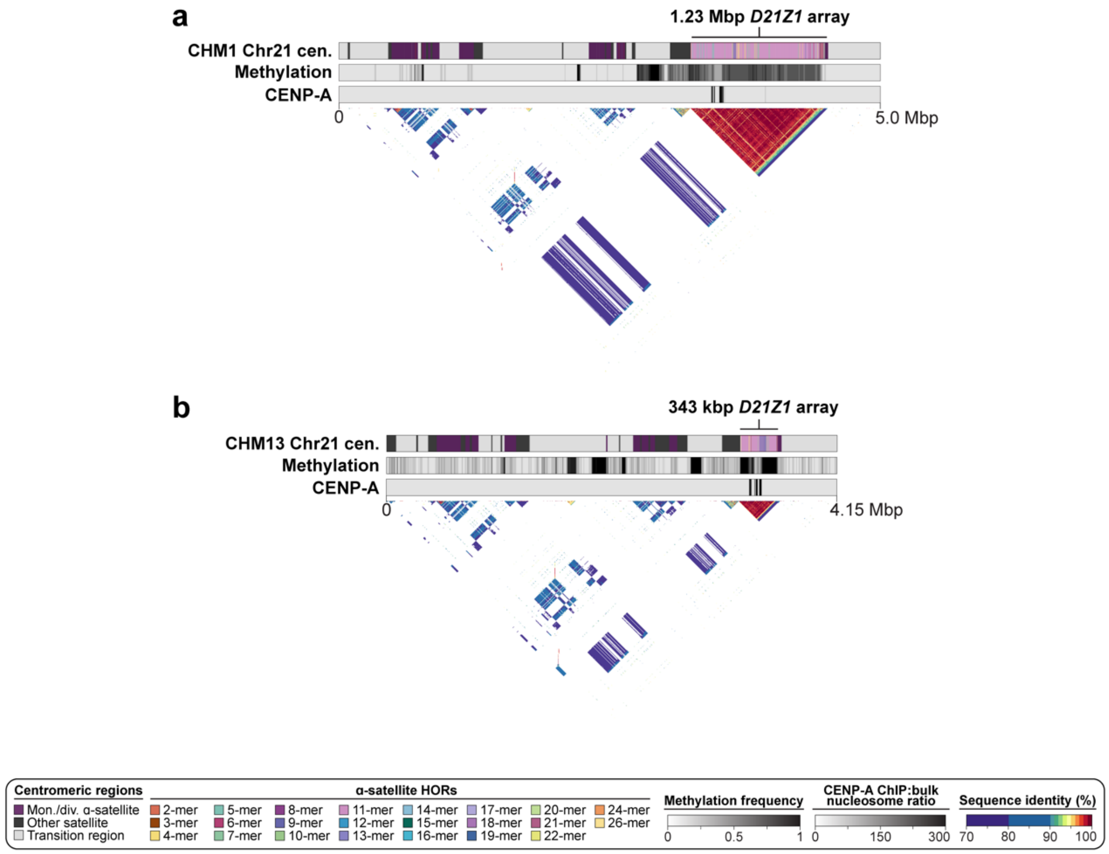


**Supplementary Figure 65. Comparison of the genetic, epigenetic, and evolutionary landscapes between the CHM1 and CHM13 chromosome 21 centromeric regions.** Plots showing the sequence organization (top track), CpG methylation frequency (second track), CENP-A nucleosome enrichment (third track), and evolutionary layers (bottom triangle) for the **a)** CHM1 and **b)** CHM13 chromosome 21 centromeric regions.

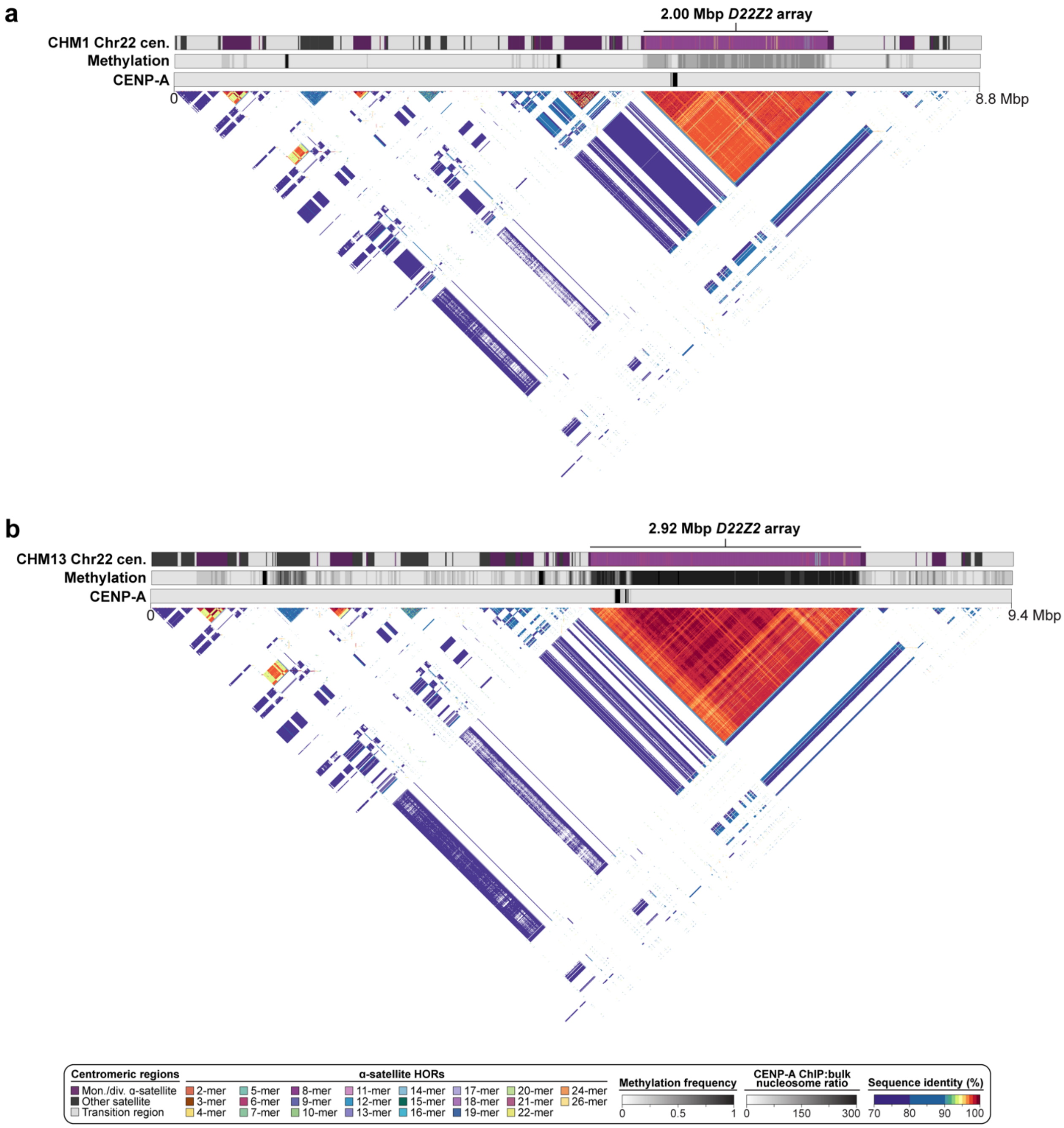


**Supplementary Figure 66. Comparison of the genetic, epigenetic, and evolutionary landscapes between the CHM1 and CHM13 chromosome 22 centromeric regions.** Plots showing the sequence organization (top track), CpG methylation frequency (second track), CENP-A nucleosome enrichment (third track), and evolutionary layers (bottom triangle) for the **a)** CHM1 and **b)** CHM13 chromosome 22 centromeric regions.

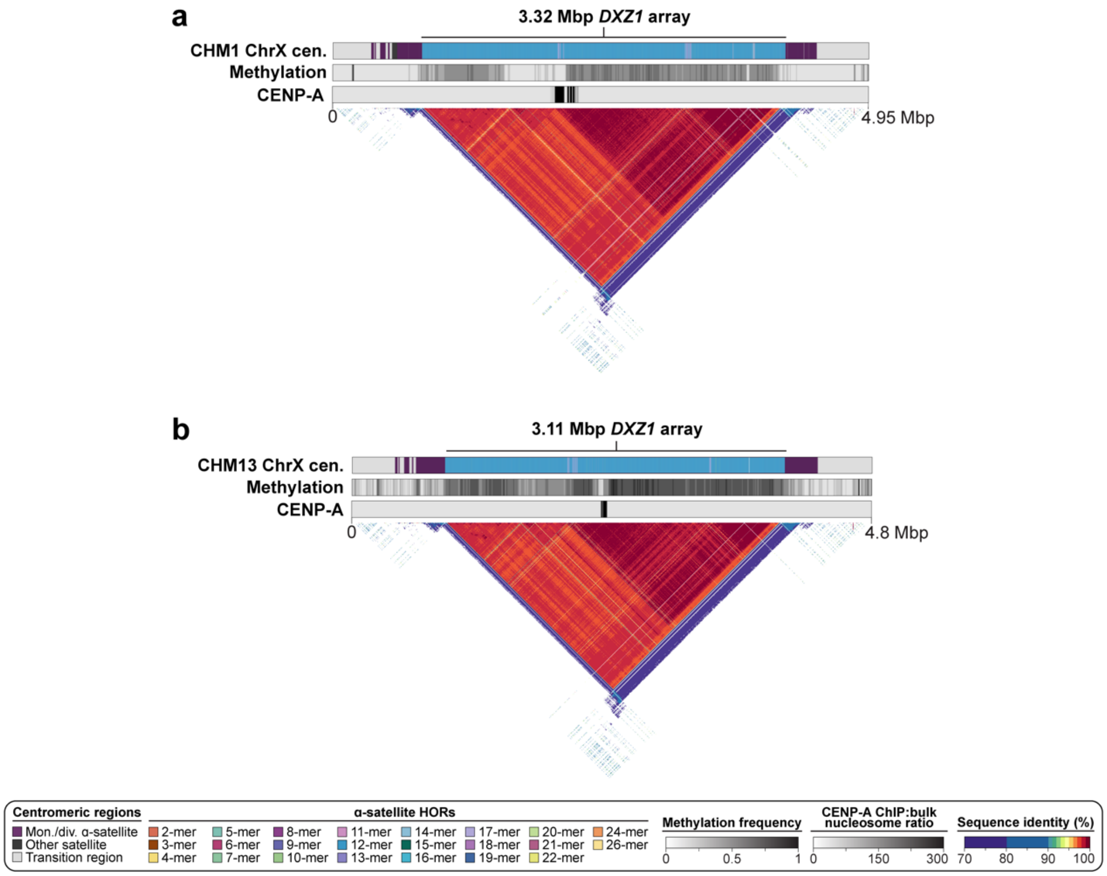


**Supplementary Figure 67. Comparison of the genetic, epigenetic, and evolutionary landscapes between the CHM1 and CHM13 chromosome X centromeric regions.** Plots showing the sequence organization (top track), CpG methylation frequency (second track), CENP-A nucleosome enrichment (third track), and evolutionary layers (bottom triangle) for the **a)** CHM1 and **b)** CHM13 chromosome X centromeric regions.

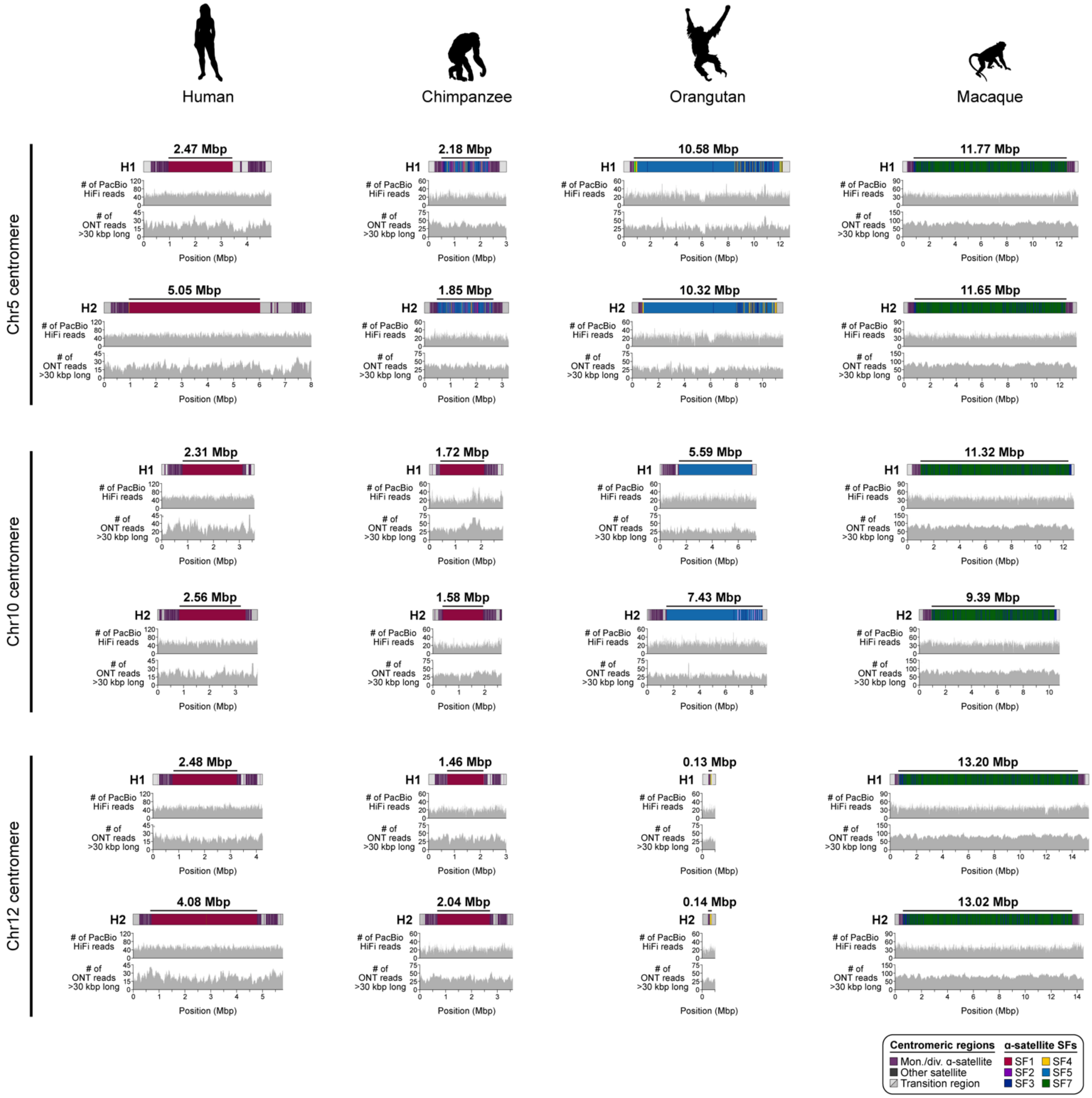


**Supplementary Figure 68. Read-depth profiles of the chromosome 5, 10, and 12 centromeric regions from the human, chimpanzee, orangutan, and macaque genomes.** Alignment of PacBio HiFi and ONT long-read sequencing data to the centromere assemblies from diverse primate species shows uniform read depth, indicating a lack of large structural errors. The human genome is HG00733. Read-depth histograms of these plots are shown in **Supplementary Figs. 70,71**.

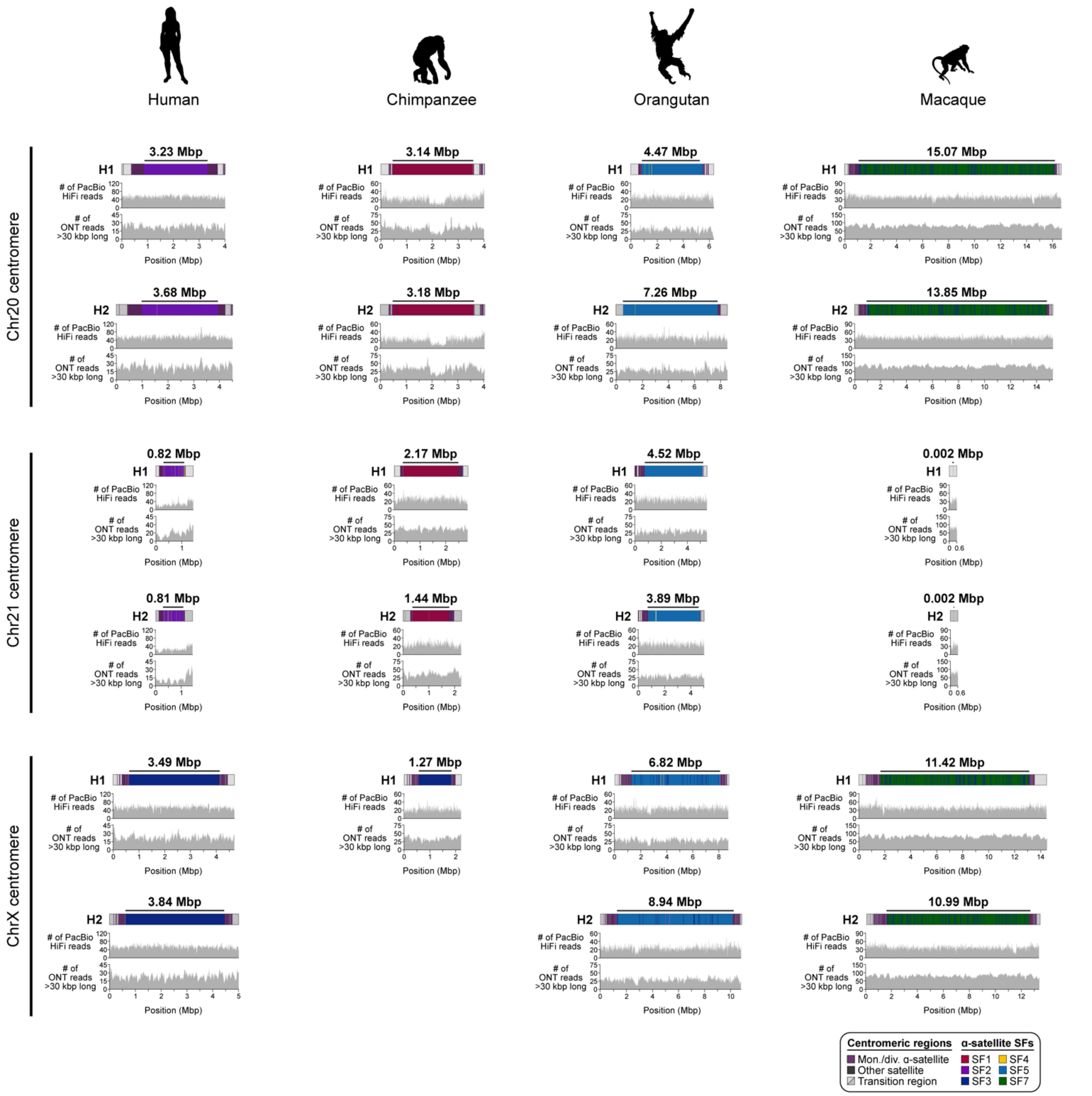


**Supplementary Figure 69. Read-depth profiles of the chromosome 20, 21, and X centromeric regions from the human, chimpanzee, orangutan, and macaque genomes.** Alignment of PacBio HiFi and ONT long-read sequencing data to the centromere assemblies from diverse primate species shows uniform read depth, indicating a lack of large structural errors. The human genome is HG00733. Read-depth histograms of these plots are shown in **Supplementary Figs. 70,71**.

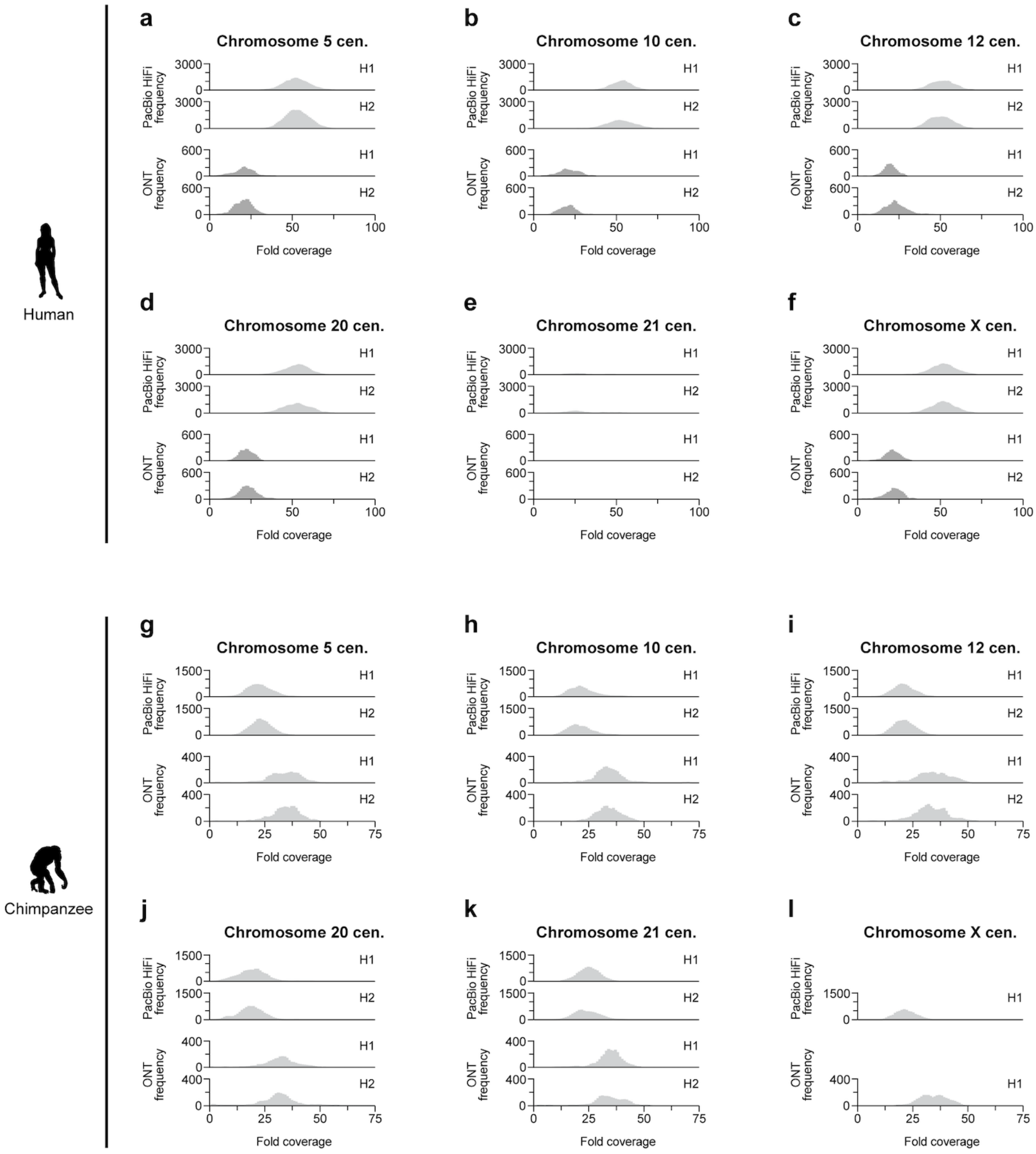


**Supplementary Figure 70. PacBio HiFi and ONT read-depth histograms for human and chimpanzee centromeres from chromosomes 5, 10, 12, 20, 21, and X. a-l)** Histograms of the PacBio HiFi (top) and ONT (bottom) read depths across the human (HG00733) chromosome **a)** 5, **b)** 10, **c)** 12, **d)** 20, **e)** 21, and **f)** X centromeres and the chimpanzee chromosome **g)** 5, **h)** 10, **i)** 12, **j)** 20, **k)** 21, and **l)** X centromeres. All read-depth distributions are consistent with Poisson sampling, with no significant outliers. We note that the human chromosome 21 centromere has lower coverage due to a smaller region being assessed as a result of a smaller α-satellite HOR array.

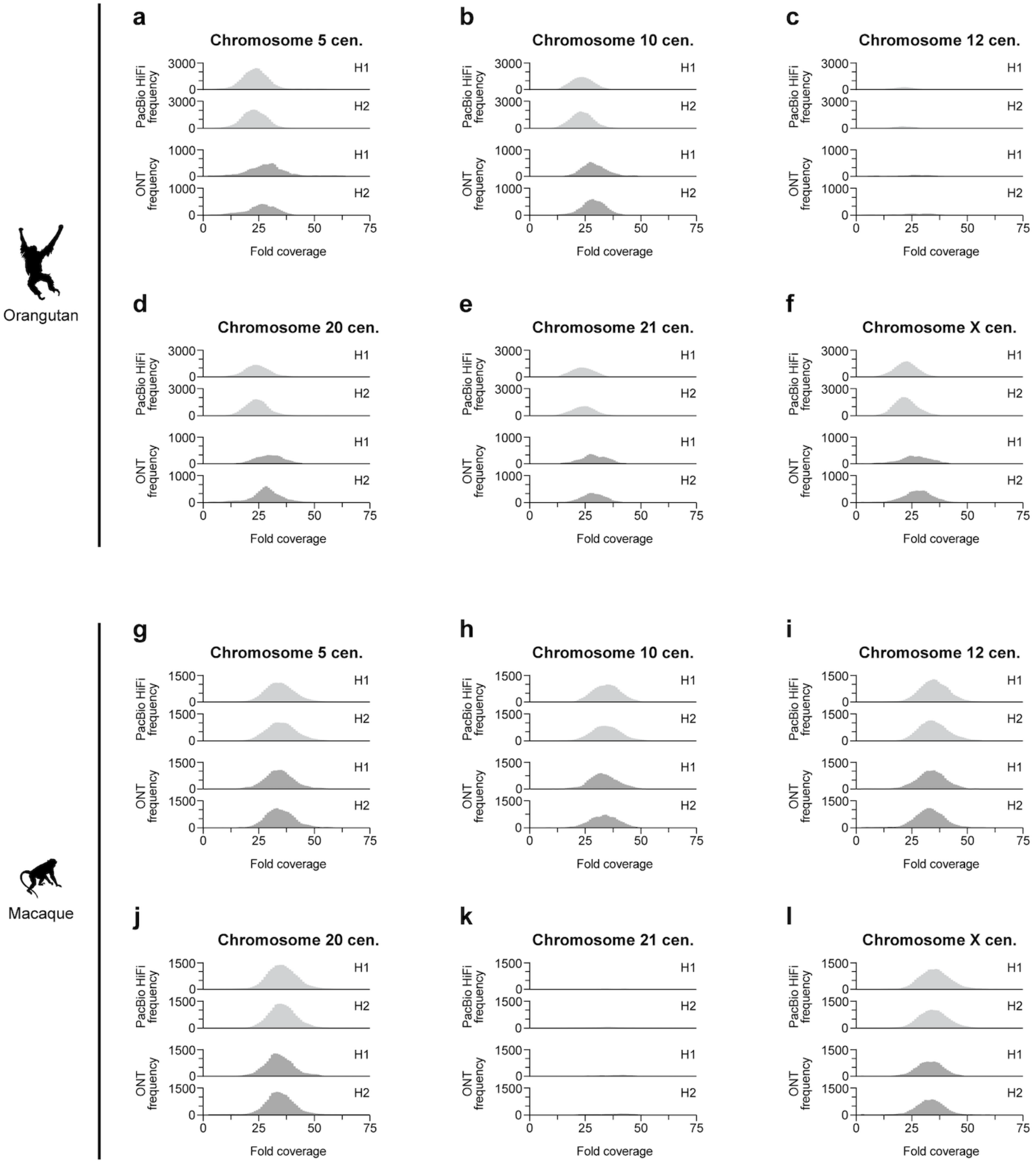


**Supplementary Figure 71. PacBio HiFi and ONT read-depth histograms for orangutan and macaque centromeres from chromosomes 5, 10, 12, 20, 21, and X. a-l)** Histograms of the PacBio HiFi (top) and ONT (bottom) read depths across the orangutan chromosome **a)** 5, **b)** 10, **c)** 12, **d)** 20, **e)** 21, and **f)** X centromeres and the macaque chromosome **g)** 5, **h)** 10, **i)** 12, **j)** 20, **k)** 21, and **l)** X centromeres. All read-depth distributions are consistent with Poisson sampling, with no significant outliers. We note that the orangutan chromosome 12 centromere and macaque chromosome 21 centromere have lower coverage due to inactivation of that centromere and, consequently, a smaller region being assessed.

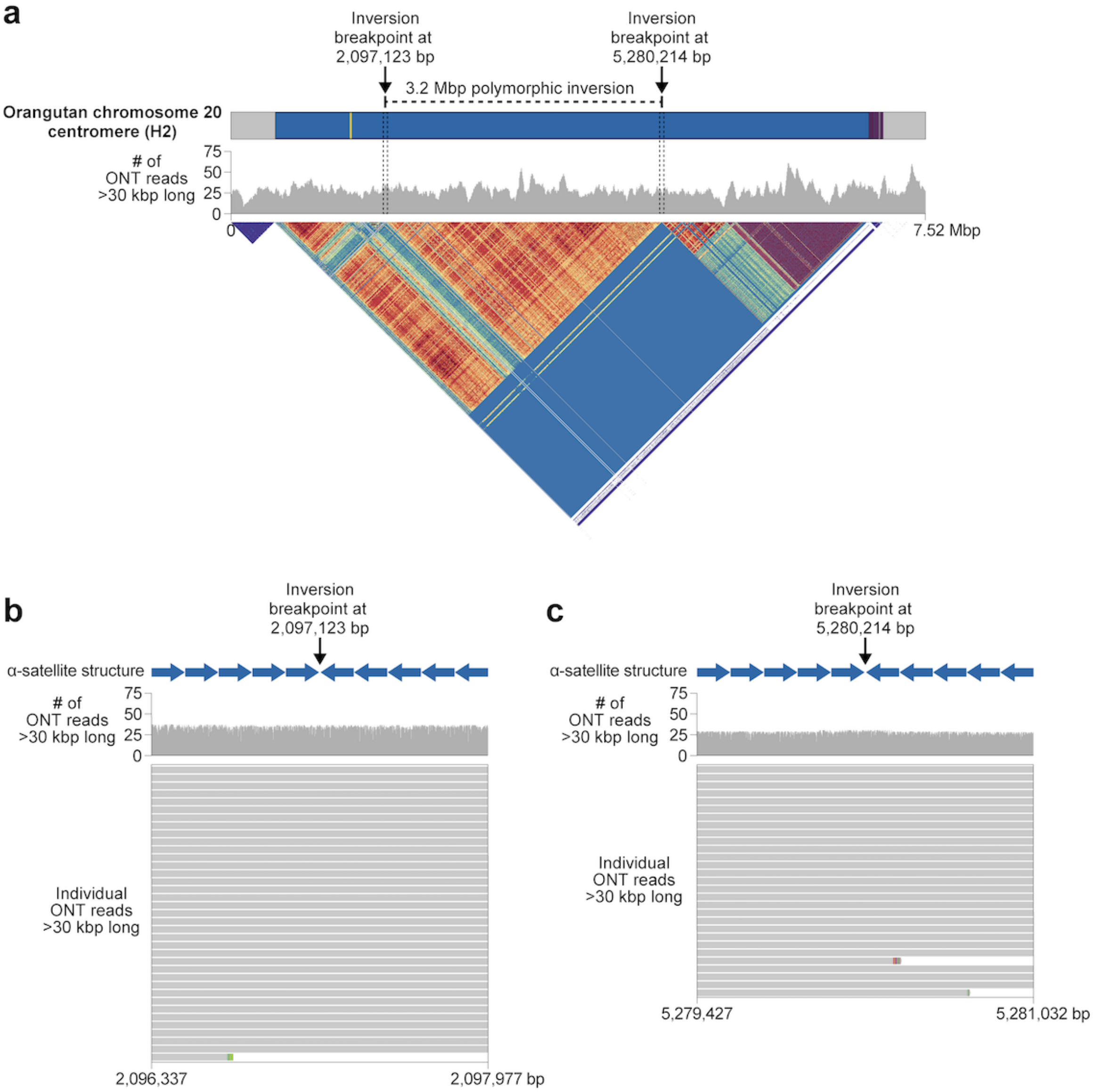


**Supplementary Figure 72. Detection of a 3.2 Mbp polymorphic inversion in the orangutan chromosome 20 centromere. a)** Location of a 3.2 Mbp inversion in the orangutan chromosome 20 centromeric α-satellite HOR array in haplotype 2 (H2). This inversion is located at bases 2,097,123-5,280,214 and was detected with both HumAS-HMMER (https://github.com/fedorrik/HumAS-HMMER_for_AnVIL) and StringDecomposer[20]. **b,c)** Uniform coverage of orangutan ONT reads >30 kbp long across the **b)** upstream and **c)** downstream inversion breakpoints supports this structural variant.

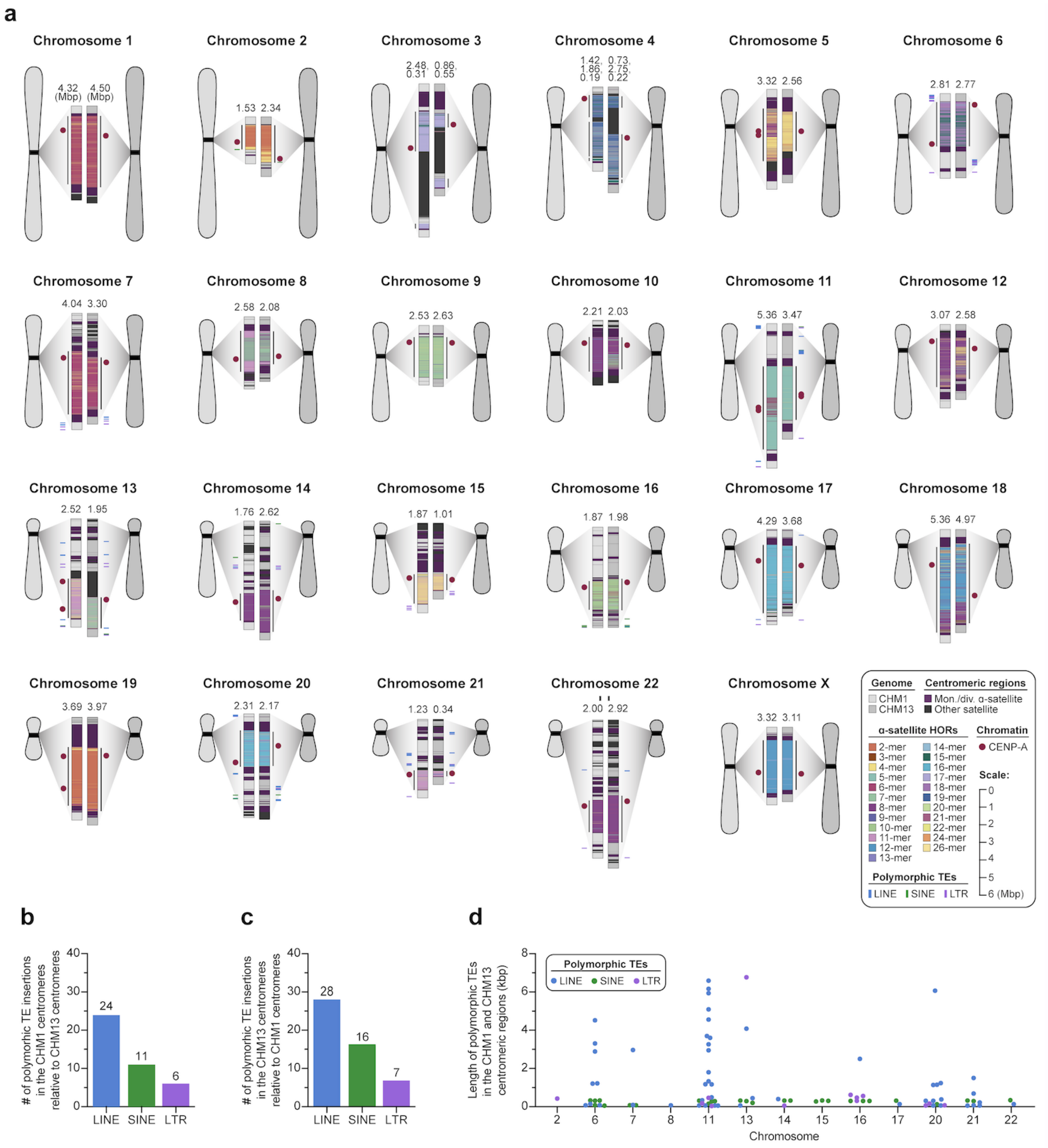

**Supplementary Figure 73. Polymorphic TEs within the CHM1 and CHM13 centromeric regions. a)** Map of the CHM1 and CHM13 centromeric regions, showing the location of 92 total LINEs (blue), SINEs (green), and LTRs (purple) relative to the α-satellite HOR array(s) and kinetochore(s). The TEs are shown as colorful lines next to the centromeric structures. **b,c)** Number of polymorphic LINE, SINE, and LTR insertions for the **b)** CHM1 centromeric regions and **c)** CHM13 centromeric regions. **d)** Length of the polymorphic TEs in the CHM1 and CHM13 centromeric regions.

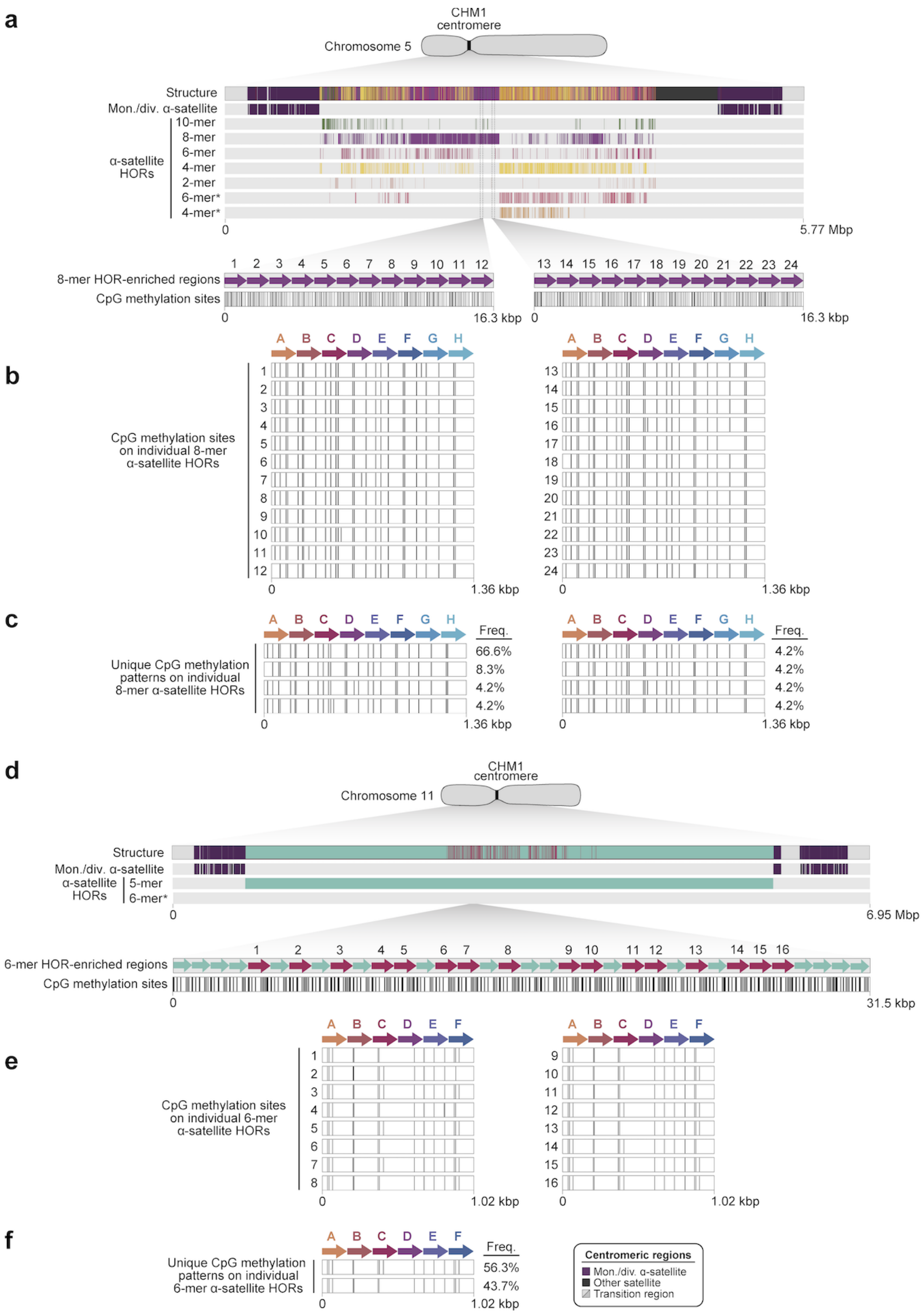


**Supplementary Figure 74. Expanded α-satellite HORs in the CHM1 chromosome 5 and 11 centromeres have divergent CpG methylation patterns. a,d)** CpG methylation patterns on recently expanded α-satellite HORs in the core of the CHM1 **a)** chromosome 5 centromere and **d)** chromosome 11 centromere. **b,e)** CpG methylation patterns on individual α-satellite monomers from the HORs within the core of the CHM1 **b)** chromosome 5 centromere or **e)** chromosome 11 centromere. **c,f)** Unique CpG methylation patterns and their frequencies within the recently expanded α-satellite HORs within the CHM1 **c)** chromosome 5 centromere and **f)** chromosome 11 centromere.

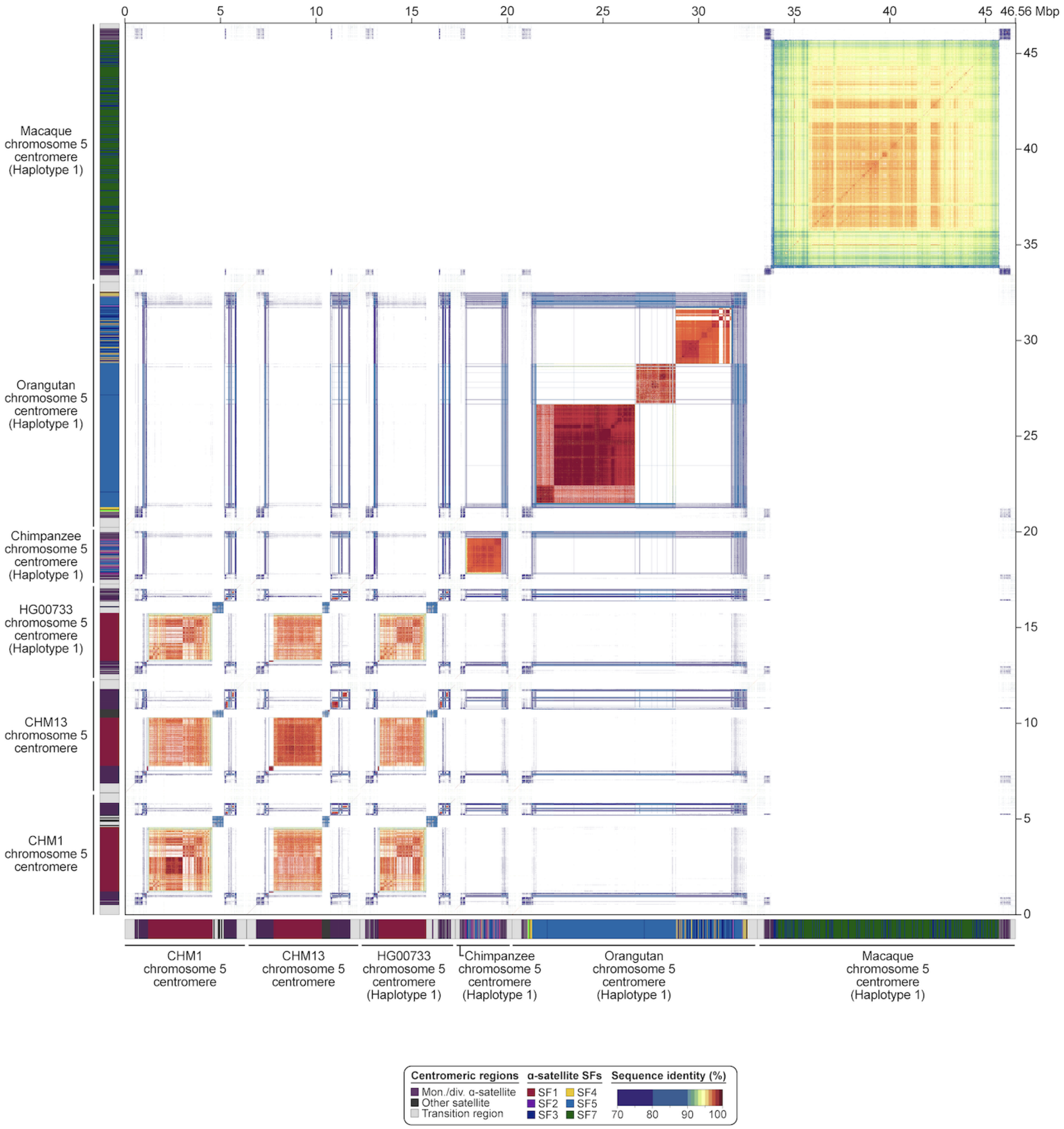


**Supplementary Figure 75. Sequence identity map of the chromosome 5 centromeres from six human and NHPs.** A sequence identity map of the chromosome 5 centromeres from CHM1, CHM13, human (HG00733), chimpanzee, orangutan, and macaque genomes (generated via StainedGlass[17]) reveal 70-90% sequence identity among monomeric/diverged α-satellite flanking the α-satellite HOR array.

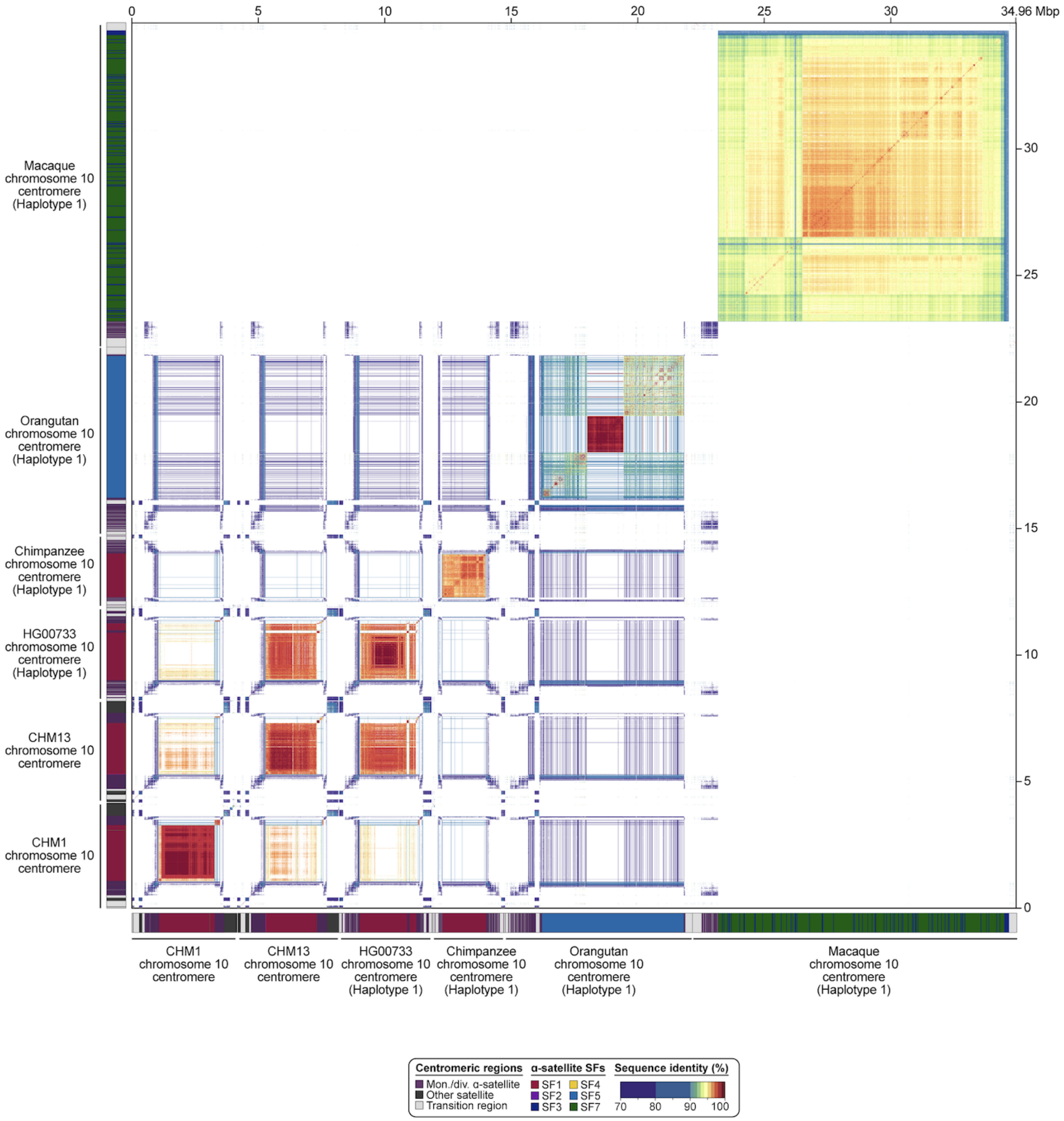


**Supplementary Figure 76. Sequence identity map of the chromosome 10 centromeres from six human and NHPs.** A sequence identity map of the chromosome 10 centromeres from CHM1, CHM13, human (HG00733), chimpanzee, orangutan, and macaque genomes (generated via StainedGlass[17]) reveal 70-90% sequence identity among monomeric/diverged α-satellite flanking the α-satellite HOR array.

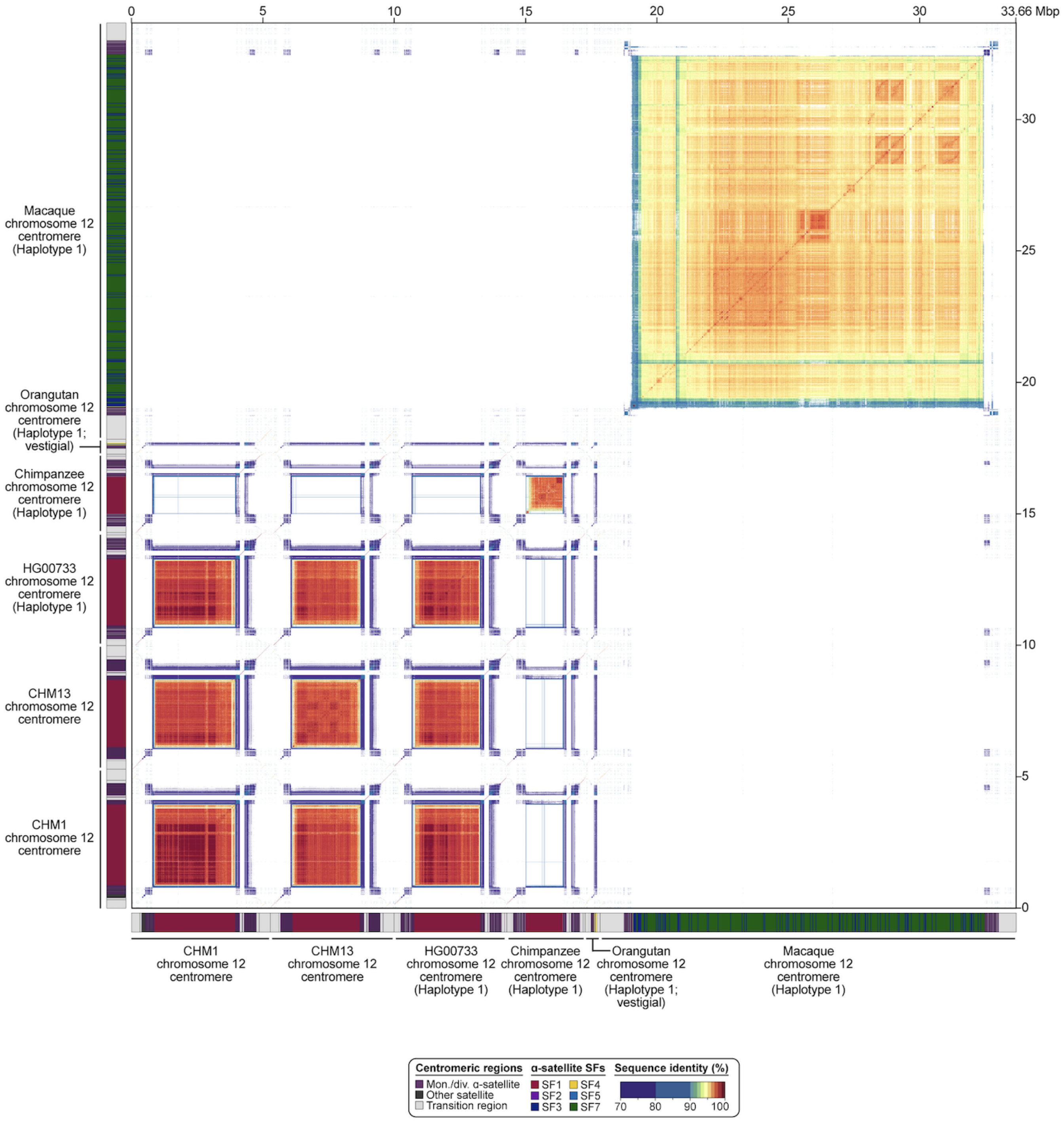


**Supplementary Figure 77. Sequence identity map of the chromosome 12 centromeres from six human and NHPs.** A sequence identity map of the chromosome 12 centromeres from CHM1, CHM13, human (HG00733), chimpanzee, orangutan, and macaque genomes (generated via StainedGlass[17]) reveal 70-90% sequence identity among monomeric/diverged α-satellite flanking the α-satellite HOR array.

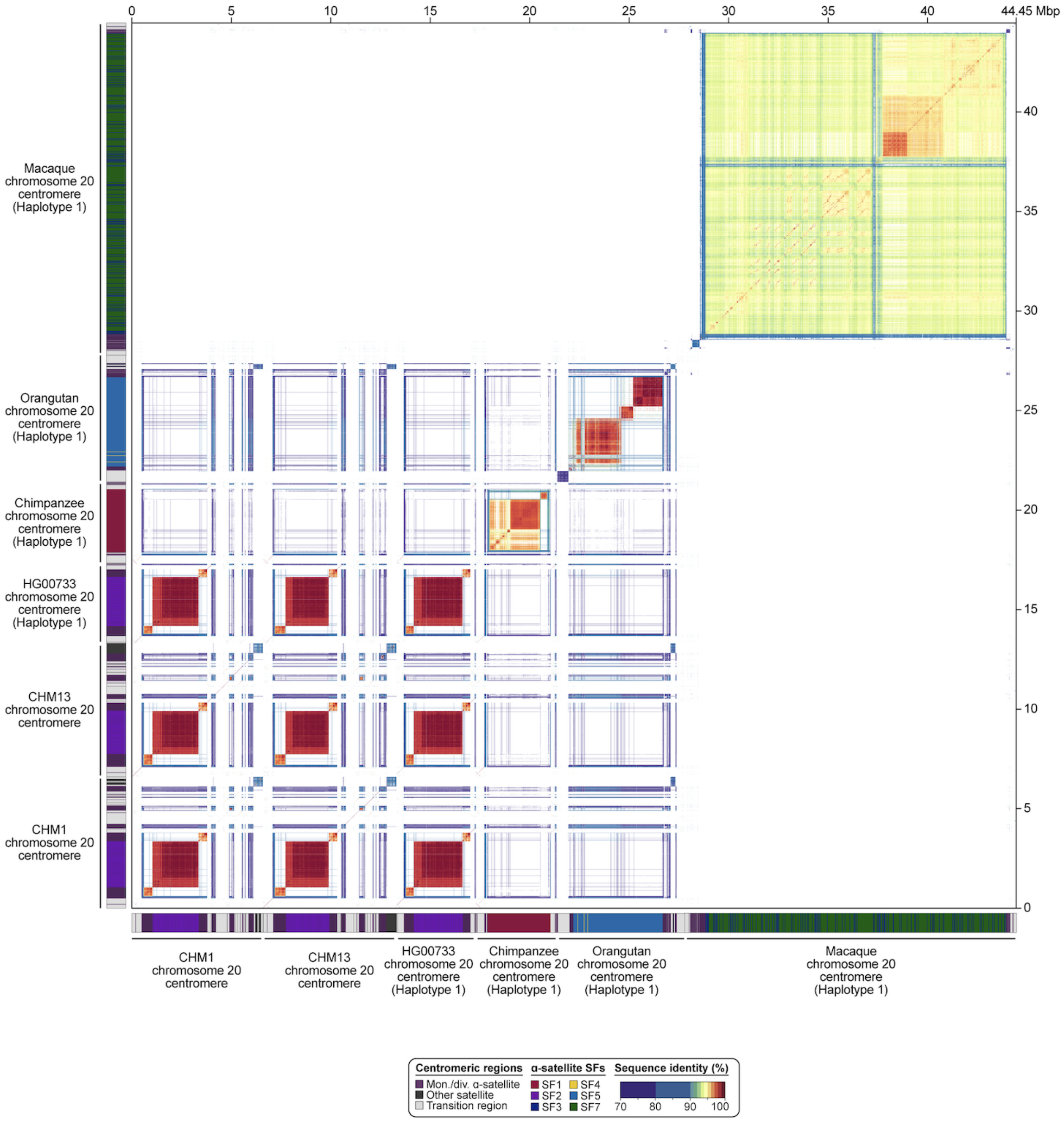


**Supplementary Figure 78. Sequence identity map of the chromosome 20 centromeres from six human and NHPs.** A sequence identity map of the chromosome 20 centromeres from CHM1, CHM13, human (HG00733), chimpanzee, orangutan, and macaque genomes (generated via StainedGlass[17]) reveal 70-90% sequence identity among monomeric/diverged α-satellite flanking the α-satellite HOR array as well as some α-satellite HORs within the array.

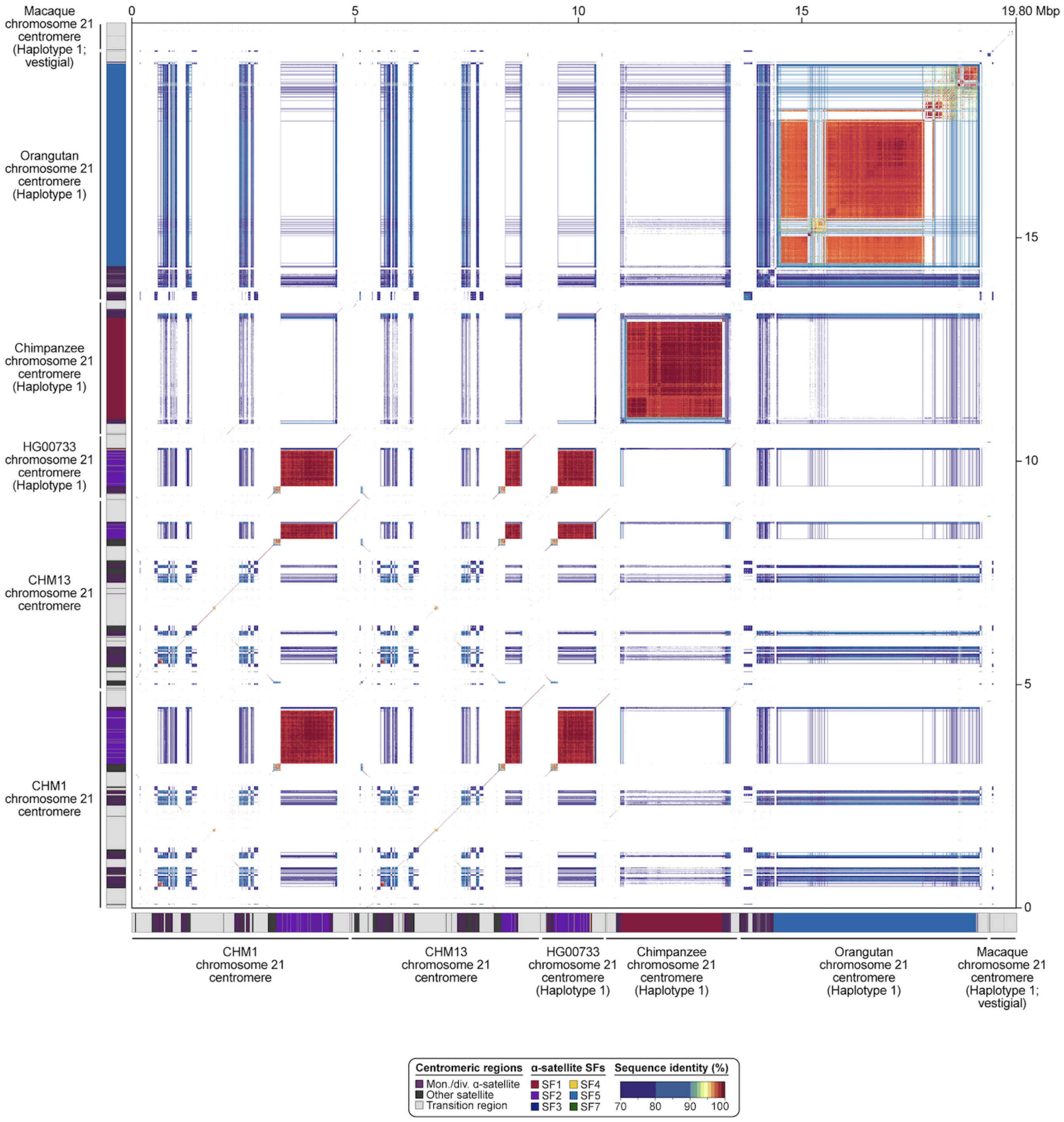


**Supplementary Figure 79. Sequence identity map of the chromosome 21 centromeres from six human and NHPs.** A sequence identity map of the chromosome 21 centromeres from CHM1, CHM13, human (HG00733), chimpanzee, orangutan, and macaque genomes (generated via StainedGlass[17]) reveal 70-90% sequence identity among monomeric/diverged α-satellite flanking the α-satellite HOR array.

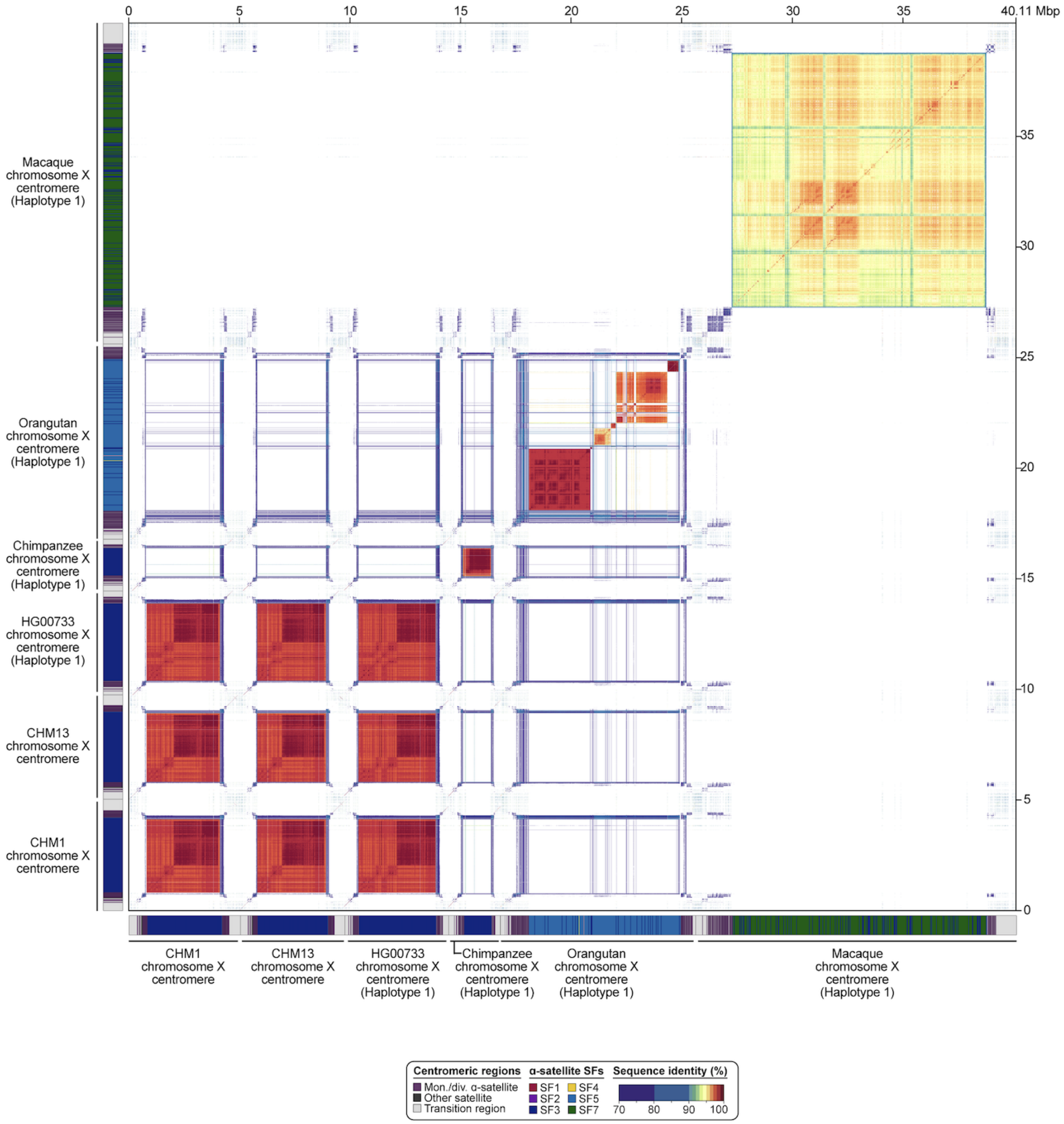


**Supplementary Figure 80. Sequence identity map of the chromosome X centromeres from six human and NHPs.** A sequence identity map of the chromosome X centromeres from CHM1, CHM13, human (HG00733), chimpanzee, orangutan, and macaque genomes (generated via StainedGlass[17]) reveal 70-90% sequence identity among monomeric/diverged α-satellite flanking the α-satellite HOR array.

## SUPPLEMENTARY TABLES

**Supplementary Table 1. Statistics of long-read sequencing datasets and genome assemblies.** See accompanying Excel file.

**Supplementary Table 2. Support for CHM1 and CHM13 centromere assemblies from 56 diverse human genomes sequenced by the HPRC and HGSVC.** See accompanying Excel file.

**Supplementary Table 3. Three alignment strategies for assessing centromere sequence identity.** See accompanying Excel file.

**Supplementary Table 4. Sequence identity calculated from full contig alignments between CHM1 and CHM13 centromeres.** See accompanying Excel file.

**Supplementary Table 5. Sequence identity calculated from alignments of 10-kbp segments between the CHM1 and CHM13 centromeres.** See accompanying Excel file.

**Supplementary Table 6. Sequence identity and alignment statistics of centromeric α-satellite HOR arrays from CHM1, CHM13, and 56 diverse human genomes.** See accompanying Excel file.

**Supplementary Table 7. Quantification of the genetic and epigenetic variation of all CHM1 and CHM13 centromeres.** See accompanying Excel file.

**Supplementary Table 8. Catalog of all α-satellite HOR variants in the CHM1 and CHM13 centromeres.** See accompanying Excel file.

**Supplementary Table 9. Quantification of changes in bases, α-satellite monomers, and α-satellite HORs among centromeric arrays with the same monophyletic origin.** See accompanying Excel file.

**Supplementary Table 10. SNP density and Ti/Tv ratios for 70 monomeric/diverged α-satellite regions across the CHM13 genome.** See accompanying Excel file.

**Supplementary Table 11. SNP density and Ti/Tv ratios for 500 unique regions across the CHM13 genome.** See accompanying Excel file.

## SUPPLEMENTARY INFORMATION REFERENCES


1. Vollger, M. R. *et al.* Increased mutation and gene conversion within human segmental duplications. *Nature* **617**, 325–334 (2023).
2. Nurk, S. *et al.* The complete sequence of a human genome. *Science* **376**, 44–53 (2022).
3. Porter, S. *et al.* ADAMTS8 and ADAMTS15 expression predicts survival in human breast carcinoma. *International Journal of Cancer* **118**, 1241–1247 (2006).
4. Viloria, C. G. *et al.* Genetic Inactivation of ADAMTS15 Metalloprotease in Human Colorectal Cancer. *Cancer Research* **69**, 4926–4934 (2009).
5. Toyooka, K. O. *et al.* Loss of expression and aberrant methylation of the CDH13 (H-cadherin) gene in breast and lung carcinomas. *Cancer Res* **61**, 4556–4560 (2001).
6. Sato, M., Mori, Y., Sakurada, A., Fujimura, S. & Horii, A. The H-cadherin (CDH13) gene is inactivated in human lung cancer. *Hum Genet* **103**, 96–101 (1998).
7. Zhu, Q. *et al.* Hypomethylation of RPTOR in peripheral blood is associated with very early-stage lung cancer. *Clin Chim Acta* **537**, 173–180 (2022).
8. Lu, X. *et al.* Meta-analysis of the association between mTORC1-related genes polymorphisms and cancer risk. *Pathol Res Pract* **229**, 153696 (2022).
9. Cheng, H., Concepcion, G. T., Feng, X., Zhang, H. & Li, H. Haplotype-resolved de novo assembly using phased assembly graphs with hifiasm. *Nature Methods* **18**, 170–175 (2021).
10. Nurk, S. *et al.* HiCanu: accurate assembly of segmental duplications, satellites, and allelic variants from high-fidelity long reads. *Genome Res.* gr.263566.120 (2020) doi:10.1101/gr.263566.120.
11. Rhie, A., Walenz, B. P., Koren, S. & Phillippy, A. M. Merqury: reference-free quality, completeness, and phasing assessment for genome assemblies. *Genome Biology* **21**, 245 (2020).
12. Nurk, S. *et al.* The complete sequence of a human genome. *Science* **376**, 44–53 (2022).
13. Falconer, E. *et al.* DNA template strand sequencing of single-cells maps genomic rearrangements at high resolution. *Nat. Methods* **9**, 1107–1112 (2012).
14. Altemose, N. *et al.* Complete genomic and epigenetic maps of human centromeres. *Science* **376**, eabl4178 (2022).

15. Dishuck, P. C., Rozanski, A. N., Logsdon, G. A., Porubsky, D. & Eichler, E. E. GAVISUNK: genome assembly validation via inter-SUNK distances in Oxford Nanopore reads. *Bioinformatics* btac714 (2022) doi:10.1093/bioinformatics/btac714.
16. Rautiainen, M. *et al.* Telomere-to-telomere assembly of diploid chromosomes with Verkko. *Nat Biotechnol* 1–9 (2023) doi:10.1038/s41587-023-01662-6.
17. Vollger, M. R., Kerpedjiev, P., Phillippy, A. M. & Eichler, E. E. StainedGlass: interactive visualization of massive tandem repeat structures with identity heatmaps. *Bioinformatics* **38**, 2049–2051 (2022).
18. Liao, W.-W. *et al.* A draft human pangenome reference. *Nature* **617**, 312–324 (2023).
19. Ebert, P. *et al.* Haplotype-resolved diverse human genomes and integrated analysis of structural variation. *Science* (2021) doi:10.1126/science.abf7117.
20. Dvorkina, T., Bzikadze, A. V. & Pevzner, P. A. The string decomposition problem and its applications to centromere analysis and assembly. *Bioinformatics* **36**, i93–i101 (2020).
