# Supplementary material for: The variation and evolution of complete human centromeres
Source: Nature. 2024 Apr 3;629(8010):136–45. doi: 10.1038/s41586-024-07278-3 (PMC11062924; doi:10.1038/s41586-024-07278-3)
Supplement: Supplementary file 2 [file 41586_2024_7278_MOESM2_ESM.pdf]

nature portfolio

Corresponding author(s): Evan E. Eichler

Last updated by author(s): Dec 28, 2023

Reporting Summary

Nature Portfolio wishes to improve the reproducibility of the work that we publish. This form provides structure for consistency and transparency in reporting. For further information on Nature Portfolio policies, see our Editorial Policies and the Editorial Policy Checklist.

Statistics

For all statistical analyses, confirm that the following items are present in the figure legend, table legend, main text, or Methods section.

n/a	Confirmed	
☐	☒	The exact sample size (*n*) for each experimental group/condition, given as a discrete number and unit of measurement
☐	☒	A statement on whether measurements were taken from distinct samples or whether the same sample was measured repeatedly
☐	☒	The statistical test(s) used AND whether they are one- or two-sided<br>*Only common tests should be described solely by name; describe more complex techniques in the Methods section.*
☒	☐	A description of all covariates tested
☐	☒	A description of any assumptions or corrections, such as tests of normality and adjustment for multiple comparisons
☐	☒	A full description of the statistical parameters including central tendency (e.g. means) or other basic estimates (e.g. regression coefficient) AND variation (e.g. standard deviation) or associated estimates of uncertainty (e.g. confidence intervals)
☐	☒	For null hypothesis testing, the test statistic (e.g. *F*, *t*, *r*) with confidence intervals, effect sizes, degrees of freedom and *P* value noted<br>*Give P values as exact values whenever suitable.*
☒	☐	For Bayesian analysis, information on the choice of priors and Markov chain Monte Carlo settings
☒	☐	For hierarchical and complex designs, identification of the appropriate level for tests and full reporting of outcomes
☒	☐	Estimates of effect sizes (e.g. Cohen's *d*, Pearson's *r*), indicating how they were calculated

Our web collection on statistics for biologists contains articles on many of the points above.

Software and code

Policy information about availability of computer code

Data collection	The software used to collect sequencing data are Pacific Bioscience Sequel II Instrument Control SW (v7.0, 7.1, and 8.0) and Oxford Nanopore Technologies PromethION software (v21.02.17 - 23.04.5). The software used to collect image data are ZEN (v3.7) and NIS-Elements AR (v3.2).
Data analysis	Custom code for the SUNK-based sequence assembly of centromeric regions is publicly available at https://github.com/arozanski97/SUNK-based-contig-scaffolding. Custom code to detect hypomethylated regions within centromeric regions, termed "centromere dip regions” (CDRs), is publicly available at https://github.com/arozanski97/CDR-Finder. Other publicly available software used in this study include DeepConsensus (v0.2.0), PacBio circular consensus sequencing software (v3.4.1), hifiasm (v0.16.1), HiCanu (v2.1.1), minimap2 (v2.17-r941 and v2.24), Jellyfish (v2.2.4), pbmm2 (v1.1.0), Winnowmap (v1.0), Merqury (v1.1), BWA-MEM (v0.7.17), sambamba (v1.0), SAMtools (v1.9), breakpointR (v1.18), BEDtools (v2.29.0), deepTools (v3.4.3), seqtk (v1.3), TandemAligner (v0.1), meryl (v1.3), StringDecomposer (no version specified), StainedGlass (v6.7.0), Nanopolish (v0.13.3), RepeatMasker (v4.1.0), Sickle (v1.33), Cutadapt (v1.18), MAFFT (v7.453), IQ-TREE (v2.1.2), Fiji (v2.13.1), ImageJ (v1.53k), KISS ImageJ plug-in (v1), Prism (v9.5.1), and R (v1.1.383).

For manuscripts utilizing custom algorithms or software that are central to the research but not yet described in published literature, software must be made available to editors and reviewers. We strongly encourage code deposition in a community repository (e.g. GitHub). See the Nature Portfolio guidelines for submitting code & software for further information.

Data

Policy information about availability of data

All manuscripts must include a data availability statement. This statement should provide the following information, where applicable:

- Accession codes, unique identifiers, or web links for publicly available datasets
- A description of any restrictions on data availability
- For clinical datasets or third party data, please ensure that the statement adheres to our policy

All sequencing data generated and/or used in this study are publicly available and listed in Extended Data Table 1 with their BioProject ID, accession # (if available), and/or URL. For convenience, we also list the BioProject IDs and/or URLs here: CHM1 whole-genome assembly with complete centromeres (PRJNA975207); CHM1 PacBio HiFi data (PRJNA726974); CHM1 ONT data (PRJNA869061); CHM1 Illumina data (PRJNA246220); CHM1 Strand-Seq alignments (https://doi.org/10.5281/zenodo.7959305); CHM1 CENP-A ChIP-seq data (PRJNA975217); T2T-CHM13 (v2.0) whole-genome assembly (PRJNA559484); CHM13 PacBio HiFi data (PRJNA530776); CHM13 ONT data (PRJNA559484); HG00733 PacBio HiFi data (PRJNA975575 and PRJEB36100); HG00733 ONT data (PRJNA975575, PRJNA686388, and PRJEB37264); HPRC whole-genome assemblies (https://projects.ensembl.org/hprc/); HGSVC whole-genome assemblies (https://www.internationalgenome.org/data-portal/data-collection/hgsvc2); and NHP [chimpanzee (Clint; S006007), orangutan (Susie; PR01109), and macaque (AG07107)] PacBio HiFi and ONT data (PRJNA659034). The original karyotyping imaging data for the CHM1 cell line is available from the Stowers Original Data Repository (http://www.stowers.org/research/publications/libpb-2457).

Research involving human participants, their data, or biological material

Policy information about studies with human participants or human data. See also policy information about sex, gender (identity/presentation), and sexual orientation and race, ethnicity and racism.

Reporting on sex and gender	N/A
Reporting on race, ethnicity, or other socially relevant groupings	We report analyses of publicly available human genome sequencing data generated by the 1000 Genomes Project (https://www.internationalgenome.org/home) and their associated genetic ancestry information, as established and described by the 1000 Genomes Project (https://www.internationalgenome.org/category/population/).
Population characteristics	See above.
Recruitment	See above.
Ethics oversight	See above.

Note that full information on the approval of the study protocol must also be provided in the manuscript.

Field-specific reporting

Please select the one below that is the best fit for your research. If you are not sure, read the appropriate sections before making your selection.

☒ Life sciences ☐ Behavioural & social sciences ☐ Ecological, evolutionary & environmental sciences

For a reference copy of the document with all sections, see nature.com/documents/nr-reporting-summary-flat.pdf

Life sciences study design

All studies must disclose on these points even when the disclosure is negative.

Sample size	We generated complete sequence assemblies of each centromere in the human CHM1hTERT genome (n=23) as well as a subset of orthologous centromeres in a second human (HG00733; n=12), chimpanzee (Pan troglodytes; Clint; S006007; n=11), orangutan (Pongo abelii; Susie; PR01109; n=10), and macaque (Macaca mulatta; AG07107; n=10). We also analyzed whole-genome assemblies from diverse humans generated by the Human Pangenome Reference Consortium (HPRC) and Human Genome Structural Variation Consortium (HGSVC; n=56 genomes; n=112 haplotypes; n=580 completely assembled centromeres; and n=2,049 incompletely assembled centromeres). For phylogenetic tree construction of centromeric regions, we used 150 data points from each genome. For centromeric mutation rate computation, we used hundreds to thousands of data points from each genome.
Data exclusions	No data were excluded.
Replication	Computational experiments are deterministic and are, therefore, reproducible. Each wet-lab experiment was performed at least two independent times.
Randomization	Randomization is not applicable to this study because we did not perform any experiments where there are treatment and control groups that would necessitate randomization between the subjects.
Blinding	Blinding is not applicable to this study because we did not perform any experiments where there are treatment and control groups that would necessitate blinding.

Reporting for specific materials, systems and methods

We require information from authors about some types of materials, experimental systems and methods used in many studies. Here, indicate whether each material, system or method listed is relevant to your study. If you are not sure if a list item applies to your research, read the appropriate section before selecting a response.

Materials & experimental systems

n/a	Involved in the study	
☐	☒	Antibodies
☐	☒	Eukaryotic cell lines
☒	☐	Palaeontology and archaeology
☒	☐	Animals and other organisms
☒	☐	Clinical data
☒	☐	Dual use research of concern
☒	☐	Plants

Methods

n/a	Involved in the study	
☐	☒	ChIP-seq
☒	☐	Flow cytometry
☒	☐	MRI-based neuroimaging

Antibodies

Antibodies used	We used a mouse monoclonal anti-human CENP-A antibody (clone 3-19; Enzo, ADI-KAM-CC006-E) in the ChIP-seq experiments. We used a rabbit polyclonal anti-human CENP-C antibody (made in house) and a goat anti-rabbit IgG antibody conjugated to FITC (Sigma F0382) in the immuno-FISH experiments.
Validation	The anti-human CENP-A antibody was generated against a synthetic peptide consisting of amino acids 3-19 of human CENP-A, and mutation of this epitope in human cells prevents antibody binding (Logsdon et al., JCB, 2015).

Eukaryotic cell lines

Policy information about cell lines and Sex and Gender in Research

Cell line source(s)	The human CHM1hTERT cell line was a gift from Urvashi Surti (Pittsburgh, PA). The human HG00733 lymphoblastoid cell line was obtained from the Coriell Institute for Medical Research (Camden, NJ). Chimpanzee (Pan troglodytes; Clint; S006007) fibroblast cells were obtained from a male western chimpanzee named Clint (now deceased) at the Yerkes National Primate Research Center (Atlanta, GA) and immortalized with EBV. Orangutan (Pongo abelii; Susie; PR01109) fibroblast cells were obtained from a female Sumatran orangutan named Susie (now deceased) at the Gladys Porter Zoo (Brownsville, TX), immortalized with EBV, and stored at the Coriell Institute for Medical Research (Camden, NJ). Macaque (Macaca mulatta; AG07107) fibroblast cells were originally obtained from a female rhesus macaque of Indian origin and stored at the Coriell Institute for Medical Research (Camden, NJ).
Authentication	The human CHM1hTERT cell line was authenticated via STR analysis by Cell Line Genetics (Madison, WI). The human HG00733 cell line is part of the NHGRI Sample Repository for Human Genetic Research at the Coriell Institute for Medical Research (Camden, NJ) and was authenticated using a multiplex PCR assay with six autosomal microsatellite markers. The chimpanzee, orangutan, and macaque cell lines have not yet been authenticated to our knowledge.
Mycoplasma contamination	The human CHM1hTERT and HG00733 cell lines are negative for mycoplasma contamination. The chimpanzee, orangutan, and macaque cell lines have not yet been tested for mycoplasma contamination to our knowledge.
Commonly misidentified lines<br>(See ICLAC register)	No commonly misidentified cell lines were used in this study.

Plants

Seed stocks	*Report on the source of all seed stocks or other plant material used. If applicable, state the seed stock centre and catalogue number. If plant specimens were collected from the field, describe the collection location, date and sampling procedures.*
Novel plant genotypes	*Describe the methods by which all novel plant genotypes were produced. This includes those generated by transgenic approaches, gene editing, chemical/radiation-based mutagenesis and hybridization. For transgenic lines, describe the transformation method, the number of independent lines analyzed and the generation upon which experiments were performed. For gene-edited lines, describe the editor used, the endogenous sequence targeted for editing, the targeting guide RNA sequence (if applicable) and how the editor was applied.*
Authentication	*Describe any authentication procedures for each seed stock used or novel genotype generated. Describe any experiments used to assess the effect of a mutation and, where applicable, how potential secondary effects (e.g. second site T-DNA insertions, mosiacism, off-target gene editing) were examined.*

ChIP-seq

Data deposition

☒ Confirm that both raw and final processed data have been deposited in a public database such as GEO.

☒ Confirm that you have deposited or provided access to graph files (e.g. BED files) for the called peaks.

Data access links<br>*May remain private before publication.*	https://www.ncbi.nlm.nih.gov/sra/?term=SRR24675260<br>https://www.ncbi.nlm.nih.gov/sra/?term=SRR24675261<br>https://www.ncbi.nlm.nih.gov/sra/?term=SRR24675262<br>https://www.ncbi.nlm.nih.gov/sra/?term=SRR24675263
Files in database submission	CHM1_CA_ChIP_1_S3_R1_001.fastq.gz<br>CHM1_CA_ChIP_1_S3_R2_001.fastq.gz<br>CHM1_CA_ChIP_2_S4_R1_001.fastq.gz<br>CHM1_CA_ChIP_2_S4_R2_001.fastq.gz<br>CHM1_Input_1_S1_R1_001.fastq.gz<br>CHM1_Input_1_S1_R2_001.fastq.gz<br>CHM1_Input_1_S2_R1_001.fastq.gz<br>CHM1_Input_1_S2_R2_001.fastq.gz
Genome browser session<br>(e.g. UCSC)	No longer applicable.

Methodology

Replicates	Two independent replicates of CENP-A ChIP-seq (with chromatin input as a control) were performed on CHM1hTERT cells and were in agreement with each other.
Sequencing depth	The total number of reads generated from each CHM1hTERT CENP-A ChIP-seq experiment is as follows:<br><br>CHM1hTERT CENP-A ChIP (Replicate 1): 113,284,073 paired-end, 150x150-bp reads<br>CHM1hTERT CENP-A ChIP (Replicate 2): 82,612,743 paired-end, 150x150-bp reads<br>CHM1hTERT Input (Replicate 1): 81,452,960 paired-end, 150x150-bp reads<br>CHM1hTERT Input (Replicate 2): 90,430,891 paired-end, 150x150-bp reads
Antibodies	We used a mouse monoclonal anti-human CENP-A antibody (clone 3-19; Enzo, ADI-KAM-CC006-E) to enrich for CENP-A-containing chromatin in the CHM1hTERT cell line.
Peak calling parameters	We aligned the CHM1hTERT CENP-A ChIP and input sequencing data to the CHM1hTERT whole-genome assembly generated in this study using BWA-MEM (v0.7.17) with the following parameters: bwa mem -k 50 -c 1000000 {index} {read1.fastq.gz} {read2.fastq.gz}. The resulting SAM files were filtered using SAMtools (v1.9) with flag score 2308 to prevent multi-mapping of reads. With this filter, reads mapping to more than one location are randomly assigned a single mapping location, thereby preventing mapping biases in highly identical regions. Alignments were normalized with deepTools (v3.4.3) bamCompare with the following parameters: bamCompare -b1 {ChIP.bam} -b2 {bulk_nucleosomal.bam} --operation ratio --binSize 1000 minMappingQuality 1 -o {out.bw}.
Data quality	The CHM1hTERT CENP-A ChIP and input sequencing data were assessed for quality using FastQC (https://github.com/s-andrews/FastQC), trimmed with Sickle (v1.33; https://github.com/najoshi/sickle) to remove low-quality 5' and 3' end bases, and trimmed with Cutadapt (v1.18) to remove adapters.
Software	BWA-MEM (v0.7.17) was used to align the CHM1hTERT CENP-A ChIP and input sequencing data to the CHM1hTERT whole-genome assembly. SAMtools (v1.9) was used to remove multi-mapped reads, and deepTools (v3.4.3) bamCompare was used to normalize and filter CENP-A ChIP data relative to input data to calculate fold enrichment.
